# The roles of CPSF6 in proliferation, apoptosis and tumorigenicity of lung adenocarcinoma

**DOI:** 10.18632/aging.204407

**Published:** 2022-11-29

**Authors:** Yukun Zu, Dao Wang, Wei Ping, Wei Sun

**Affiliations:** 1 Department of Thoracic Surgery, Tongji Hospital, Tongji Medical College, Huazhong University of Science and Technology, Wuhan 430030, Hubei Province, China

**Keywords:** lung adenocarcinoma, CPSF6, proliferation, apoptosis, tumorigenesis

## Abstract

Cleavage and polyadenylation specific factor 6 (CPSF6), a member of serine/arginine-rich protein family, is implicated in HIV-1-infection and replication. Overexpression of CPSF6 predicts poor prognostic outcomes of breast cancer. However, the expression and possible function of CPSF6 in lung adenocarcinoma (LUAD) still needs to be explored. Here, we found that CPSF6 is significantly higher expressed in tumor tissues than normal tissues in multiple cancer types. Besides, CPSF6 plays a significant risky role in LUAD that is associated with overall survival (HR=1.337, P=0.051) and disease-specific survival (HR=1.4739, P=0.042). CPSF6 mRNA was up-regulated in LUAD tissues by analyzing publicly available datasets from Gene Expression Omnibus (GEO). Further survival analysis on The Cancer Genome Atlas (TCGA) dataset suggested a close correlation between CPSF6 expression and overall survival, and disease-free survival of LUAD patients. Inhibition of CPSF6 expression by lentivirus-mediated RNA interference (RNAi) in two LUAD cell lines (A549 and NCH-H1299) caused a significant reduction in cell proliferation, colony formation and a notable induction in apoptotic rate. CPSF6 knockdown in xenograft tumors inhibited LUAD cell growth *in vivo*. Moreover, we identified differentially expressed genes with CPSF6 inhibition by Microarray analysis, and pathway analyses revealed that CPSF6 knockdown resulted in the dysregulation of the phosphatidylinositol 3-kinase/protein kinase B (PI3K/AKT) pathway. Collectively, our results are the first to demonstrate that CPSF6 functions as an oncoprotein by regulating cancer-related pathways in LUAD.

## INTRODUCTION

Lung cancer is one of the most frequent malignancies and the main cause of cancer mortality globally [1, 2]. The major type of lung cancer is non-small-cell lung carcinoma (NSCLC), among which lung adenocarcinoma (LUAD) is the main histological type [3]. Despite decades of efforts to improve outcome, the prognosis for lung cancer is still unfavorable, especially for advanced NSCLC. The 5-year survival rate of NSCLC remains less than 15% [4]. Undoubtedly, there is an urgency to elucidate the molecular mechanisms involved in the pathogenesis and progression of LUAD. The expression of components of several cancer-related signaling pathways, such as Mammalian Target of Rapamycin (MTOR) [5, 6], PI3K/AKT [7, 8], Insulin-like Growth Factor-1 (IGF-1) [9] and Epidermal Growth Factor Receptor (EGFR) [10, 11] pathways are frequently disturbed in LUAD. Molecular target therapy has become important parts of LUAD treatment [12, 13].

Cleavage and polyadenylation specific factor 6 (CPSF6), belonging to the serine/arginine-rich (SR) protein family, is a pre-mRNA splicing factor. CPSF6 was first identified as a component of cleavage factor Im (CFIm) complex [14, 15], which plays critical role in mRNA 3’ end processing and alternative polyadenylation [16, 17]. The death and proliferation of tumor cells are important pathological processes in tumor progression. Liu et al. found that CPSF6 inhibited the BTG2 expression to promote glycolysis and suppress apoptosis in HCC cells by activating AKT/ERK/NF-κB pathway [18]. Furthermore, Previous studies have revealed that CPSF6 helps the nuclear entry, integration and replication of HIV-1 [19, 20] and suppresses HIV-1-induced innate immunity in monocyte-derived macrophages (MDMs) [21]. Recently, Binothman et al. has reported that CPSF6 is overexpressed in breast cancer and correlated with poor patient outcomes [15]. They further suggest that CPSF6 serves as an oncogene in aggressive breast cancer. Mechanismly, their study demonstrated that CPSF6, residing within the nucleoplasm, is a key component of the pro-oncogenic adenosine-to-inosine (A-to-I) RNA editing process through interactions with paraspeckles and ADAR1. However, the expression profile and biological function of CPSF6 on other types of cancer still needs to be addressed.

In the current study, the expression of CPSF6 in NSCLC tissues and its prognostic value for survival of patients with LUAD were analyzed with public available dataset. To clarify the benefits of CPSF6 inhibition in LUAD, we knocked down the expression of the CPSF6 gene in two human LUAD cell lines, A549 and NCI-H1299, by lentivirus-mediated RNA interference (RNAi) and evaluated the alterations in lung cancer proliferation and apoptosis. Further, we identified differentially expressed genes (DEGs) with CPSF6 inhibition by Microarray analysis and established the pathways and associated protein networks involving in the identified DEGs, which provided comprehensive information on molecular mechanism of LUAD development involving CPSF6.

## RESULTS

### Pan-cancer analysis of CPSF6

**Figure 1:**
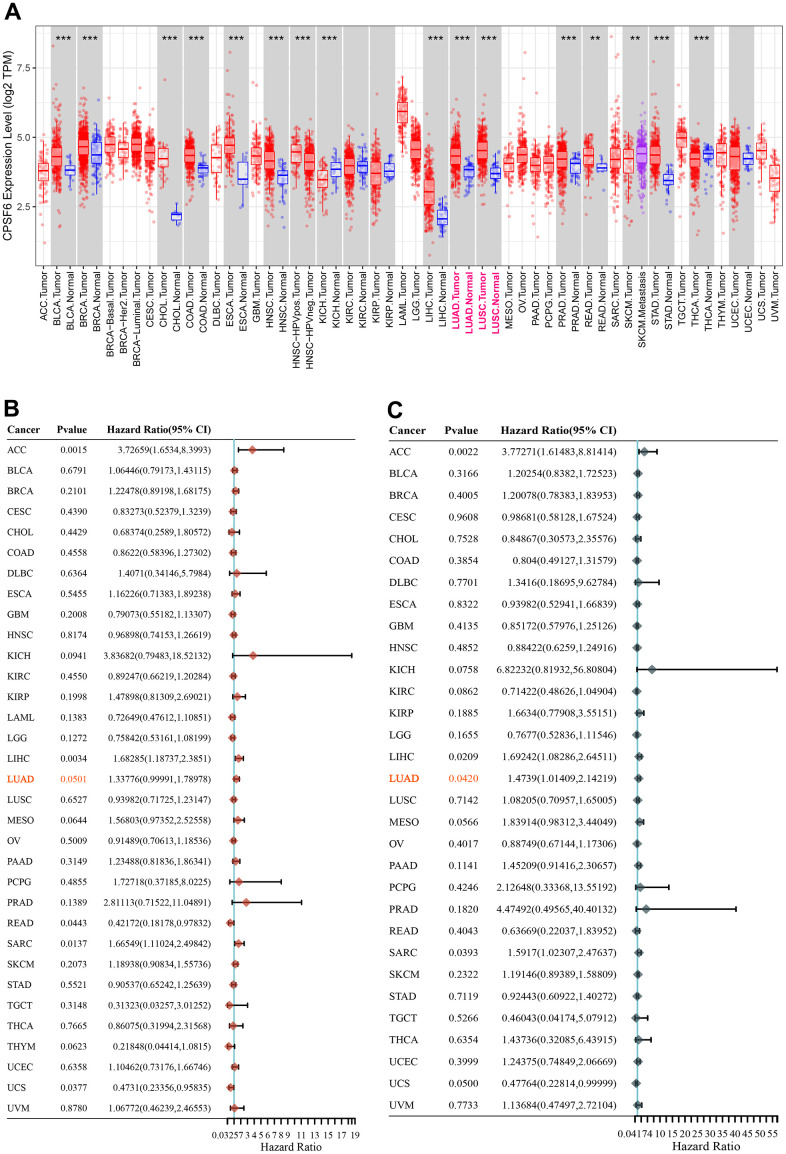
**Pan-cancer analysis of CPSF6.** (**A**) Differential expression status of CPSF6 in pan-cancer, ^***^P<0.001. (**B**) Hazard ratio of CPSF6 about overall survival in pan-cancer. (**C**) Hazard ratio of CPSF6 about disease specific survival in pan-cancer.

CPSF6 is significantly higher expressed in tumor tissues than normal tissues in multiple cancer types, especially in both LUAD and lung squamous cell carcinoma (LUSC) (Figure 1A). Besides, CPSF6 plays a significant risky role in LUAD that is associated with overall survival (HR=1.337, P=0.051, Figure 1B) and disease-specific survival (HR=1.4739, P=0.042, Figure 1C).

### CPSF6 overexpression was correlated with poor overall survival of LUAD patients

**Figure 2:**
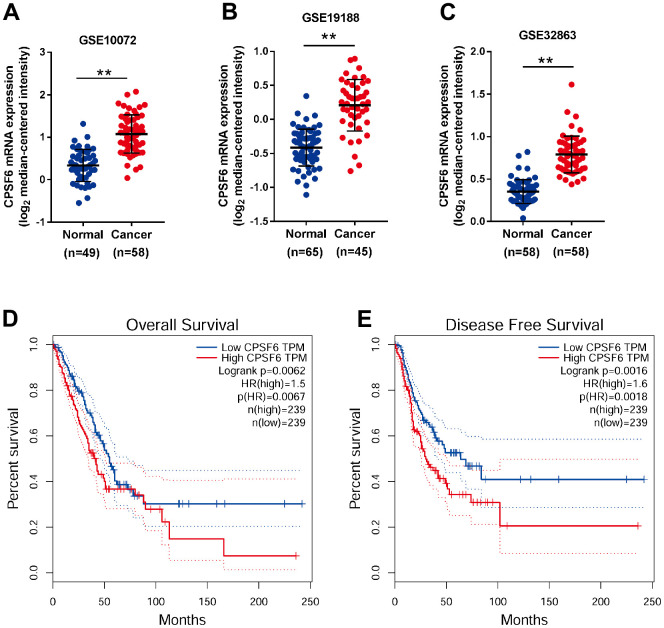
**CPSF6 overexpression was correlated with poor overall survival of LUAD patients.** (**A**–**C**) Comparison of CPSF6 mRNA expression in NSCLC and normal lung tissues from GSE10072 (**A**), GSE19188 (**B**) and GSE32863 (**C**). (**D**, **E**) Overall survival (**D**) and disease-free survival (**E**) analysis of TCGA LUAD dataset. **P<0.01.

Three GEO datasets, including GSE10072 [22], GSE19188 [23] and GSE32863 [24], were downloaded for comparison of CPSF6 mRNA expression between NSCLC tissue specimens and normal lung tissue samples. The results of two-sided Student’s t-test indicated that CPSF6 mRNA levels were significantly elevated in NSCLC tissues (Figure 2A–2C, P<0.01).

By analyzing RNA-sequencing datasets of LUAD, the main histological type of NSCLC, from TCGA, we found that CPSF6 overexpression was correlated with the poor overall survival (Figure 2D) and disease-free survival of patients with LUAD (Figure 2E). These data suggest that CPSF6 overexpression may be a novel diagnosis and prognosis marker for LUAD, and that CPSF6 might be involved in LUAD development.

### CPSF6 knockdown inhibited LUAD cell growth *in vitro*

**Figure 3:**
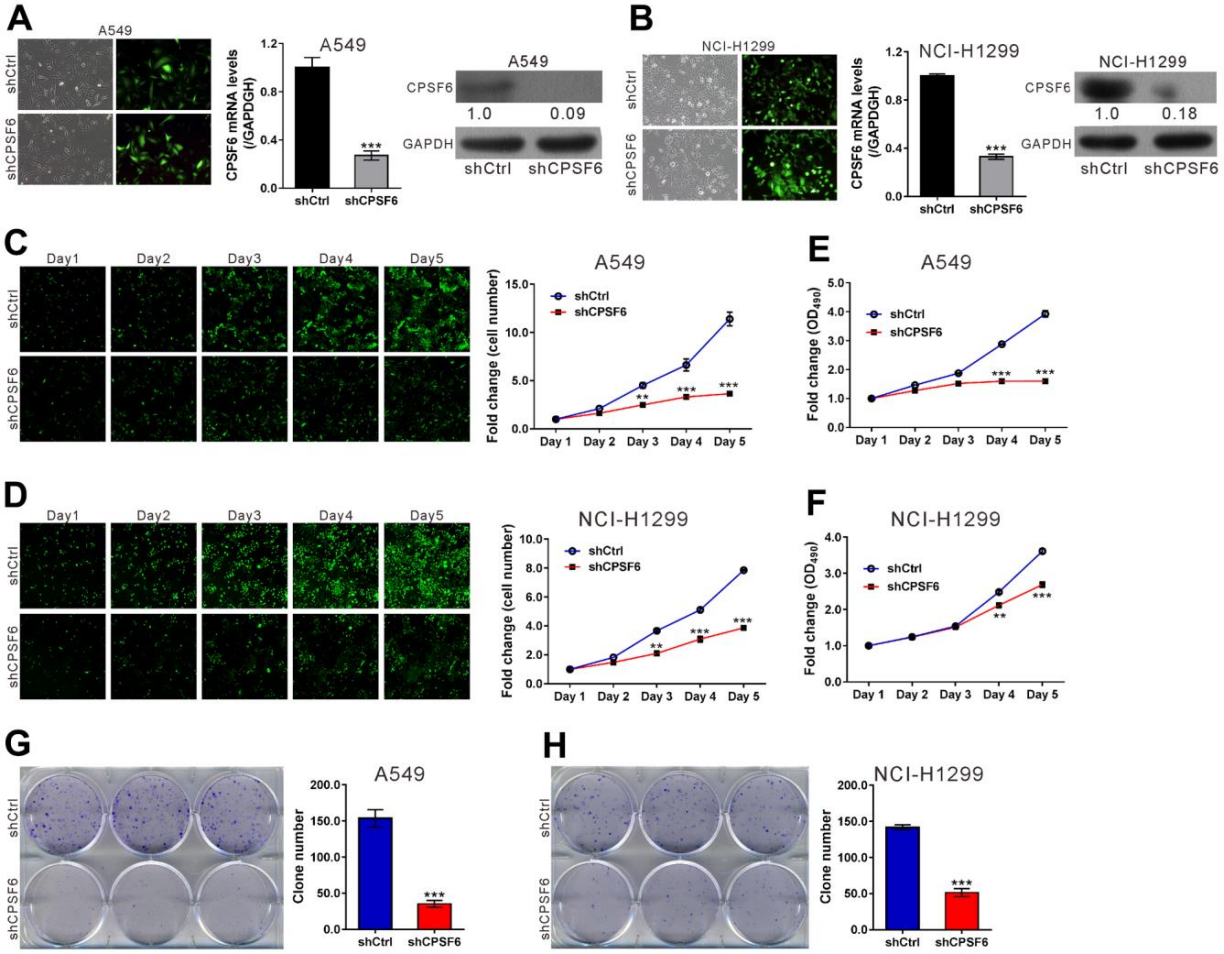
**CPSF6 knockdown suppressed cell proliferation of LUAD cells.** A549 and NCI-H1299 cells were transduced with lentivirus expressing CPSF6 shRNA (shCPSF6) or control shRNA (shCtrl). (**A**, **B**) At 72 h later, infection efficiency was estimated by counting the numbers of GFP expressing cells under a fluorescence microscopy. Magnification: 200× (left panel). Real-time PCR analysis and western blotting were conducted to assess mRNA (middle panel) and protein expression (right panel) of CPSF6, respectively. The densitometric analysis of western blotting was shown below the blot. (**C**, **D**) The Celigo Cell Counting assay was done for 5 days. The fold change of cell number was calculated relative to Day 1. (**E**, **F**) MTT assay was used to estimate cell proliferation. The fold change of OD_490_ was calculated relative to Day 1. (**G**, **H**) Colony formation assay was continued for 8 days to evaluate cell proliferation. **P<0.01, ***P<0.001 versus shCtrl. The number of cell experiments in parallel (n=3).

To explore the functions of CPSF6 in LUAD cells, lentivirus expressing CPSF6 shRNA (shCPSF6) or control shRNA (shCtrl) was transduced into A549 (Figure 3A) and NCI-H1299 cells (Figure 3B). The infection efficiency of both viruses was estimated above 80%. Infection with shCPSF6 efficiently knocked down CPSF6 expression at both mRNA and protein levels.

Next, cell proliferation was assessed in A549 and NCI-H1299 cells with CPSF6 knockdown by Celigo Image cytometer assays, MTT assays and colony formation assays. The results of Celigo Image cytometer assays indicated that the fold change of cell number was significantly decreased in A549 and NCI-H1299 cells infected with shCPSF6 virus at Day 3 (A549 2.5±0.08 VS 4.51±0.31, NCI-H1299 2.11±0.14 VS 3.67±0.08), Day 4 (A549 3.32±0.2 VS 6.64±0.63, NCI-H1299 3.09±0.18 VS 5.11±0.16) and Day 5 (A549 3.66±0.13 VS 11.41±0.71, NCI-H1299 3.87±0.04 VS 7.85±0.05) (P<0.01 versus shCtrl, Figure 3C, 3D). The results of MTT assay also demonstrated that the growth of both LUAD cells was significantly inhibited at Day 4 (A549 1.6±0.06 VS 2.88±0.01, NCI-H1299 2.12±0.06 VS 2.49±0.04) and Day 5 (A549 1.6±0.06 VS 3.93±0.12, NCI-H1299 2.69±0.07 VS 3.62±0.07) (P<0.01 versus shCtrl, Figure 3E, 3F). Moreover, colony formation assays showed that the clone number in A549 and NCI-H1299 cells infected with shCPSF6 virus was decreased to 22.9% and 36.2% of those infected with shCtrl virus, respectively (P<0.001 versus shCtrl, Figure 3G, 3H). Collectively, these data demonstrated the inhibitory effects of CPSF6 knockdown in LUAD cell growth *in vitro*.

### CPSF6 knockdown induced LUAD cell apoptosis

**Figure 4:**
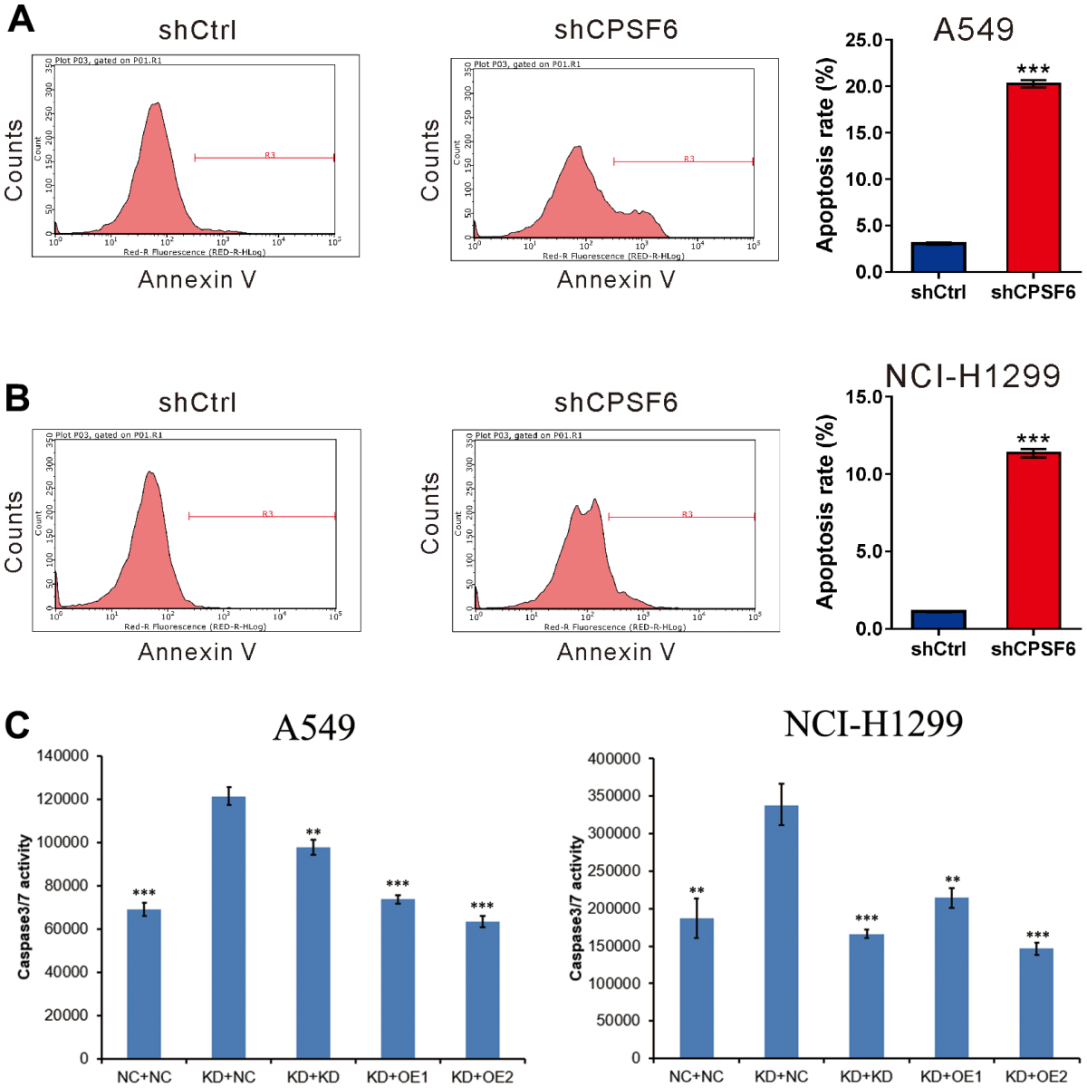
**CPSF6 knockdown induced apoptosis of LUAD cells.** A549 (**A**) and NCI-H1299 cells (**B**) were transduced with lentivirus expressing CPSF6 shRNA (shCPSF6) or control shRNA (shCtrl), labelled with the Annexin V Apoptosis Detection kit, and analyzed on a flow cytometry. Representative images and the apoptotic rates of three independent experiments are shown. ***P<0.001 versus shCtrl. (**C**). Caspase3/7 activity of A549 and NCI-H1299 cells were detected between five groups after transducing with lentivirus for 5 days. Control cell + control shRNA (NC+NC), CPSF6 shRNA + control shRNA (KD+NC), CPSF6 shRNA + GSK3B shRNA (KD+KD), CPSF6 shRNA + IRS1 Over Expressed (KD+OE1), CPSF6 shRNA + JUN Over Expressed (KD+OE2). **P<0.01, ***P<0.001 versus KD+NC. The number of cell experiments in parallel (n=3).

The results of Annexin V staining plus flow cytometry analysis (Figure 4A, 4B) revealed that the apoptotic ratio of A549 and NCI-H1299 cells infected with shCPSF6 virus was elevated 5.8-fold and 9.3-fold, respectively compared to shCtrl virus (P<0.001 versus shCtrl). In addition, the caspase3/7 activity of A549 and NCI-H1299 cells infected with shCPSF6 virus was elevated 1.76-fold and 1.8-fold compared to shCtrl virus, this change was reversed by infecting with shGSK3B, OE IRS1 or OE JUN respectively (Figure 4C). These results indicated that CPSF6 knockdown promoted LUAD cell apoptosis.

### CPSF6 knockdown suppressed LUAD cell growth *in vivo*

**Figure 5:**
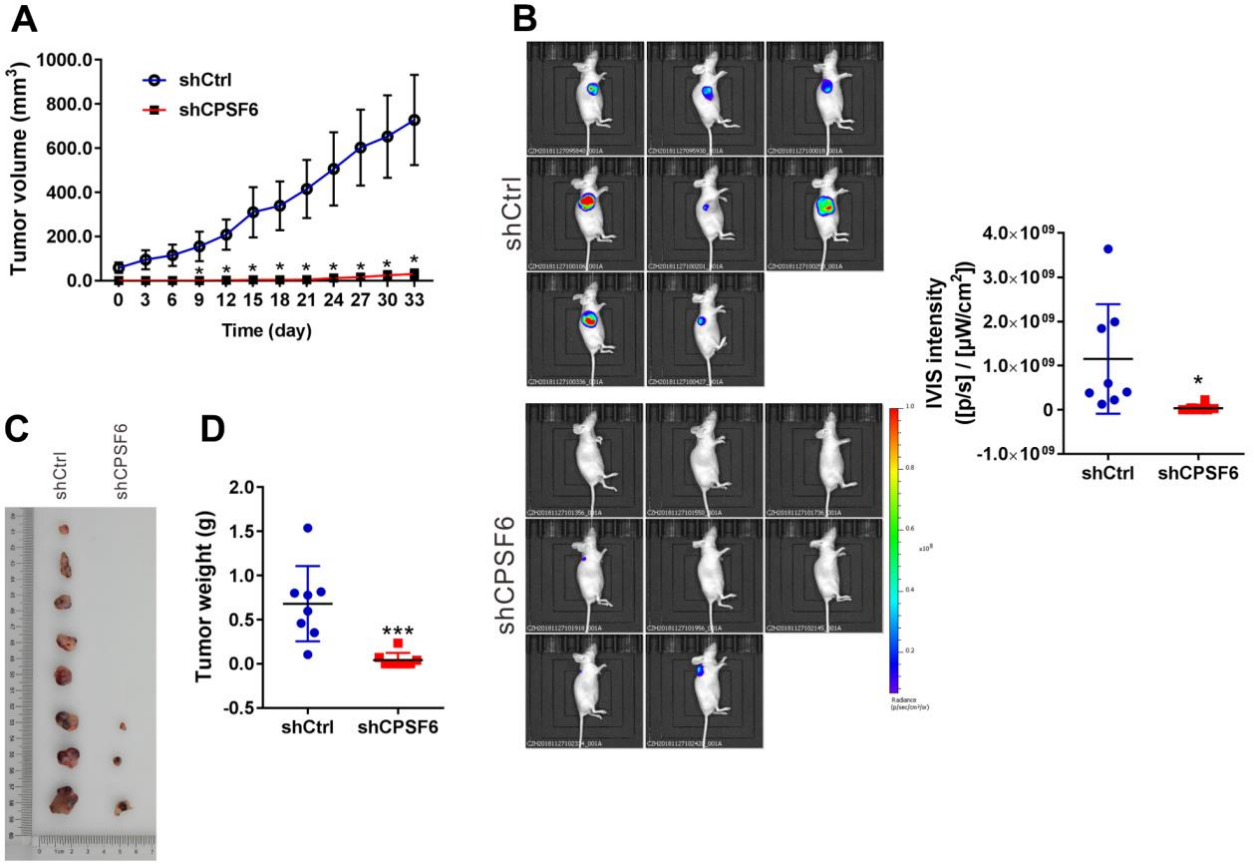
**CPSF6 knockdown suppressed LUAD cell growth *in vivo*.** Nude mice were transplanted with A549 cells stably expressing CPSF6 shRNA (shCPSF6) or control shRNA (shCtrl) (n=8 per group). (**A**) One month later, xenograft was formed, and the tumor volume of xenografts was then monitored every 3 days and continued for 33 days. (**B**) Images of tumor-bearing mice and quantitative IVIS intensity are shown (n=8 per group). (**C**, **D**) Xenografts were formed in 8 mice of shCtrl group and 3 mice of shCPSF6 group. These xenografts were collected (**C**) and weighed (**D**). *P < 0.05 and ***P<0.001 versus shCtrl.

To study the effects of CPSF6 knockdown *in vivo*, a xenograft mouse model was constructed by transplantation with A549 cells expressing shCtrl or shCPSF6. As shown in Figure 5A, tumor growth was significantly suppressed in shCPSF6 group (P<0.05 versus shCtrl). At the end of experiments, tumor formation rate of the shCtrl group and the shCPSF6 group was 100% (8/8) and 37.5% (3/8), respectively. *In vivo* imaging and intensity analysis (Figure 5B) demonstrated that the luciferase signals were significantly reduced in shCPSF6 group compared with the shCtrl group (3.73*10^7^±7.8*10^7^ VS 1.15*10^9^±1.24*10^9^, P<0.05). As shown in Figure 5C, 5D, the shCPSF6 group had significantly reduced tumor size and weight. These data demonstrated the inhibitory effects of CPSF6 knockdown on tumor progression *in vivo*.

### Analysis of CPSF6-regulated molecules

Microarray analysis was performed on A549 cells expressing shCtrl and shCPSF6. We identified a total of 1,268 DEGs with 456 up-regulations and 812 down-regulations (Supplementary Table 2 and Supplementary Figure 1) in the shCPSF6 group, when compared with the shCtrl group, based on the following criteria: FDR<0.05 and fold change>2.0.

**Figure 6:**
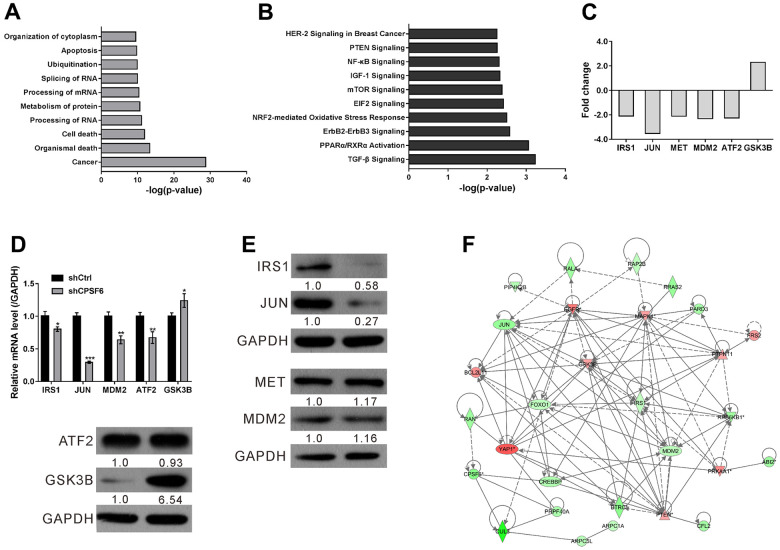
**Microarray analysis of A549 cells with CPSF6 knockdown.** A549 cells were transduced with lentivirus expressing CPSF6 shRNA (shCPSF6) or control shRNA (shCtrl). (**A**, **B**) Disease and function enrichment analyses (**A**) and canonical pathway analyses (**B**) with the IPA application. (**C**) Fold changes of IRS1, JUN, MET, MDM2, ATF2 and GSK3B as indicated by Microarray analysis. (**D**, **E**) Real-time PCR (**D**) and Western blot (**E**) was performed to validate Microarray data. The densitometric analysis of western blotting was shown below the blot. (**F**) CPSF6 knockdown-induced changes in the network constructed by IPA. Up-regulated and down-regulated Genes were presented in red and in green, respectively. *P < 0.05, **P < 0.01, ***P < 0.001 versus shCtrl. The number of cell experiments in parallel (n=3).

Disease and function enrichment analyses showed that “Cancer” and “apoptosis” ranked in the ten most significant categories (Figure 6A). Further IPA pathway analyses indicated that multiple cancer-associated pathways, such as transforming growth factor-β (TGF-β), mTOR, IGF-1, nuclear factor-κB (NF-κB) and phosphatase and tensin homolog (PTEN) pathways, were correlated with CPSF6 expression (Figure 6B).

To confirm the results of Microarray, key molecules associated with the above pathways (Insulin receptor substrate 1(IRS1), Jun proto-oncogene (JUN), Mesenchymal epithelial transition (MET), MDM2 proto-oncogene (MDM2), Activating transcription factor 2 (ATF2), and Glycogen synthase kinase 3 beta (GSK3B)) were assessed for verification. In line with the results of Microarray (Figure 6C), Real-time PCR (Figure 6D) demonstrated that IRS1 (0.80-fold), JUN (0.30-fold), MDM2 (0.64-fold) and ATF2(0.67-fold) was down-regulated, while GSK3B (1.24-fold) was up-regulated in the shCPSF6 group. Western blot demonstrated the down-regulation of IRS1 and JUN and the up-regulation of GSK3B (Figure 6E). Western blot demonstrated that p-AKT was down-regulated in the shCPSF6 group, while PI3K was not statistically different between the two groups (Supplementary Figure 2). Finally, based on the bioinformatics results and subsequent verification data, the correlation between CPSF6 and cancer-related genes were established by IPA (Figure 6F). There is a significant correlation between the expression of the screened key molecule mRNA (ATF2, GSK3B, IRS1, JUN, MDM2) and the expression of CPSF6 mRNA in TCGA datasets, except MET (Supplementary Figure 3). In addition, the expression of CPSF6 was also found to be significantly correlated with the expression of the Bcl-2, Bcl-2L1, Mcl-1 by GEPIA datasets (Supplementary Figure 4).

## DISCUSSION

As an important subunit of a cellular complex required for maturation of messenger RNAs (mRNA) from pre-mRNA, CPSF6 can directly activate the mRNA 3′-processing machinery and also involved in the mRNA nuclear export [25]. CPSF6 has been reported to be involved in several steps of the HIV-1 life cycle including cytoplasmic transport, nuclear import, and integration site selection [26]. In additional, overexpression of CPSF6 shows prognostic value in breast cancer. CPSF6 is able to regulate A-to-I RNA editing process and tumorigenesis in breast cancer [15]. We analyzed the differentially expression status of CPSF6 between normal tissue and tumor tissue in pan-cancer is retrieved from TIMER database, we found that CPSF6 is significantly higher expressed in tumor tissues than normal tissues in multiple cancer types, especially in both LUAD and LUSC. We also proposed that CPSF6 may be involved in the tumorigenesis of LUAD. To test this hypothesis, we first analyzed the mRNA expression of CPSF6 in GEO NSCLC datasets and revealed that CPSF6 levels were significantly elevated in NSCLC cancer tissues in comparison with normal lung tissues. Subsequently, survival analyses with TCGA dataset showed that increased CPSF6 expression had significant association with overall survival and disease-free survival of LUAD, the main histological type of NSCLC. Our data suggest that CPSF6 may serve as a novel diagnosis and prognosis marker for LUAD, but comprehensive analysis with a large number of clinical cases is required.

To explore the possible functions of CPSF6, we performed CPSF6 loss-of-function studies in two LUAD cell lines. The *in vitro* and *in vivo* experiments showed that blockage of CPSF6 expression resulted in a significant inhibition on LUAD cell proliferation. Thus, we, for the first time, provided evidence supported the oncogenic role of CPSF6 in LUAD. Dysregulated cell proliferation may be attributed to the inhibition of cell apoptosis during carcinogenesis [27]. We also observed elevated apoptotic rate in LUAD cells with CPSF6 knockdown. Guo et al. also reported depletion of CPSF6 led to cell cycle arrest and apoptosis in esophageal squamous cell carcinoma [28]. Thus, we supposed that the increased apoptosis was one of the potential causes of the reduced proliferation in LUAD cells with CPSF6 knockdown.

To further understand the biological significance of CPSF6 in the progression of LUAD, microarray analyses were performed and the DEGs between A549 cells with CPSF6 knockdown and control cells were identified. The results of disease and function analysis showed that a large number of DEGs were enriched in the categories of “Cancer” and “apoptosis”, which was consistent with the above functional data. These findings indicated that CPSF6 participates in cancer development via regulating cell apoptosis. CPSF6 has been found to play an important role in tumorigenesis and progression such as gastric cancer and hepatocellular carcinoma [29, 30].

Moreover, pathway analyses showed that the DEGs were involved in multiple cancer-associated pathways, such as TGF-β, mTOR, IGF-1, NF-κB and PTEN pathways in LUAD. The phosphatase PTEN negatively regulates AKT, while AKT activity is dependent on the activity of PI3K [31]. The PI3K/AKT signaling pathway, an important mechanism for tumorigenesis, is frequently dysregulated in many human cancers including LUAD [6–8]. Fundamental cellular functions, including proliferation, apoptosis, differentiation, cell repair and metabolism, are reported to be regulated by the PI3K/AKT signaling pathway through a variety of downstream effectors [2]. Here, the changes of three key molecules (IRS1, JUN and GSK3B) in the cancer-associated pathways were verified by real-time PCR and Western blotting. IRS1 plays a key role in oncogenic IGF-1 signaling, which activates the PI3K/AKT signaling [32]. JUN and GSK3B act downstream of the PI3K/AKT signaling pathway [31]. JUN is a critical mediator for the regulation of cell-cycle and apoptosis [33]. GSK3B regulates various cellular processes, including gene expression, proliferation and apoptosis [34]. Therefore, the changes of IRS1, JUN and GSK3B in LUAD cells with CPSF6 knockdown in the current study indicate a close correlation between CPSF6 and the PI3K/AKT pathway during LUAD tumorigenesis.

However, there are still some limitations in this study. First, the CPSF6 mRNA levels elevated in NSCLC tissues were obtained from TCGA, and it would be better if we could verify CPSF6 levels with LUAD tissue from our own center. In addition, this is the first report on the oncogenic role of CPSF6 in LUAD, but its underlying mechanism remains to be further explored.

## CONCLUSIONS

In conclusion, blockage of CPSF6 suppressed the proliferation and induced the apoptosis of LUAD cells. The oncogenic function of CPSF6 may be ascribed to the regulation of key molecules in multiple signaling pathways, especially the PI3K/AKT pathway. Collectively, the current findings provide comprehensive information on the molecular mechanisms underlying the pathogenesis of LUAD.

## MATERIALS AND METHODS

### Pan-cancer analysis of CPSF6

Differentially expression status of CPSF6 between normal tissue and tumor tissue in pan-cancer is retrieved from TIMER database (https://cistrome.shinyapps.io/timer/), and the univariate cox regression were performed subsequently to investigate the prognostic value of CPSF6 in pan-cancer, especially in lung adenocarcinoma.

### Public datasets

Three datasets, GSE10072 [22], GSE19188 [23] and GSE32863 [24], were acquired from the GEO database (https://www.ncbi.nlm.nih.gov/geo/), and CPSF6 mRNA expression in NSCLC specimens and normal lung tissues were compared by Student’s t test. Survival data of LUAD patients were acquired from the TCGA project, and the correlation between CPSF6, overall survival and disease-free survival was analyzed. In addition, the correlation between the expression of CPSF6 mRNA and the expression of the screened key molecule mRNA (ATF2, GSK3B, IRS1, JUN, MDM2 and MET) is analyzed in TCGA datasets. The correlation between the expression of CPSF6 and the expression of the Bcl-2, Bcl-2L1, Mcl-1 have analyzed by GEPIA datasets.

### LUAD cell lines

NCI-H1299 and A549 cells were acquired from the Chinese Academy of Sciences (Shanghai, China). NCI-H1299 and A549 cells were grown in RPMI-1640 medium (Corning, New York, NY, USA) and F12K medium (Corning), respectively, with 10% fetal bovine serum (FBS; Ausbian, Australia). The cells were cultured in an incubator at 37° C with 95% air and 5% CO_2_.

### Real-time PCR

Total RNA was isolated using Trizol reagent (Pufei, Shanghai, China), and M-MLV Reverse Transcriptase (Promega, Madison, WI, USA) was used to reverse transcribe the total RNA into single-stranded cDNA as per manufacturer’s guidance. Real-time PCR was then conducted to determine the mRNA levels of interested genes with SYBR Master Mixture (Takara, Shanghai, China) and specific primers (Supplementary Table 1) on Agilent MX3000p qPCR instrument (Agilent, Palo Alto, CA, USA) according to manufacturer’s guidance. The PCR program was initiated at 95° C for 30 s, followed by 45 cycles of 95° C for 5 s and 60° C for 30 s. Relative gene expression was normalized to GAPDH using the 2-ΔΔCT method.

### Western blot

Cell extracts were prepared using RIPA buffer (Beyotime, Shanghai, China). Following separation by 10% SDS-PAGE, the cell extracts were blotted onto the polyvinylidene fluoride (PVDF) membranes (Millipore, Bedford, MA, USA). The membranes were then incubated with the following antibodies: anti-CPSF6 (Sigma, St. Louis, MO, USA; AV40697), anti-insulin receptor substrate 1 (IRS1) (Abcam, Cambridge, MA, USA; ab52167), anti-JUN (Abcam; ab32137), anti-MET (Abcam, ab51067), anti-MDM2 (Abcam, ab38618), anti-ATF2 (Abcam, ab32160) and anti-glycogen synthase kinase-3 (GSK3B) (Abcam, ab93926). Antibodies against GAPDH (Santa-Cruz, USA; sc-32233) were used as loading control. Following incubation with a horseradish peroxidase-conjugated secondary antibody (Cell Signaling Technology, Danvers, MA, USA), Pierce™ ECL Western Blotting Substrate (Thermo Fisher Scientific, Rockford, IL, USA) was applied to detect the immunoreactive proteins. Densitometric analysis was carried out with Image J software (National Institute of Health, Bethesda, MD, USA).

### Small hairpin RNAs (shRNAs) and lentiviruses

The polynucleotides of shRNA targeting human CPSF6 (shCPSF6, CATAGTAGATCACGAGAAA) and control shRNA (shCtrl, TTCTCCGAACGTGTCACGT) was synthesized, annealed, and cloned into the lentiviral vector GV115 containing GFP (green fluorescent protein) expression cassette (Genechem, Shanghai, China). Sequencing was conducted to verify the constructs. Lentivirus production was performed with 293T cells as previously described [35]. A549 and NCI-H1299 cells were infected with the lentivirus at the presence of 8 μg/ml polybrene. At 72 h later, the infection efficiency was determined by observing under a fluorescence microscopy (IX71; Olympus, Japan). To evaluate the knockdown efficiency, real-time PCR and western blotting analysis was conducted to compare the mRNA and protein expression of CPSF6 between cells infected with shCPSF6 and shCtrl, respectively.

### Analysis of cell proliferation using Celigo Image Cytometer

A549 cells and NCI-H1299 cells were infected with lentivirus expressing either shCPSF6 or shCtrl, seeded onto 96-well plates (A549, 1.5 × 10^3^/well; NCI-H1299, 1.0 × 10^3^/well) and cultured overnight. Subsequently, the Celigo Cell Counting application (Nexcelom Bioscience, Lawrence, MA, USA) was use to image and count cell number every day for 5 days. The fold change of cell number was obtained by dividing cell number at indicated time point to that at Day 1.

### MTT assay

A549 cells and NCI-H1299 cells were treated and plated as described above. After culturing overnight, MTT (3- [4, 5-dimethylthiazol-2-yl]-2, 5 diphenyl tetrazolium bromide) assay was done every day for 5 days. Fresh medium was replaced regularly. In brief, MTT solution (Genview, Houston, TX, USA) was added and incubated at 37° C for 4 h. The culture medium with MTT reagent was eliminated carefully and formazan was dissolved with DMSO. The optic density at 490 nm (OD490) was recorded by a microplate reader (Tecan Infinite M2009PR, Crailsheim, Germany). The fold change of OD490 was obtained by dividing OD490 at indicated time point to that at Day 1.

### Colony formation assays

A549 and NCI-H1299 cells were infected with lentivirus expressing shCPSF6 or shCtrl. After 3 days, the cells were seeded into 6-well plates at 1.0 × 103 cells/well. The cultured medium was changed every 3 days. After 8 days of culture, the colonies were fixed with 4% paraformaldehyde and enumerated by staining with 0.2% crystal violet and microscopy (XDS-100, Caikang, Shanghai, China).

### Cell apoptosis analysis

A549 and NCI-H1299 cells were infected with lentivirus expressing either shCPSF6 or shCtrl. After 3 days, the cells were plated into 6 cm dishes. After another 2 days, the cells were collected, and probed with Annexin V-APC (eBioscience, San Diego, CA, USA) at room temperature for 10 min as per the manufacturer’s guidance. The labelled cells were evaluated by flow cytometry (Millipore). A549 and NCI-H1299 cells were infected with lentivirus expressing either shCPSF6 or shCtrl or shGSK3B or OE IRS1 or OE JUN. After 5 days, the cells were detected by Caspase Glo® 3/7 Assay (Promega, Madison, WI, USA).

### Tumor xenograft assay

A total of 10^7^ A549 cells transduced with lentivirus expressing shCPSF6 or shCtrl were implanted subcutaneously into the right flanks of female BALB/c-A nude mice at 4 weeks of age (Shanghai Linchang Biological Technology Co., Shanghai, China). After one month, tumor length and width were monitored every three days. Tumor volumes were calculated as π/6×length×width2 (mm3). At 33 days, tumors were detected by a PerkinElmer imaging system (Lumina LT, Tübingen, Germany), excised, and weighted.

### Microarray analysis and data processing

Total RNA was extracted from A549 cells infected with lentivirus expressing either shCPSF6 or shCtrl as described above. RNA integrity was assessed by NanoDrop 2000 (Thermo Fisher Scientific) and Agilent Bioanalyzer 2100 (Agilent Technologies Inc., Santa Clara, CA, USA). The RNA was amplified and Biotin-modified with GeneChip 3’IVT PLUS Kit (Affymetrix Inc., Santa Clara, CA, USA) to generate aRNA. The aRNA was fragmented and hybridized onto PrimeView Human Gene Expression Array (Affymetrix Inc.) according to the manufacturer’s guidance. GeneChip scanner 3000 (Affymetrix Inc.) was used to scan Microarray signals.

Differentially expressed genes (DEGs) were identified according to the following criteria: fold change > 2.0 and false discovery rate (FDR) < 0.05. The canonical pathways and associated protein networks involving in the identified DEGs were then established by Ingenuity Pathway Analysis (IPA) 9.0 (Ingenuity Systems, Redwood City, CA, USA).

### Statistical analyses

Data were analyzed with Prism GraphPad 7.0 (GraphPad Software, Inc, San Diego, CA, USA). Data of *in vitro* experiments are shown as mean ± standard deviation (SD) of three independent experiments. The two-sided Student’s t-test used to determine the statistical difference between two groups and a P value of <0.05 represents statistical significance.

## Supplementary Material

Supplementary Figures

Supplementary Table 1

Supplementary Table 2
