# Supplementary material for: The roles of CPSF6 in proliferation, apoptosis and tumorigenicity of lung adenocarcinoma
Source: Aging (Albany NY). 2022 Nov 29;14(22):9300–16. doi: 10.18632/aging.204407 (PMC9740356; doi:10.18632/aging.204407)
Supplement: Supplementary Figures [file aging-14-204407-s001.pdf]

SUPPLEMENTARY FIGURES

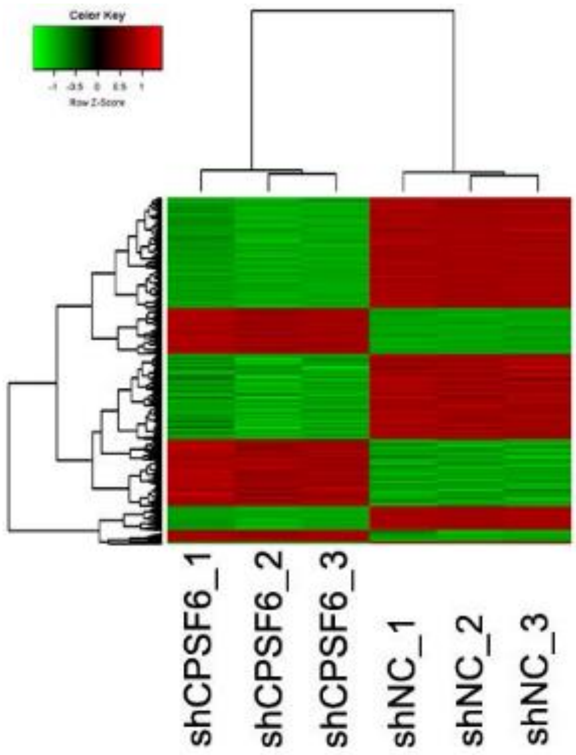

Supplementary Figure 1. The result of microarray analysis in A549 cells expressing shCtrl and shCPSF6.

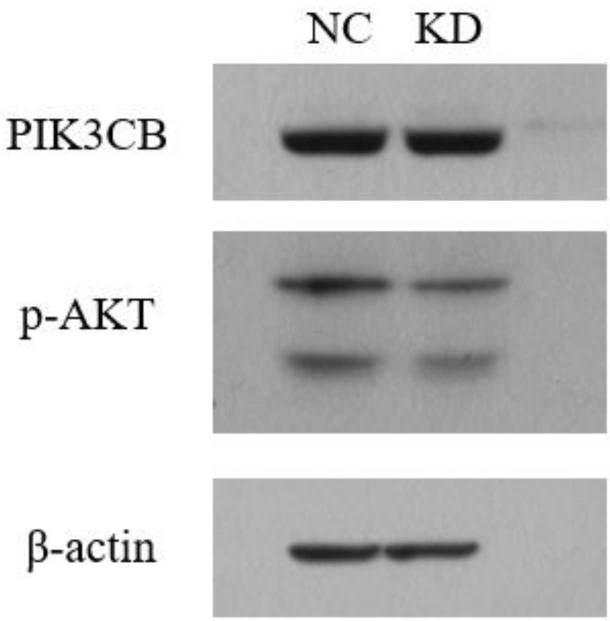

Supplementary Figure 2. The change of p-AKT and PI3K in A549 cells expressing shCtrl and shCPSF6 by western blot.

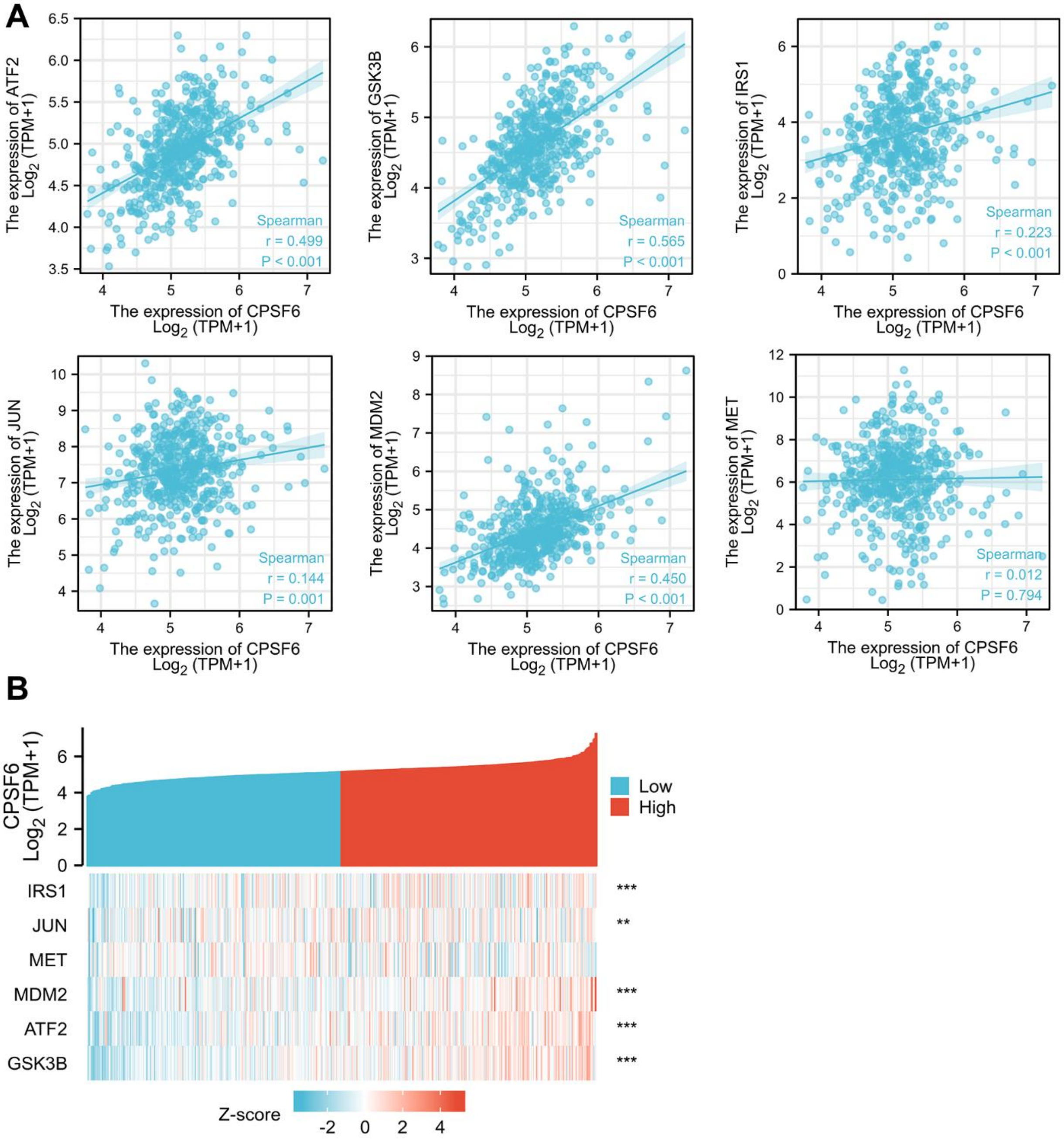

Supplementary Figure 3. The correlation between the expression of the screened key molecule mRNA (ATF2, GSK3B, IRS1, JUN, MDM2, MET) and the expression of CPSF6 mRNA in TCGA datasets.

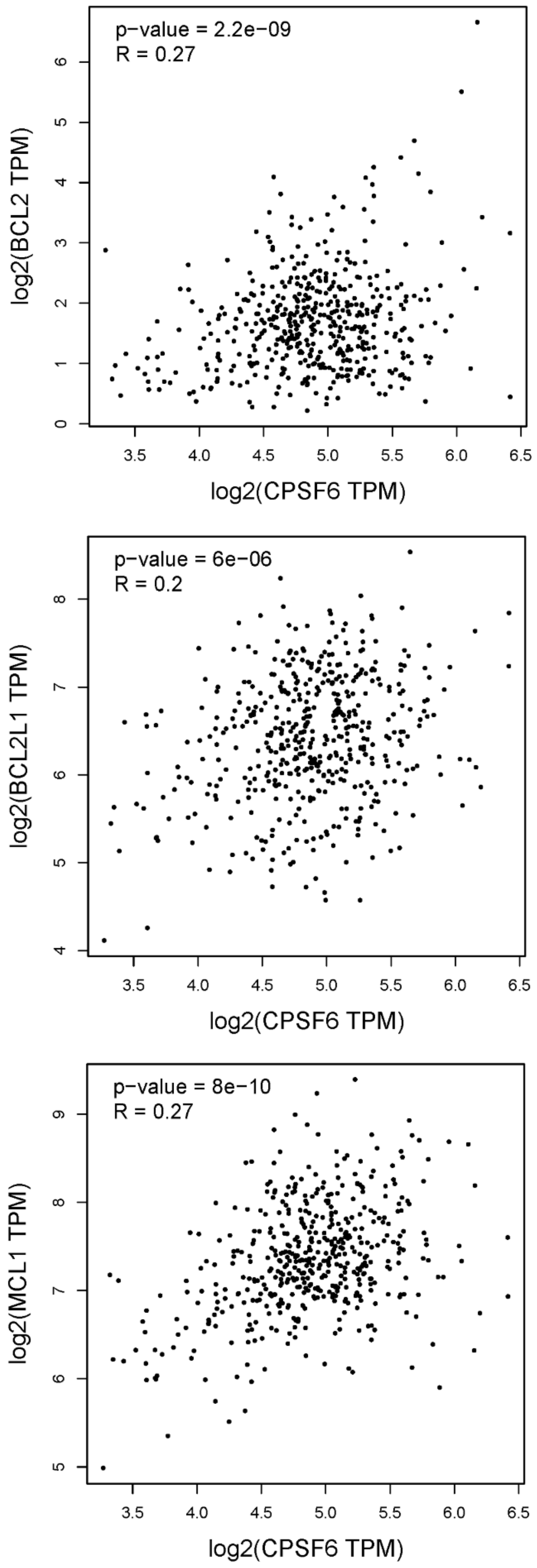

Supplementary Figure 4. The correlation between the expression of CPSF6 with the expression of the Bcl-2, Bcl-2L1, Mcl-1 by GEPIA datasets.
