# Supplementary material for: The roles of CPSF6 in proliferation, apoptosis and tumorigenicity of lung adenocarcinoma
Source: Aging (Albany NY). 2022 Nov 29;14(22):9300–16. doi: 10.18632/aging.204407 (PMC9740356; doi:10.18632/aging.204407)
Supplement: Supplementary Table 1 [file aging-14-204407-s002.pdf]

SUPPLEMENTARY TABLE

Supplementary Table 1. Primer sequences for real-time PCR.

Gene	Forward primer	Reverse primer	Size (bp)
CPSF6	TGGTGTTGGATCTGAAGCATC	CCCAGACATTTGTCCTGATTGT	160
GSK3b	GCACTGTGTAGCCGTCTG	GAGGAGGAATAAGGATGGTAGC	199
MDM2	GAATCATCGGACTCAGGTACATC	TCTGTCTCACTAATTGCTCTCCT	167
IRS1	ACAAACGCTTCTTCGTACTGC	AGTCAGCCCGCTTGTTGATG	156
JUN	ATGGTCAGGTTATACTCCTCCTC	CACATGCCACTTGATACAATCC	164
ATF2	GTCATGGTAGCGGATTGGTTA	CTTTGGGTCTGTGGAGTTGTG	124
GAPDH	TGACTTCAACAGCGACACCCA	CACCCTGTTGCTGTAGCCAAA	121
