# Supplementary material for: The roles of CPSF6 in proliferation, apoptosis and tumorigenicity of lung adenocarcinoma
Source: Aging (Albany NY). 2022 Nov 29;14(22):9300–16. doi: 10.18632/aging.204407 (PMC9740356; doi:10.18632/aging.204407)
Supplement: Supplementary Table 2 [file aging-14-204407-s003.docx]

| **Supplementary Table 2.** Identified differentially expressed genes. | | | | | | | |
| --- | --- | --- | --- | --- | --- | --- | --- |
| **Entrez** | **Gene Symbol** | **Fold Change** | **logFC** | **absolute FC** | **Regulation** | **P-value** | **FDR** |
| 81929 | SEH1L | -2.965228604 | -1.568143333 | 2.965228604 | down | 4.64555E-15 | 4.23771E-13 |
| 5048 | PAFAH1B1 | -3.426446909 | -1.776713333 | 3.426446909 | down | 4.42608E-16 | 7.48332E-14 |
| 56648 | EIF5A2 | -2.072525712 | -1.05139 | 2.072525712 | down | 9.12489E-11 | 1.34913E-09 |
| 5500 | PPP1CB | -3.236288 | -1.69434 | 3.236288 | down | 1.1363E-17 | 5.97686E-15 |
| 25994 | HIGD1A | -2.031492446 | -1.02254 | 2.031492446 | down | 1.5025E-08 | 1.22299E-07 |
| 6924 | TCEB3 | -2.820628506 | -1.496016667 | 2.820628506 | down | 1.32326E-12 | 3.61419E-11 |
| 11193 | WBP4 | -2.172957564 | -1.11966 | 2.172957564 | down | 5.21577E-09 | 4.77342E-08 |
| 54926 | UBE2R2 | -2.213833414 | -1.146546667 | 2.213833414 | down | 2.55844E-13 | 9.37021E-12 |
| 858 | CAV2 | -4.379764607 | -2.130853333 | 4.379764607 | down | 2.51722E-20 | 1.38642E-16 |
| 85365 | ALG2 | -2.377145154 | -1.24923 | 2.377145154 | down | 6.57756E-11 | 1.01564E-09 |
| 92703 | TMEM183A | -2.446094262 | -1.29048 | 2.446094262 | down | 2.11569E-11 | 3.76192E-10 |
| 8452 | CUL3 | -7.65801242 | -2.93697 | 7.65801242 | down | 4.78542E-17 | 1.54026E-14 |
| 9768 | KIAA0101 | -3.010076181 | -1.5898 | 3.010076181 | down | 3.72028E-12 | 8.72513E-11 |
| 100526836 | BLOC1S5-TXNDC5 | -2.941658964 | -1.55663 | 2.941658964 | down | 3.02415E-17 | 1.10862E-14 |
| 6654 | SOS1 | -3.109206994 | -1.636546667 | 3.109206994 | down | 1.89931E-10 | 2.57076E-09 |
| 10137 | RBM12 | -3.568901292 | -1.83548 | 3.568901292 | down | 2.21304E-13 | 8.39522E-12 |
| 79723 | SUV39H2 | -2.234001261 | -1.15963 | 2.234001261 | down | 1.78077E-09 | 1.85773E-08 |
| 285381 | DPH3 | -2.113669778 | -1.07975 | 2.113669778 | down | 7.72018E-13 | 2.3046E-11 |
| 54585 | LZTFL1 | -2.633241792 | -1.39684 | 2.633241792 | down | 3.84607E-11 | 6.35477E-10 |
| 80820 | EEPD1 | -2.184571007 | -1.12735 | 2.184571007 | down | 8.44791E-12 | 1.73615E-10 |
| 27086 | FOXP1 | -2.043600698 | -1.031113333 | 2.043600698 | down | 1.36593E-13 | 5.69984E-12 |
| 1738 | DLD | -2.677741448 | -1.421016667 | 2.677741448 | down | 1.5483E-12 | 4.14837E-11 |
| 157922 | CAMSAP1 | -2.752800387 | -1.4609 | 2.752800387 | down | 9.40634E-14 | 4.18801E-12 |
| 8905 | AP1S2 | -2.017916764 | -1.012866667 | 2.017916764 | down | 6.48903E-14 | 3.12693E-12 |
| 57614 | KIAA1468 | -2.069257711 | -1.049113333 | 2.069257711 | down | 3.52891E-11 | 5.89122E-10 |
| 84002 | B3GNT5 | -2.494377812 | -1.31868 | 2.494377812 | down | 6.10372E-14 | 2.98181E-12 |
| 6426 | SRSF1 | -3.801055262 | -1.9264 | 3.801055262 | down | 3.28693E-15 | 3.22325E-13 |
| 79872 | CBLL1 | -3.923532997 | -1.972153333 | 3.923532997 | down | 1.34365E-14 | 9.24644E-13 |
| 3658 | IREB2 | -3.041873167 | -1.60496 | 3.041873167 | down | 3.44453E-14 | 1.91376E-12 |
| 255252 | LRRC57 | -2.290300129 | -1.195536667 | 2.290300129 | down | 3.37353E-12 | 8.07362E-11 |
| 100506548 | LOC100506548 | -2.170278218 | -1.11788 | 2.170278218 | down | 8.75207E-10 | 9.89622E-09 |
| 2562 | GABRB3 | -3.302526184 | -1.72357 | 3.302526184 | down | 1.39212E-15 | 1.67503E-13 |
| 90102 | PHLDB2 | -2.294367776 | -1.198096667 | 2.294367776 | down | 1.51001E-10 | 2.10409E-09 |
| 221895 | JAZF1 | -2.633059276 | -1.39674 | 2.633059276 | down | 2.4566E-12 | 6.16901E-11 |
| 9936 | CD302 | -2.028907839 | -1.020703333 | 2.028907839 | down | 7.34313E-08 | 5.10518E-07 |
| 100506403 | LOC100506403 | -2.461122838 | -1.299316667 | 2.461122838 | down | 4.84863E-13 | 1.5757E-11 |
| 1387 | CREBBP | -2.70400284 | -1.435096667 | 2.70400284 | down | 1.09103E-11 | 2.15703E-10 |
| 7013 | TERF1 | -3.927042832 | -1.973443333 | 3.927042832 | down | 1.74551E-13 | 6.90894E-12 |
| 653659 | TMEM183B | -2.446094262 | -1.29048 | 2.446094262 | down | 2.11569E-11 | 3.76192E-10 |
| 56288 | PARD3 | -2.967229809 | -1.569116667 | 2.967229809 | down | 7.33542E-14 | 3.45831E-12 |
| 152137 | CCDC50 | -2.782921087 | -1.4766 | 2.782921087 | down | 2.08045E-12 | 5.33371E-11 |
| 169200 | TMEM64 | -4.66296198 | -2.221246667 | 4.66296198 | down | 8.2987E-14 | 3.80276E-12 |
| 23185 | LARP4B | -2.344537465 | -1.229303333 | 2.344537465 | down | 1.29615E-11 | 2.48746E-10 |
| 119032 | BORCS7 | -2.546125149 | -1.348303333 | 2.546125149 | down | 7.24266E-13 | 2.18701E-11 |
| 8847 | DLEU2 | -2.776421617 | -1.473226667 | 2.776421617 | down | 1.89432E-11 | 3.42576E-10 |
| 6198 | RPS6KB1 | -3.13204151 | -1.647103333 | 3.13204151 | down | 6.30325E-13 | 1.94681E-11 |
| 57674 | RNF213 | -2.665858119 | -1.4146 | 2.665858119 | down | 1.5322E-14 | 1.01521E-12 |
| 10135 | NAMPT | -3.198997799 | -1.67762 | 3.198997799 | down | 1.16489E-16 | 2.98435E-14 |
| 2935 | GSPT1 | -5.916264967 | -2.564686667 | 5.916264967 | down | 9.83912E-19 | 1.54376E-15 |
| 10133 | OPTN | -2.939892359 | -1.555763333 | 2.939892359 | down | 4.04204E-14 | 2.1542E-12 |
| 5903 | RANBP2 | -2.956629223 | -1.563953333 | 2.956629223 | down | 1.65677E-13 | 6.64488E-12 |
| 387921 | NHLRC3 | -2.25983379 | -1.176216667 | 2.25983379 | down | 9.292E-11 | 1.37022E-09 |
| 90871 | TMEM261 | -2.211793453 | -1.145216667 | 2.211793453 | down | 9.06511E-09 | 7.84423E-08 |
| 10106 | CTDSP2 | -2.102243814 | -1.07193 | 2.102243814 | down | 1.05468E-14 | 7.80564E-13 |
| 6443 | SGCB | -2.480928229 | -1.31088 | 2.480928229 | down | 1.00267E-12 | 2.8792E-11 |
| 51115 | RMDN1 | -2.457060418 | -1.296933333 | 2.457060418 | down | 2.91063E-10 | 3.74694E-09 |
| 8092 | ALX1 | -2.128627244 | -1.089923333 | 2.128627244 | down | 3.93856E-11 | 6.49118E-10 |
| 56946 | EMSY | -2.186161527 | -1.1284 | 2.186161527 | down | 2.63553E-06 | 1.30446E-05 |
| 90806 | ANGEL2 | -2.42612968 | -1.278656667 | 2.42612968 | down | 1.22736E-12 | 3.41855E-11 |
| 9987 | HNRNPDL | -3.411879434 | -1.770566667 | 3.411879434 | down | 6.35632E-16 | 9.23432E-14 |
| 29110 | TBK1 | -2.085769886 | -1.06058 | 2.085769886 | down | 5.69955E-15 | 4.94099E-13 |
| 6228 | RPS23 | -2.770750234 | -1.470276667 | 2.770750234 | down | 5.94131E-17 | 1.84959E-14 |
| 80155 | NAA15 | -2.761668204 | -1.46554 | 2.761668204 | down | 1.90074E-11 | 3.4342E-10 |
| 83787 | ARMC10 | -2.490830183 | -1.316626667 | 2.490830183 | down | 1.40587E-13 | 5.83615E-12 |
| 3149 | HMGB3 | -3.729296059 | -1.898903333 | 3.729296059 | down | 9.05914E-17 | 2.43243E-14 |
| 10236 | HNRNPR | -2.145186878 | -1.101103333 | 2.145186878 | down | 2.56588E-09 | 2.56425E-08 |
| 3843 | IPO5 | -4.321852993 | -2.11165 | 4.321852993 | down | 3.75845E-15 | 3.561E-13 |
| 83716 | CRISPLD2 | -2.016099256 | -1.011566667 | 2.016099256 | down | 7.54827E-12 | 1.58672E-10 |
| 93587 | TRMT10A | -2.207994635 | -1.142736667 | 2.207994635 | down | 8.27263E-10 | 9.40017E-09 |
| 55283 | MCOLN3 | -2.207357033 | -1.14232 | 2.207357033 | down | 6.19092E-09 | 5.57481E-08 |
| 2070 | EYA4 | -2.816109307 | -1.493703333 | 2.816109307 | down | 1.39317E-14 | 9.52042E-13 |
| 55284 | UBE2W | -2.960498248 | -1.56584 | 2.960498248 | down | 4.93843E-14 | 2.52226E-12 |
| 3638 | INSIG1 | -2.422539182 | -1.27652 | 2.422539182 | down | 6.20438E-15 | 5.26613E-13 |
| 10284 | SAP18 | -2.257474983 | -1.17471 | 2.257474983 | down | 1.48907E-13 | 6.11392E-12 |
| 9448 | MAP4K4 | -4.070859228 | -2.025333333 | 4.070859228 | down | 5.0736E-16 | 8.07178E-14 |
| 4976 | OPA1 | -2.553660376 | -1.352566667 | 2.553660376 | down | 1.46119E-10 | 2.05064E-09 |
| 140688 | NOL4L | -2.00415858 | -1.002996667 | 2.00415858 | down | 5.18686E-11 | 8.25373E-10 |
| 134218 | DNAJC21 | -2.951919406 | -1.561653333 | 2.951919406 | down | 1.97011E-10 | 2.65377E-09 |
| 10592 | SMC2 | -3.691771685 | -1.884313333 | 3.691771685 | down | 6.99985E-13 | 2.12844E-11 |
| 1386 | ATF2 | -2.266160432 | -1.18025 | 2.266160432 | down | 1.29684E-12 | 3.55283E-11 |
| 11196 | SEC23IP | -2.419826034 | -1.274903333 | 2.419826034 | down | 3.11863E-15 | 3.10478E-13 |
| 10483 | SEC23B | -2.501066354 | -1.322543333 | 2.501066354 | down | 2.4376E-14 | 1.462E-12 |
| 26097 | CHTOP | -2.123317333 | -1.08632 | 2.123317333 | down | 3.25999E-14 | 1.83482E-12 |
| 55758 | RCOR3 | -2.457923476 | -1.29744 | 2.457923476 | down | 3.84899E-13 | 1.29594E-11 |
| 839 | CASP6 | -2.220251847 | -1.150723333 | 2.220251847 | down | 5.40973E-09 | 4.9417E-08 |
| 192670 | AGO4 | -2.146867767 | -1.102233333 | 2.146867767 | down | 4.17121E-10 | 5.1403E-09 |
| 55326 | AGPAT5 | -4.604740961 | -2.20312 | 4.604740961 | down | 2.94978E-14 | 1.68666E-12 |
| 734 | OSGIN2 | -2.200279297 | -1.137686667 | 2.200279297 | down | 9.02403E-12 | 1.83118E-10 |
| 54664 | TMEM106B | -2.464798996 | -1.30147 | 2.464798996 | down | 7.37291E-11 | 1.11877E-09 |
| 79699 | ZYG11B | -2.031853896 | -1.022796667 | 2.031853896 | down | 1.16174E-11 | 2.27175E-10 |
| 79074 | C2orf49 | -2.188557062 | -1.12998 | 2.188557062 | down | 1.09266E-11 | 2.15917E-10 |
| 81573 | ANKRD13C | -3.139068838 | -1.650336667 | 3.139068838 | down | 3.70987E-13 | 1.26664E-11 |
| 868 | CBLB | -2.06013184 | -1.042736667 | 2.06013184 | down | 1.44862E-08 | 1.18363E-07 |
| 51108 | METTL9 | -3.358592275 | -1.747856667 | 3.358592275 | down | 3.95425E-15 | 3.72849E-13 |
| 83660 | TLN2 | -2.061998572 | -1.044043333 | 2.061998572 | down | 2.41377E-07 | 1.49314E-06 |
| 382 | ARF6 | -3.298202549 | -1.72168 | 3.298202549 | down | 8.05335E-18 | 4.86361E-15 |
| 22800 | RRAS2 | -3.483352963 | -1.800476667 | 3.483352963 | down | 1.45068E-17 | 6.91356E-15 |
| 54708 | MARCH5 | -2.546231041 | -1.348363333 | 2.546231041 | down | 1.83482E-12 | 4.8141E-11 |
| 84295 | PHF6 | -3.050079119 | -1.608846667 | 3.050079119 | down | 7.1857E-12 | 1.52202E-10 |
| 202018 | TAPT1 | -3.619549133 | -1.85581 | 3.619549133 | down | 1.81744E-14 | 1.16485E-12 |
| 7329 | UBE2I | -3.280054986 | -1.71372 | 3.280054986 | down | 2.94352E-12 | 7.20273E-11 |
| 81689 | ISCA1 | -2.539961566 | -1.344806667 | 2.539961566 | down | 1.53355E-16 | 3.49729E-14 |
| 81608 | FIP1L1 | -2.839506078 | -1.50564 | 2.839506078 | down | 2.89234E-10 | 3.72707E-09 |
| 900 | CCNG1 | -3.746517006 | -1.90555 | 3.746517006 | down | 7.98722E-15 | 6.32927E-13 |
| 9218 | VAPA | -3.968617902 | -1.988636667 | 3.968617902 | down | 9.60911E-17 | 2.54674E-14 |
| 50862 | RNF141 | -2.536003377 | -1.342556667 | 2.536003377 | down | 9.23077E-15 | 7.03062E-13 |
| 51018 | RRP15 | -3.778621466 | -1.91786 | 3.778621466 | down | 8.14765E-15 | 6.4175E-13 |
| 586 | BCAT1 | -2.374181122 | -1.24743 | 2.374181122 | down | 7.45438E-13 | 2.2406E-11 |
| 84897 | TBRG1 | -4.422074508 | -2.144723333 | 4.422074508 | down | 7.65376E-13 | 2.29175E-11 |
| 9411 | ARHGAP29 | -2.409655103 | -1.268826667 | 2.409655103 | down | 8.189E-12 | 1.6906E-10 |
| 6009 | RHEB | -2.474893882 | -1.307366667 | 2.474893882 | down | 5.63107E-14 | 2.80659E-12 |
| 2107 | ETF1 | -2.604259719 | -1.380873333 | 2.604259719 | down | 9.18794E-18 | 5.46056E-15 |
| 83877 | TM2D2 | -2.694299069 | -1.42991 | 2.694299069 | down | 1.83195E-11 | 3.33759E-10 |
| 55632 | G2E3 | -3.357808607 | -1.74752 | 3.357808607 | down | 8.28492E-13 | 2.45524E-11 |
| 5238 | PGM3 | -2.256562389 | -1.174126667 | 2.256562389 | down | 8.11392E-11 | 1.21709E-09 |
| 55037 | PTCD3 | -2.153181597 | -1.10647 | 2.153181597 | down | 1.88631E-08 | 1.50356E-07 |
| 80331 | DNAJC5 | -2.372453811 | -1.24638 | 2.372453811 | down | 4.62974E-12 | 1.04958E-10 |
| 8887 | TAX1BP1 | -2.483859093 | -1.312583333 | 2.483859093 | down | 2.0813E-11 | 3.71086E-10 |
| 5877 | RABIF | -2.547502096 | -1.349083333 | 2.547502096 | down | 3.07581E-11 | 5.24332E-10 |
| 805 | CALM2 | -2.74430342 | -1.45644 | 2.74430342 | down | 1.16432E-17 | 5.97686E-15 |
| 102724200 | LOC102724200 | -2.465733136 | -1.302016667 | 2.465733136 | down | 6.92535E-09 | 6.16259E-08 |
| 55013 | CCDC109B | -3.018189382 | -1.593683333 | 3.018189382 | down | 2.3721E-11 | 4.15939E-10 |
| 51366 | UBR5 | -3.37612113 | -1.755366667 | 3.37612113 | down | 5.87188E-13 | 1.83818E-11 |
| 8894 | EIF2S2 | -2.603489641 | -1.380446667 | 2.603489641 | down | 1.95369E-12 | 5.0717E-11 |
| 9879 | DDX46 | -2.807092621 | -1.489076667 | 2.807092621 | down | 8.56724E-14 | 3.88947E-12 |
| 1122 | CHML | -2.784915074 | -1.477633333 | 2.784915074 | down | 3.71015E-17 | 1.28788E-14 |
| 2055 | CLN8 | -2.005381428 | -1.003876667 | 2.005381428 | down | 5.99632E-10 | 7.05476E-09 |
| 6711 | SPTBN1 | -3.497773007 | -1.806436667 | 3.497773007 | down | 5.01008E-12 | 1.1249E-10 |
| 6632 | SNRPD1 | -3.666177577 | -1.874276667 | 3.666177577 | down | 1.5308E-16 | 3.49729E-14 |
| 8649 | LAMTOR3 | -3.451571412 | -1.787253333 | 3.451571412 | down | 8.79751E-13 | 2.57416E-11 |
| 9373 | PLAA | -3.872011757 | -1.953083333 | 3.872011757 | down | 8.34479E-14 | 3.81497E-12 |
| 201965 | RWDD4 | -3.446112978 | -1.78497 | 3.446112978 | down | 2.90813E-14 | 1.67015E-12 |
| 121665 | SPPL3 | -2.562833937 | -1.35774 | 2.562833937 | down | 2.38421E-14 | 1.43657E-12 |
| 5051 | PAFAH2 | -2.476341014 | -1.30821 | 2.476341014 | down | 3.18628E-11 | 5.40813E-10 |
| 2730 | GCLM | -2.063518921 | -1.045106667 | 2.063518921 | down | 5.52587E-11 | 8.74353E-10 |
| 55117 | SLC6A15 | -2.806761866 | -1.488906667 | 2.806761866 | down | 1.58869E-10 | 2.20278E-09 |
| 64710 | NUCKS1 | -2.044838163 | -1.031986667 | 2.044838163 | down | 2.96159E-16 | 5.69453E-14 |
| 22936 | ELL2 | -5.462898684 | -2.449666667 | 5.462898684 | down | 3.2896E-17 | 1.17304E-14 |
| 93973 | ACTR8 | -2.86373236 | -1.517896667 | 2.86373236 | down | 1.23493E-11 | 2.39093E-10 |
| 3915 | LAMC1 | -2.197408866 | -1.135803333 | 2.197408866 | down | 1.09679E-15 | 1.38333E-13 |
| 4076 | CAPRIN1 | -2.098987148 | -1.069693333 | 2.098987148 | down | 1.45997E-09 | 1.55618E-08 |
| 116228 | COX20 | -2.648287958 | -1.40506 | 2.648287958 | down | 1.85222E-14 | 1.17753E-12 |
| 23041 | MON2 | -2.622664624 | -1.391033333 | 2.622664624 | down | 2.08694E-11 | 3.71753E-10 |
| 51123 | ZNF706 | -7.557574698 | -2.917923333 | 7.557574698 | down | 1.76889E-14 | 1.14119E-12 |
| 56987 | BBX | -2.688783013 | -1.426953333 | 2.688783013 | down | 1.06059E-14 | 7.83458E-13 |
| 28232 | SLCO3A1 | -2.246667564 | -1.167786667 | 2.246667564 | down | 2.90039E-08 | 2.21943E-07 |
| 26156 | RSL1D1 | -2.283130515 | -1.191013333 | 2.283130515 | down | 4.76041E-15 | 4.32239E-13 |
| 9583 | ENTPD4 | -2.602834052 | -1.380083333 | 2.602834052 | down | 1.91529E-14 | 1.2059E-12 |
| 6241 | RRM2 | -2.616031658 | -1.38738 | 2.616031658 | down | 7.57985E-16 | 1.04323E-13 |
| 5873 | RAB27A | -2.257266358 | -1.174576667 | 2.257266358 | down | 5.54312E-07 | 3.18065E-06 |
| 64327 | LMBR1 | -2.12459816 | -1.08719 | 2.12459816 | down | 2.96089E-12 | 7.2362E-11 |
| 5901 | RAN | -3.226656498 | -1.69004 | 3.226656498 | down | 3.50403E-18 | 2.92438E-15 |
| 84864 | MINA | -3.612230611 | -1.85289 | 3.612230611 | down | 1.1747E-10 | 1.68474E-09 |
| 23011 | RAB21 | -2.022420978 | -1.016083333 | 2.022420978 | down | 2.02359E-14 | 1.25992E-12 |
| 153768 | PRELID2 | -3.735703619 | -1.90138 | 3.735703619 | down | 4.58549E-13 | 1.50393E-11 |
| 144348 | ZNF664 | -2.051624446 | -1.036766667 | 2.051624446 | down | 1.33841E-10 | 1.89323E-09 |
| 338699 | ANKRD42 | -2.182300844 | -1.12585 | 2.182300844 | down | 3.21212E-11 | 5.44492E-10 |
| 10206 | TRIM13 | -3.891518818 | -1.960333333 | 3.891518818 | down | 2.02658E-11 | 3.62319E-10 |
| 91433 | RCCD1 | -2.731133811 | -1.4495 | 2.731133811 | down | 6.8926E-12 | 1.47256E-10 |
| 169981 | SPIN3 | -2.473499029 | -1.306553333 | 2.473499029 | down | 3.74352E-13 | 1.27134E-11 |
| 51312 | SLC25A37 | -3.260733799 | -1.705196667 | 3.260733799 | down | 7.0957E-15 | 5.77446E-13 |
| 6581 | SLC22A3 | -4.089213343 | -2.031823333 | 4.089213343 | down | 2.12479E-15 | 2.29299E-13 |
| 25957 | PNISR | -4.39773295 | -2.13676 | 4.39773295 | down | 7.17842E-14 | 3.40476E-12 |
| 5217 | PFN2 | -3.426272744 | -1.77664 | 3.426272744 | down | 3.63714E-16 | 6.60495E-14 |
| 51514 | DTL | -2.241638025 | -1.164553333 | 2.241638025 | down | 7.23337E-13 | 2.18589E-11 |
| 6760 | SS18 | -2.163279385 | -1.11322 | 2.163279385 | down | 2.89508E-13 | 1.03424E-11 |
| 57396 | CLK4 | -2.646721997 | -1.404206667 | 2.646721997 | down | 5.81016E-09 | 5.26579E-08 |
| 55075 | UACA | -5.063962308 | -2.340266667 | 5.063962308 | down | 8.88101E-13 | 2.59388E-11 |
| 399818 | METTL10 | -4.1180747 | -2.04197 | 4.1180747 | down | 5.77512E-14 | 2.84574E-12 |
| 6431 | SRSF6 | -2.504917925 | -1.324763333 | 2.504917925 | down | 5.15769E-16 | 8.12492E-14 |
| 9706 | ULK2 | -2.353035903 | -1.234523333 | 2.353035903 | down | 3.25762E-09 | 3.15662E-08 |
| 80209 | PROSER1 | -2.724088045 | -1.445773333 | 2.724088045 | down | 1.32549E-13 | 5.55473E-12 |
| 55388 | MCM10 | -2.841927991 | -1.50687 | 2.841927991 | down | 8.45018E-15 | 6.57655E-13 |
| 400 | ARL1 | -2.624998624 | -1.392316667 | 2.624998624 | down | 3.99905E-15 | 3.73882E-13 |
| 51099 | ABHD5 | -2.061788956 | -1.043896667 | 2.061788956 | down | 4.37997E-09 | 4.08766E-08 |
| 1915 | EEF1A1 | -4.389114784 | -2.13393 | 4.389114784 | down | 4.71651E-17 | 1.54026E-14 |
| 23244 | PDS5A | -2.170970316 | -1.11834 | 2.170970316 | down | 2.54589E-14 | 1.51529E-12 |
| 54431 | DNAJC10 | -2.824241255 | -1.497863333 | 2.824241255 | down | 2.0855E-11 | 3.71666E-10 |
| 23071 | ERP44 | -2.086010857 | -1.060746667 | 2.086010857 | down | 1.92238E-09 | 1.98697E-08 |
| 57534 | MIB1 | -2.001465388 | -1.001056667 | 2.001465388 | down | 1.90893E-09 | 1.97515E-08 |
| 113444 | SMIM12 | -2.043874576 | -1.031306667 | 2.043874576 | down | 5.29075E-11 | 8.402E-10 |
| 148867 | SLC30A7 | -2.113249829 | -1.079463333 | 2.113249829 | down | 2.27758E-12 | 5.78615E-11 |
| 10299 | MARCH6 | -2.829387941 | -1.50049 | 2.829387941 | down | 1.41978E-11 | 2.67745E-10 |
| 57542 | KLHL42 | -3.312682645 | -1.728 | 3.312682645 | down | 8.411E-12 | 1.73005E-10 |
| 10128 | LRPPRC | -5.529886999 | -2.46725 | 5.529886999 | down | 5.97777E-16 | 8.84822E-14 |
| 8614 | STC2 | -2.293779427 | -1.197726667 | 2.293779427 | down | 1.4937E-14 | 1.00154E-12 |
| 51633 | OTUD6B | -2.179912156 | -1.12427 | 2.179912156 | down | 4.3512E-10 | 5.33362E-09 |
| 9786 | KIAA0586 | -2.852888442 | -1.512423333 | 2.852888442 | down | 4.03677E-09 | 3.81134E-08 |
| 23199 | GSE1 | -2.43187094 | -1.282066667 | 2.43187094 | down | 4.61638E-14 | 2.40475E-12 |
| 54965 | PIGX | -2.335562415 | -1.22377 | 2.335562415 | down | 2.28599E-13 | 8.59711E-12 |
| 54815 | GATAD2A | -5.15888755 | -2.36706 | 5.15888755 | down | 2.93727E-17 | 1.08693E-14 |
| 57602 | USP36 | -2.195328244 | -1.134436667 | 2.195328244 | down | 1.31476E-10 | 1.86398E-09 |
| 6138 | RPL15 | -3.494792178 | -1.805206667 | 3.494792178 | down | 1.10835E-19 | 3.34422E-16 |
| 10632 | ATP5L | -2.488448736 | -1.315246667 | 2.488448736 | down | 7.6415E-15 | 6.10464E-13 |
| 5411 | PNN | -5.348409762 | -2.41911 | 5.348409762 | down | 3.37839E-18 | 2.92438E-15 |
| 5359 | PLSCR1 | -2.109011068 | -1.076566667 | 2.109011068 | down | 8.84624E-08 | 6.03258E-07 |
| 9184 | BUB3 | -2.402023058 | -1.26425 | 2.402023058 | down | 3.82522E-14 | 2.07817E-12 |
| 2137 | EXTL3 | -2.329085067 | -1.219763333 | 2.329085067 | down | 2.64568E-14 | 1.5555E-12 |
| 23272 | FAM208A | -3.520027419 | -1.815586667 | 3.520027419 | down | 3.43877E-13 | 1.19262E-11 |
| 9856 | KIAA0319 | -2.426937014 | -1.279136667 | 2.426937014 | down | 2.32439E-09 | 2.34985E-08 |
| 65084 | TMEM135 | -2.265924827 | -1.1801 | 2.265924827 | down | 1.71486E-13 | 6.80824E-12 |
| 359948 | IRF2BP2 | -4.342783403 | -2.11862 | 4.342783403 | down | 2.5593E-14 | 1.51645E-12 |
| 117178 | SSX2IP | -4.485793888 | -2.165363333 | 4.485793888 | down | 4.27834E-14 | 2.26169E-12 |
| 6210 | RPS15A | -3.731838794 | -1.899886667 | 3.731838794 | down | 3.49036E-13 | 1.20625E-11 |
| 57148 | RALGAPB | -3.380703128 | -1.757323333 | 3.380703128 | down | 1.81952E-16 | 3.98719E-14 |
| 51100 | SH3GLB1 | -3.909570165 | -1.96701 | 3.909570165 | down | 1.17374E-14 | 8.42608E-13 |
| 1810 | DR1 | -2.331820387 | -1.221456667 | 2.331820387 | down | 2.03205E-14 | 1.26319E-12 |
| 6432 | SRSF7 | -4.706920002 | -2.234783333 | 4.706920002 | down | 7.47838E-16 | 1.03563E-13 |
| 8697 | CDC23 | -2.264040867 | -1.1789 | 2.264040867 | down | 2.8407E-10 | 3.66776E-09 |
| 80025 | PANK2 | -2.203107664 | -1.13954 | 2.203107664 | down | 2.58532E-11 | 4.48911E-10 |
| 29904 | EEF2K | -2.115413948 | -1.08094 | 2.115413948 | down | 1.45861E-10 | 2.04847E-09 |
| 254427 | PROSER2 | -4.6326039 | -2.211823333 | 4.6326039 | down | 2.33504E-15 | 2.46215E-13 |
| 64343 | AZI2 | -2.350281126 | -1.232833333 | 2.350281126 | down | 2.95578E-12 | 7.2282E-11 |
| 7332 | UBE2L3 | -2.22201209 | -1.151866667 | 2.22201209 | down | 6.3194E-08 | 4.45264E-07 |
| 84901 | NFATC2IP | -2.34339475 | -1.2286 | 2.34339475 | down | 3.06812E-14 | 1.74923E-12 |
| 7468 | WHSC1 | -2.52269653 | -1.334966667 | 2.52269653 | down | 2.3937E-13 | 8.86617E-12 |
| 11030 | RBPMS | -2.43443447 | -1.283586667 | 2.43443447 | down | 5.71331E-12 | 1.25636E-10 |
| 10124 | ARL4A | -2.970337093 | -1.570626667 | 2.970337093 | down | 5.71736E-12 | 1.25638E-10 |
| 133746 | JMY | -2.100670666 | -1.07085 | 2.100670666 | down | 8.63695E-11 | 1.28425E-09 |
| 7095 | SEC62 | -3.002061086 | -1.585953333 | 3.002061086 | down | 1.85894E-12 | 4.86747E-11 |
| 8473 | OGT | -2.109059798 | -1.0766 | 2.109059798 | down | 7.63996E-12 | 1.6017E-10 |
| 51444 | RNF138 | -3.228192626 | -1.690726667 | 3.228192626 | down | 3.39273E-12 | 8.10473E-11 |
| 3224 | HOXC8 | -2.448729362 | -1.292033333 | 2.448729362 | down | 4.74345E-13 | 1.54922E-11 |
| 56889 | TM9SF3 | -3.46423468 | -1.792536667 | 3.46423468 | down | 2.84878E-16 | 5.55937E-14 |
| 10052 | GJC1 | -2.391136793 | -1.257696667 | 2.391136793 | down | 1.43016E-13 | 5.89883E-12 |
| 26984 | SEC22A | -2.902876778 | -1.537483333 | 2.902876778 | down | 4.87281E-12 | 1.09848E-10 |
| 79469 | DLEU2L | -2.776421617 | -1.473226667 | 2.776421617 | down | 1.89432E-11 | 3.42576E-10 |
| 961 | CD47 | -6.756112415 | -2.756193333 | 6.756112415 | down | 5.25735E-18 | 3.96576E-15 |
| 9616 | RNF7 | -2.917130646 | -1.54455 | 2.917130646 | down | 7.67757E-11 | 1.15962E-09 |
| 85465 | EPT1 | -2.130782494 | -1.091383333 | 2.130782494 | down | 2.04374E-15 | 2.22065E-13 |
| 5412 | UBL3 | -4.154342032 | -2.05462 | 4.154342032 | down | 4.39546E-16 | 7.48332E-14 |
| 81579 | PLA2G12A | -2.220251847 | -1.150723333 | 2.220251847 | down | 5.40973E-09 | 4.9417E-08 |
| 54800 | KLHL24 | -2.401856568 | -1.26415 | 2.401856568 | down | 9.25889E-10 | 1.04153E-08 |
| 6936 | GCFC2 | -2.493300319 | -1.318056667 | 2.493300319 | down | 5.91376E-12 | 1.29374E-10 |
| 4651 | MYO10 | -2.114412218 | -1.080256667 | 2.114412218 | down | 7.39917E-10 | 8.51871E-09 |
| 129642 | MBOAT2 | -2.725617912 | -1.446583333 | 2.725617912 | down | 4.78272E-12 | 1.08004E-10 |
| 10762 | NUP50 | -2.135099543 | -1.094303333 | 2.135099543 | down | 2.10735E-14 | 1.2936E-12 |
| 8125 | ANP32A | -2.625975276 | -1.392853333 | 2.625975276 | down | 3.10073E-15 | 3.09481E-13 |
| 6452 | SH3BP2 | -2.13557811 | -1.094626667 | 2.13557811 | down | 2.50691E-09 | 2.51235E-08 |
| 8417 | STX7 | -3.028919704 | -1.598803333 | 3.028919704 | down | 2.58952E-13 | 9.46295E-12 |
| 7073 | TIAL1 | -3.719254144 | -1.895013333 | 3.719254144 | down | 2.3807E-15 | 2.48359E-13 |
| 3281 | HSBP1 | -2.206276082 | -1.141613333 | 2.206276082 | down | 1.34141E-14 | 9.24644E-13 |
| 10204 | NUTF2 | -2.106303503 | -1.074713333 | 2.106303503 | down | 3.08491E-12 | 7.49724E-11 |
| 1854 | DUT | -2.291660504 | -1.196393333 | 2.291660504 | down | 6.05846E-13 | 1.88606E-11 |
| 2590 | GALNT2 | -2.899773065 | -1.53594 | 2.899773065 | down | 1.08072E-15 | 1.36746E-13 |
| 55319 | TMA16 | -2.495432707 | -1.31929 | 2.495432707 | down | 8.48657E-12 | 1.7417E-10 |
| 10151 | HNRNPA3P1 | -2.226364794 | -1.15469 | 2.226364794 | down | 1.18502E-16 | 2.98435E-14 |
| 29028 | ATAD2 | -3.596266662 | -1.8465 | 3.596266662 | down | 2.85639E-13 | 1.02697E-11 |
| 29979 | UBQLN1 | -2.268350113 | -1.181643333 | 2.268350113 | down | 3.66631E-17 | 1.28403E-14 |
| 51340 | CRNKL1 | -2.645713177 | -1.403656667 | 2.645713177 | down | 1.1919E-12 | 3.33946E-11 |
| 10138 | YAF2 | -2.253170779 | -1.171956667 | 2.253170779 | down | 5.00914E-11 | 7.99758E-10 |
| 219333 | USP12 | -2.461816677 | -1.299723333 | 2.461816677 | down | 5.21528E-14 | 2.64301E-12 |
| 4594 | MUT | -2.346916749 | -1.230766667 | 2.346916749 | down | 2.0855E-09 | 2.13531E-08 |
| 3141 | HLCS | -2.154032474 | -1.10704 | 2.154032474 | down | 4.15157E-11 | 6.79525E-10 |
| 6161 | RPL32 | -2.220718714 | -1.151026667 | 2.220718714 | down | 6.11766E-13 | 1.90147E-11 |
| 112487 | DTD2 | -2.001525505 | -1.0011 | 2.001525505 | down | 2.67661E-13 | 9.73379E-12 |
| 1999 | ELF3 | -3.438755797 | -1.781886667 | 3.438755797 | down | 1.43463E-13 | 5.90486E-12 |
| 2202 | EFEMP1 | -2.31231295 | -1.209336667 | 2.31231295 | down | 1.80292E-12 | 4.74948E-11 |
| 196074 | METTL15 | -3.327610713 | -1.734486667 | 3.327610713 | down | 8.63912E-14 | 3.91304E-12 |
| 10638 | SPHAR | -2.061231674 | -1.043506667 | 2.061231674 | down | 1.38954E-10 | 1.9599E-09 |
| 1488 | CTBP2 | -2.270400271 | -1.182946667 | 2.270400271 | down | 3.46312E-12 | 8.22781E-11 |
| 1075 | CTSC | -3.426264828 | -1.776636667 | 3.426264828 | down | 9.12753E-13 | 2.64813E-11 |
| 79811 | SLTM | -2.445399204 | -1.29007 | 2.445399204 | down | 3.95267E-16 | 7.01519E-14 |
| 159090 | FAM122B | -3.127774853 | -1.645136667 | 3.127774853 | down | 2.64168E-14 | 1.5555E-12 |
| 64434 | NOM1 | -2.014423009 | -1.010366667 | 2.014423009 | down | 1.25559E-09 | 1.36541E-08 |
| 490 | ATP2B1 | -2.732844426 | -1.450403333 | 2.732844426 | down | 7.4807E-12 | 1.57928E-10 |
| 9774 | BCLAF1 | -2.907582243 | -1.53982 | 2.907582243 | down | 5.71919E-19 | 9.75371E-16 |
| 22822 | PHLDA1 | -3.466212252 | -1.79336 | 3.466212252 | down | 1.30847E-15 | 1.59328E-13 |
| 55706 | NDC1 | -2.743580677 | -1.45606 | 2.743580677 | down | 5.22757E-16 | 8.20206E-14 |
| 57563 | KLHL8 | -2.142838814 | -1.099523333 | 2.142838814 | down | 3.83967E-10 | 4.79195E-09 |
| 23433 | RHOQ | -2.969445045 | -1.570193333 | 2.969445045 | down | 7.66889E-14 | 3.57854E-12 |
| 9306 | SOCS6 | -3.367061331 | -1.75149 | 3.367061331 | down | 7.49824E-16 | 1.03563E-13 |
| 84668 | FAM126A | -2.404121818 | -1.26551 | 2.404121818 | down | 6.99184E-14 | 3.32834E-12 |
| 1054 | CEBPG | -2.906359835 | -1.539213333 | 2.906359835 | down | 3.22522E-17 | 1.16784E-14 |
| 196394 | AMN1 | -2.264192573 | -1.178996667 | 2.264192573 | down | 1.30003E-10 | 1.84559E-09 |
| 219771 | CCNY | -2.153226372 | -1.1065 | 2.153226372 | down | 1.13687E-13 | 4.93293E-12 |
| 58528 | RRAGD | -5.787793034 | -2.533013333 | 5.787793034 | down | 5.91279E-16 | 8.7852E-14 |
| 5813 | PURA | -2.144562458 | -1.100683333 | 2.144562458 | down | 4.79981E-13 | 1.56149E-11 |
| 10552 | ARPC1A | -2.150049686 | -1.10437 | 2.150049686 | down | 2.48818E-14 | 1.48327E-12 |
| 79269 | DCAF10 | -2.719038665 | -1.443096667 | 2.719038665 | down | 3.56127E-14 | 1.96195E-12 |
| 6428 | SRSF3 | -2.375168722 | -1.24803 | 2.375168722 | down | 1.37626E-09 | 1.48103E-08 |
| 54602 | NDFIP2 | -2.94832044 | -1.559893333 | 2.94832044 | down | 1.86432E-11 | 3.38399E-10 |
| 84749 | USP30 | -2.652850422 | -1.407543333 | 2.652850422 | down | 1.37452E-09 | 1.48039E-08 |
| 25896 | INTS7 | -5.737252447 | -2.52036 | 5.737252447 | down | 1.9358E-14 | 1.2149E-12 |
| 129450 | TYW5 | -2.299393428 | -1.201253333 | 2.299393428 | down | 1.08827E-09 | 1.20279E-08 |
| 10772 | SRSF10 | -3.216221274 | -1.685366667 | 3.216221274 | down | 5.75533E-13 | 1.81182E-11 |
| 2734 | GLG1 | -2.304179857 | -1.204253333 | 2.304179857 | down | 8.55315E-13 | 2.52064E-11 |
| 55450 | CAMK2N1 | -2.012334309 | -1.00887 | 2.012334309 | down | 6.72206E-15 | 5.56271E-13 |
| 57634 | EP400 | -2.35350894 | -1.234813333 | 2.35350894 | down | 2.92858E-14 | 1.67944E-12 |
| 7326 | UBE2G1 | -2.253238457 | -1.172 | 2.253238457 | down | 2.48532E-15 | 2.56544E-13 |
| 51592 | TRIM33 | -2.185570627 | -1.12801 | 2.185570627 | down | 5.88045E-13 | 1.8394E-11 |
| 80306 | MED28 | -2.401684541 | -1.264046667 | 2.401684541 | down | 1.06219E-11 | 2.1084E-10 |
| 10492 | SYNCRIP | -7.672074164 | -2.939616667 | 7.672074164 | down | 7.0098E-16 | 9.8723E-14 |
| 63027 | SLC22A23 | -2.18808179 | -1.129666667 | 2.18808179 | down | 4.97997E-09 | 4.58112E-08 |
| 84869 | CBR4 | -3.650506545 | -1.868096667 | 3.650506545 | down | 1.74183E-15 | 1.95872E-13 |
| 7490 | WT1 | -2.279198641 | -1.188526667 | 2.279198641 | down | 1.63266E-11 | 3.02081E-10 |
| 57589 | RIC1 | -2.526768222 | -1.337293333 | 2.526768222 | down | 1.66707E-11 | 3.07432E-10 |
| 7410 | VAV2 | -2.348994497 | -1.232043333 | 2.348994497 | down | 2.29951E-11 | 4.04296E-10 |
| 87178 | PNPT1 | -3.602379068 | -1.84895 | 3.602379068 | down | 6.47301E-14 | 3.12305E-12 |
| 57187 | THOC2 | -2.596365216 | -1.376493333 | 2.596365216 | down | 1.88847E-11 | 3.41676E-10 |
| 102659353 | THRIL | -4.027208035 | -2.00978 | 4.027208035 | down | 4.35285E-13 | 1.44207E-11 |
| 9653 | HS2ST1 | -3.135698102 | -1.648786667 | 3.135698102 | down | 1.18055E-13 | 5.07753E-12 |
| 79145 | CHCHD7 | -2.110892828 | -1.077853333 | 2.110892828 | down | 1.47992E-11 | 2.77484E-10 |
| 8396 | PIP4K2B | -2.47770312 | -1.309003333 | 2.47770312 | down | 3.616E-13 | 1.24419E-11 |
| 375056 | MIA3 | -4.072016291 | -2.025743333 | 4.072016291 | down | 4.78004E-14 | 2.46068E-12 |
| 11098 | PRSS23 | -2.055576884 | -1.039543333 | 2.055576884 | down | 1.35354E-16 | 3.21774E-14 |
| 81567 | TXNDC5 | -2.941658964 | -1.55663 | 2.941658964 | down | 3.02415E-17 | 1.10862E-14 |
| 64854 | USP46 | -2.592624614 | -1.374413333 | 2.592624614 | down | 2.35853E-13 | 8.76905E-12 |
| 2308 | FOXO1 | -2.137849064 | -1.09616 | 2.137849064 | down | 3.47577E-11 | 5.81395E-10 |
| 8767 | RIPK2 | -2.363961708 | -1.241206667 | 2.363961708 | down | 4.35163E-11 | 7.06217E-10 |
| 5701 | PSMC2 | -3.099602759 | -1.632083333 | 3.099602759 | down | 1.75279E-14 | 1.13542E-12 |
| 23658 | LSM5 | -7.919631665 | -2.985433333 | 7.919631665 | down | 4.68633E-17 | 1.54026E-14 |
| 2257 | FGF12 | -2.919139859 | -1.545543333 | 2.919139859 | down | 1.73045E-10 | 2.37E-09 |
| 2355 | FOSL2 | -3.333782536 | -1.73716 | 3.333782536 | down | 7.43236E-16 | 1.03381E-13 |
| 4756 | NEO1 | -3.369972145 | -1.752736667 | 3.369972145 | down | 4.96207E-15 | 4.47442E-13 |
| 94239 | H2AFV | -2.282307739 | -1.190493333 | 2.282307739 | down | 3.83103E-14 | 2.07845E-12 |
| 10640 | EXOC5 | -2.801468621 | -1.486183333 | 2.801468621 | down | 3.34288E-14 | 1.86787E-12 |
| 113419 | TEX261 | -2.116787822 | -1.081876667 | 2.116787822 | down | 4.15912E-13 | 1.3849E-11 |
| 101930123 | LOC101930123 | -2.115413948 | -1.08094 | 2.115413948 | down | 1.45861E-10 | 2.04847E-09 |
| 83935 | TMEM133 | -2.735333358 | -1.451716667 | 2.735333358 | down | 1.27633E-12 | 3.51575E-11 |
| 11051 | NUDT21 | -3.400263773 | -1.765646667 | 3.400263773 | down | 4.29603E-15 | 3.98373E-13 |
| 54841 | BIVM | -2.074053823 | -1.052453333 | 2.074053823 | down | 1.44621E-10 | 2.0335E-09 |
| 1385 | CREB1 | -2.549745641 | -1.350353333 | 2.549745641 | down | 1.64372E-11 | 3.03841E-10 |
| 22852 | ANKRD26 | -2.196469805 | -1.135186667 | 2.196469805 | down | 3.05568E-09 | 2.98009E-08 |
| 121536 | AEBP2 | -4.528416534 | -2.179006667 | 4.528416534 | down | 7.07861E-14 | 3.36556E-12 |
| 51012 | PRELID3B | -2.069879334 | -1.049546667 | 2.069879334 | down | 7.42916E-14 | 3.48993E-12 |
| 56888 | KCMF1 | -5.062546778 | -2.339863333 | 5.062546778 | down | 7.94579E-12 | 1.65344E-10 |
| 10668 | CGRRF1 | -2.192945564 | -1.13287 | 2.192945564 | down | 6.36214E-10 | 7.44495E-09 |
| 84955 | NUDCD1 | -3.909019189 | -1.966806667 | 3.909019189 | down | 4.93403E-14 | 2.52226E-12 |
| 84188 | FAR1 | -4.40743716 | -2.13994 | 4.40743716 | down | 2.01265E-16 | 4.33179E-14 |
| 441027 | TMEM150C | -2.380167878 | -1.251063333 | 2.380167878 | down | 3.29901E-09 | 3.19043E-08 |
| 85476 | GFM1 | -2.130639728 | -1.091286667 | 2.130639728 | down | 9.30303E-16 | 1.22786E-13 |
| 85015 | USP45 | -2.071501215 | -1.050676667 | 2.071501215 | down | 1.80805E-07 | 1.14889E-06 |
| 5862 | RAB2A | -3.072472292 | -1.6194 | 3.072472292 | down | 1.30706E-14 | 9.12266E-13 |
| 83636 | C19orf12 | -2.165935078 | -1.11499 | 2.165935078 | down | 1.08584E-09 | 1.20079E-08 |
| 4090 | SMAD5 | -2.838036872 | -1.504893333 | 2.838036872 | down | 3.30321E-10 | 4.19179E-09 |
| 9295 | SRSF11 | -3.195370751 | -1.675983333 | 3.195370751 | down | 4.65941E-14 | 2.41387E-12 |
| 57213 | SPRYD7 | -3.911060898 | -1.96756 | 3.911060898 | down | 1.71381E-14 | 1.11668E-12 |
| 221477 | C6orf89 | -2.625593065 | -1.392643333 | 2.625593065 | down | 3.77551E-14 | 2.05973E-12 |
| 8573 | CASK | -3.19406425 | -1.675393333 | 3.19406425 | down | 3.64015E-13 | 1.25031E-11 |
| 203197 | C9orf91 | -2.025572888 | -1.01833 | 2.025572888 | down | 3.41344E-10 | 4.31772E-09 |
| 89890 | KBTBD6 | -2.359274579 | -1.238343333 | 2.359274579 | down | 1.93953E-10 | 2.61887E-09 |
| 7528 | YY1 | -2.286804974 | -1.193333333 | 2.286804974 | down | 1.7097E-12 | 4.53435E-11 |
| 84172 | POLR1B | -2.024674515 | -1.01769 | 2.024674515 | down | 2.27867E-10 | 3.01555E-09 |
| 492311 | IGIP | -2.453645231 | -1.294926667 | 2.453645231 | down | 2.12679E-09 | 2.17135E-08 |
| 8239 | USP9X | -2.710645909 | -1.438636667 | 2.710645909 | down | 4.13493E-11 | 6.77213E-10 |
| 10424 | PGRMC2 | -4.630260407 | -2.211093333 | 4.630260407 | down | 1.1108E-18 | 1.65228E-15 |
| 25948 | KBTBD2 | -2.128735446 | -1.089996667 | 2.128735446 | down | 5.10925E-12 | 1.14259E-10 |
| 55714 | TENM3 | -3.507152101 | -1.8103 | 3.507152101 | down | 1.53138E-14 | 1.01521E-12 |
| 7163 | TPD52 | -3.263575315 | -1.706453333 | 3.263575315 | down | 4.16492E-13 | 1.38566E-11 |
| 4300 | MLLT3 | -3.959468181 | -1.985306667 | 3.959468181 | down | 2.89475E-13 | 1.03424E-11 |
| 26091 | HERC4 | -2.183375094 | -1.12656 | 2.183375094 | down | 1.20234E-09 | 1.3148E-08 |
| 54464 | XRN1 | -3.293557339 | -1.719646667 | 3.293557339 | down | 4.39646E-15 | 4.05768E-13 |
| 9716 | AQR | -2.679641499 | -1.42204 | 2.679641499 | down | 8.51136E-11 | 1.26991E-09 |
| 3589 | IL11 | -2.493098701 | -1.31794 | 2.493098701 | down | 6.40656E-12 | 1.38609E-10 |
| 54887 | UHRF1BP1 | -2.15460489 | -1.107423333 | 2.15460489 | down | 2.68005E-13 | 9.73379E-12 |
| 6168 | RPL37A | -2.170614209 | -1.118103333 | 2.170614209 | down | 1.08182E-12 | 3.06166E-11 |
| 10152 | ABI2 | -3.207612799 | -1.6815 | 3.207612799 | down | 6.04661E-13 | 1.88536E-11 |
| 25963 | TMEM87A | -2.451282344 | -1.293536667 | 2.451282344 | down | 2.56202E-07 | 1.57372E-06 |
| 100526664 | LY75-CD302 | -2.028907839 | -1.020703333 | 2.028907839 | down | 7.34313E-08 | 5.10518E-07 |
| 27032 | ATP2C1 | -3.448335151 | -1.7859 | 3.448335151 | down | 8.99867E-13 | 2.61948E-11 |
| 200933 | FBXO45 | -2.269928204 | -1.182646667 | 2.269928204 | down | 8.80037E-13 | 2.57416E-11 |
| 134553 | C5orf24 | -2.203856059 | -1.14003 | 2.203856059 | down | 6.05218E-16 | 8.8718E-14 |
| 258010 | SVIP | -3.69134522 | -1.884146667 | 3.69134522 | down | 7.32811E-15 | 5.93895E-13 |
| 253943 | YTHDF3 | -2.460457619 | -1.298926667 | 2.460457619 | down | 7.74547E-16 | 1.05761E-13 |
| 3183 | HNRNPC | -3.163255305 | -1.66141 | 3.163255305 | down | 1.42735E-14 | 9.66574E-13 |
| 4233 | MET | -2.10540824 | -1.0741 | 2.10540824 | down | 7.07554E-15 | 5.77003E-13 |
| 22931 | RAB18 | -4.134501329 | -2.047713333 | 4.134501329 | down | 7.02198E-16 | 9.8723E-14 |
| 4053 | LTBP2 | -2.598633782 | -1.377753333 | 2.598633782 | down | 1.42262E-12 | 3.84843E-11 |
| 7707 | ZNF148 | -2.388800987 | -1.256286667 | 2.388800987 | down | 4.69785E-11 | 7.55528E-10 |
| 79137 | FAM134A | -2.07080255 | -1.05019 | 2.07080255 | down | 2.54924E-08 | 1.97033E-07 |
| 23142 | DCUN1D4 | -2.163134441 | -1.113123333 | 2.163134441 | down | 8.32648E-12 | 1.71537E-10 |
| 23111 | SPG20 | -3.531701309 | -1.820363333 | 3.531701309 | down | 5.81382E-11 | 9.14759E-10 |
| 80018 | NAA25 | -4.148606052 | -2.052626667 | 4.148606052 | down | 1.09953E-14 | 8.07662E-13 |
| 64718 | UNKL | -2.321187932 | -1.214863333 | 2.321187932 | down | 3.71803E-13 | 1.26664E-11 |
| 9931 | HELZ | -2.984998073 | -1.57773 | 2.984998073 | down | 1.59993E-11 | 2.96584E-10 |
| 144108 | SPTY2D1 | -2.461191076 | -1.299356667 | 2.461191076 | down | 7.7785E-12 | 1.6238E-10 |
| 58517 | RBM25 | -2.002385848 | -1.00172 | 2.002385848 | down | 6.94212E-09 | 6.17611E-08 |
| 5515 | PPP2CA | -3.947784759 | -1.981043333 | 3.947784759 | down | 2.01099E-12 | 5.19297E-11 |
| 9922 | IQSEC1 | -2.184061276 | -1.127013333 | 2.184061276 | down | 1.35243E-07 | 8.84002E-07 |
| 65056 | GPBP1 | -2.262696888 | -1.178043333 | 2.262696888 | down | 3.87496E-11 | 6.3971E-10 |
| 6444 | SGCD | -2.273376554 | -1.184836667 | 2.273376554 | down | 1.38125E-10 | 1.9496E-09 |
| 51582 | AZIN1 | -2.790318872 | -1.48043 | 2.790318872 | down | 8.6557E-17 | 2.39099E-14 |
| 2770 | GNAI1 | -4.351139687 | -2.121393333 | 4.351139687 | down | 2.31718E-16 | 4.83466E-14 |
| 55529 | TMEM55A | -2.558195759 | -1.355126667 | 2.558195759 | down | 9.68548E-08 | 6.53782E-07 |
| 7915 | ALDH5A1 | -2.332612506 | -1.221946667 | 2.332612506 | down | 1.29225E-09 | 1.4014E-08 |
| 51182 | HSPA14 | -2.113210768 | -1.079436667 | 2.113210768 | down | 1.41033E-11 | 2.66732E-10 |
| 7042 | TGFB2 | -2.355418368 | -1.235983333 | 2.355418368 | down | 3.46131E-10 | 4.367E-09 |
| 10254 | STAM2 | -4.29478725 | -2.102586667 | 4.29478725 | down | 6.75435E-13 | 2.06984E-11 |
| 149371 | EXOC8 | -2.135232742 | -1.094393333 | 2.135232742 | down | 5.65066E-12 | 1.24661E-10 |
| 79882 | ZC3H14 | -3.131701411 | -1.646946667 | 3.131701411 | down | 1.36295E-15 | 1.65005E-13 |
| 50515 | CHST11 | -3.324997679 | -1.733353333 | 3.324997679 | down | 5.6345E-16 | 8.55257E-14 |
| 993 | CDC25A | -2.526289545 | -1.33702 | 2.526289545 | down | 1.63981E-09 | 1.72259E-08 |
| 85403 | EAF1 | -2.009662634 | -1.006953333 | 2.009662634 | down | 1.34214E-11 | 2.55809E-10 |
| 55664 | CDC37L1 | -2.802666339 | -1.4868 | 2.802666339 | down | 4.1177E-13 | 1.37344E-11 |
| 54556 | ING3 | -2.784413227 | -1.477373333 | 2.784413227 | down | 6.24533E-10 | 7.31918E-09 |
| 11043 | MID2 | -2.522888884 | -1.335076667 | 2.522888884 | down | 3.32919E-10 | 4.22067E-09 |
| 66036 | MTMR9 | -2.494268313 | -1.318616667 | 2.494268313 | down | 6.3004E-13 | 1.94681E-11 |
| 55110 | MAGOHB | -2.491348191 | -1.316926667 | 2.491348191 | down | 2.8408E-09 | 2.79624E-08 |
| 84959 | UBASH3B | -3.866031341 | -1.950853333 | 3.866031341 | down | 1.5689E-17 | 7.20414E-15 |
| 84294 | UTP23 | -2.824038976 | -1.49776 | 2.824038976 | down | 1.52622E-14 | 1.01468E-12 |
| 1719 | DHFR | -2.053426535 | -1.038033333 | 2.053426535 | down | 1.05664E-12 | 3.00337E-11 |
| 57531 | HACE1 | -2.119925159 | -1.084013333 | 2.119925159 | down | 3.93068E-10 | 4.88689E-09 |
| 57456 | KIAA1143 | -4.425150943 | -2.145726667 | 4.425150943 | down | 1.45885E-17 | 6.91356E-15 |
| 9527 | GOSR1 | -2.350384304 | -1.232896667 | 2.350384304 | down | 1.32313E-13 | 5.55076E-12 |
| 57670 | KIAA1549 | -2.508844985 | -1.327023333 | 2.508844985 | down | 1.31835E-09 | 1.42654E-08 |
| 401152 | C4orf3 | -2.127791319 | -1.089356667 | 2.127791319 | down | 1.78045E-10 | 2.42915E-09 |
| 10015 | PDCD6IP | -6.593677534 | -2.721083333 | 6.593677534 | down | 5.58637E-16 | 8.55257E-14 |
| 10988 | METAP2 | -4.118455308 | -2.042103333 | 4.118455308 | down | 5.27477E-15 | 4.69168E-13 |
| 89796 | NAV1 | -2.215353103 | -1.147536667 | 2.215353103 | down | 9.73833E-08 | 6.56898E-07 |
| 51105 | PHF20L1 | -4.765794244 | -2.252716667 | 4.765794244 | down | 5.43171E-15 | 4.78784E-13 |
| 28982 | FLVCR1 | -2.174127678 | -1.120436667 | 2.174127678 | down | 5.2103E-08 | 3.7438E-07 |
| 220988 | HNRNPA3 | -3.857724188 | -1.94775 | 3.857724188 | down | 1.3487E-14 | 9.2649E-13 |
| 7803 | PTP4A1 | -2.215133016 | -1.147393333 | 2.215133016 | down | 1.15075E-17 | 5.97686E-15 |
| 4646 | MYO6 | -3.966967744 | -1.988036667 | 3.966967744 | down | 4.22699E-12 | 9.74171E-11 |
| 29761 | USP25 | -3.035056415 | -1.601723333 | 3.035056415 | down | 1.72824E-12 | 4.57734E-11 |
| 3551 | IKBKB | -2.400486241 | -1.263326667 | 2.400486241 | down | 9.84632E-15 | 7.37065E-13 |
| 64224 | HERPUD2 | -2.009774076 | -1.007033333 | 2.009774076 | down | 2.9934E-10 | 3.8409E-09 |
| 115294 | PCMTD1 | -2.420838214 | -1.275506667 | 2.420838214 | down | 1.64566E-13 | 6.6206E-12 |
| 203547 | VMA21 | -2.543473389 | -1.3468 | 2.543473389 | down | 3.6096E-16 | 6.60495E-14 |
| 518 | ATP5G3 | -4.640756495 | -2.21436 | 4.640756495 | down | 3.72139E-12 | 8.72513E-11 |
| 57132 | CHMP1B | -2.194172067 | -1.133676667 | 2.194172067 | down | 1.40654E-12 | 3.8102E-11 |
| 26512 | INTS6 | -3.179816103 | -1.668943333 | 3.179816103 | down | 7.12673E-11 | 1.08646E-09 |
| 9584 | RBM39 | -2.254894601 | -1.17306 | 2.254894601 | down | 1.97503E-16 | 4.28014E-14 |
| 3667 | IRS1 | -2.090642912 | -1.063946667 | 2.090642912 | down | 5.1099E-13 | 1.63889E-11 |
| 286343 | LURAP1L | -4.30606471 | -2.10637 | 4.30606471 | down | 1.8654E-12 | 4.87477E-11 |
| 2673 | GFPT1 | -6.743885304 | -2.75358 | 6.743885304 | down | 2.36904E-16 | 4.89082E-14 |
| 256356 | GK5 | -2.507865541 | -1.32646 | 2.507865541 | down | 1.53134E-09 | 1.62036E-08 |
| 137492 | VPS37A | -2.183501214 | -1.126643333 | 2.183501214 | down | 1.46406E-09 | 1.55969E-08 |
| 23243 | ANKRD28 | -2.135499164 | -1.094573333 | 2.135499164 | down | 7.00018E-11 | 1.07008E-09 |
| 23141 | ANKLE2 | -2.036902136 | -1.026376667 | 2.036902136 | down | 2.67639E-13 | 9.73379E-12 |
| 5208 | PFKFB2 | -2.631763775 | -1.39603 | 2.631763775 | down | 3.70896E-13 | 1.26664E-11 |
| 23450 | SF3B3 | -2.905829389 | -1.53895 | 2.905829389 | down | 5.85905E-13 | 1.83576E-11 |
| 23788 | MTCH2 | -2.475368539 | -1.307643333 | 2.475368539 | down | 1.13288E-15 | 1.41972E-13 |
| 10026 | PIGK | -2.399183214 | -1.262543333 | 2.399183214 | down | 6.99525E-13 | 2.12844E-11 |
| 79664 | ICE2 | -2.208377284 | -1.142986667 | 2.208377284 | down | 5.42167E-10 | 6.47382E-09 |
| 55161 | TMEM33 | -3.276495007 | -1.712153333 | 3.276495007 | down | 2.91232E-13 | 1.03945E-11 |
| 56943 | ENY2 | -2.78678171 | -1.4786 | 2.78678171 | down | 7.42365E-15 | 5.97932E-13 |
| 154007 | SNRNP48 | -2.177385215 | -1.122596667 | 2.177385215 | down | 3.23171E-10 | 4.11303E-09 |
| 7874 | USP7 | -2.454864395 | -1.295643333 | 2.454864395 | down | 2.85124E-10 | 3.68015E-09 |
| 3192 | HNRNPU | -4.338120117 | -2.11707 | 4.338120117 | down | 1.14102E-15 | 1.42537E-13 |
| 22929 | SEPHS1 | -2.188051457 | -1.129646667 | 2.188051457 | down | 5.73414E-13 | 1.8066E-11 |
| 56937 | PMEPA1 | -2.497191857 | -1.320306667 | 2.497191857 | down | 2.00564E-18 | 2.07029E-15 |
| 79892 | MCMBP | -2.960347767 | -1.565766667 | 2.960347767 | down | 4.72743E-18 | 3.63595E-15 |
| 55766 | H2AFJ | -2.582843088 | -1.36896 | 2.582843088 | down | 2.17727E-09 | 2.21917E-08 |
| 801 | CALM1 | -2.74430342 | -1.45644 | 2.74430342 | down | 1.16432E-17 | 5.97686E-15 |
| 483 | ATP1B3 | -2.138852014 | -1.096836667 | 2.138852014 | down | 4.92404E-14 | 2.52148E-12 |
| 154807 | VKORC1L1 | -2.843898548 | -1.50787 | 2.843898548 | down | 2.33249E-14 | 1.40974E-12 |
| 91860 | CALML4 | -2.01141857 | -1.008213333 | 2.01141857 | down | 7.16381E-10 | 8.27445E-09 |
| 95681 | CEP41 | -2.180546869 | -1.12469 | 2.180546869 | down | 7.25674E-13 | 2.18958E-11 |
| 2531 | KDSR | -3.852878434 | -1.945936667 | 3.852878434 | down | 3.10706E-14 | 1.76886E-12 |
| 9043 | SPAG9 | -6.464834033 | -2.692613333 | 6.464834033 | down | 1.57486E-18 | 1.76497E-15 |
| 7320 | UBE2B | -2.387520852 | -1.255513333 | 2.387520852 | down | 1.02604E-14 | 7.63691E-13 |
| 1977 | EIF4E | -6.272352617 | -2.649006667 | 6.272352617 | down | 1.99987E-13 | 7.70578E-12 |
| 154881 | KCTD7 | -3.256795952 | -1.703453333 | 3.256795952 | down | 2.48804E-16 | 5.10959E-14 |
| 5774 | PTPN3 | -2.069707174 | -1.049426667 | 2.069707174 | down | 3.73853E-10 | 4.68063E-09 |
| 221294 | NT5DC1 | -4.405512928 | -2.13931 | 4.405512928 | down | 7.80724E-14 | 3.61985E-12 |
| 55069 | TMEM248 | -2.475402855 | -1.307663333 | 2.475402855 | down | 1.47138E-15 | 1.73841E-13 |
| 2618 | GART | -2.009017318 | -1.00649 | 2.009017318 | down | 3.107E-11 | 5.29189E-10 |
| 79745 | CLIP4 | -2.090725031 | -1.064003333 | 2.090725031 | down | 3.82645E-13 | 1.2905E-11 |
| 92 | ACVR2A | -2.052511066 | -1.03739 | 2.052511066 | down | 1.37269E-08 | 1.12785E-07 |
| 642443 | ADH5P4 | -2.724547544 | -1.446016667 | 2.724547544 | down | 1.11114E-14 | 8.12422E-13 |
| 57539 | WDR35 | -3.050840309 | -1.609206667 | 3.050840309 | down | 1.61764E-14 | 1.0593E-12 |
| 58487 | CREBZF | -2.10057845 | -1.070786667 | 2.10057845 | down | 1.20189E-11 | 2.33502E-10 |
| 5867 | RAB4A | -2.061231674 | -1.043506667 | 2.061231674 | down | 1.38954E-10 | 1.9599E-09 |
| 23161 | SNX13 | -3.34389605 | -1.74153 | 3.34389605 | down | 8.39675E-15 | 6.54796E-13 |
| 1111 | CHEK1 | -2.110224757 | -1.077396667 | 2.110224757 | down | 8.04261E-13 | 2.39538E-11 |
| 9969 | MED13 | -2.459553892 | -1.298396667 | 2.459553892 | down | 9.83994E-15 | 7.37065E-13 |
| 501 | ALDH7A1 | -2.657733903 | -1.410196667 | 2.657733903 | down | 2.91695E-11 | 5.00952E-10 |
| 54107 | POLE3 | -2.009722997 | -1.006996667 | 2.009722997 | down | 3.35718E-15 | 3.25828E-13 |
| 55276 | PGM2 | -2.809039023 | -1.490076667 | 2.809039023 | down | 2.14791E-11 | 3.81402E-10 |
| 9213 | XPR1 | -2.403794113 | -1.265313333 | 2.403794113 | down | 1.28693E-12 | 3.53501E-11 |
| 84154 | RPF2 | -3.04442548 | -1.60617 | 3.04442548 | down | 5.92574E-11 | 9.30121E-10 |
| 5471 | PPAT | -2.274868786 | -1.185783333 | 2.274868786 | down | 1.88111E-14 | 1.19011E-12 |
| 56916 | SMARCAD1 | -2.004005776 | -1.002886667 | 2.004005776 | down | 9.54315E-13 | 2.75648E-11 |
| 26190 | FBXW2 | -2.377842787 | -1.249653333 | 2.377842787 | down | 3.88498E-09 | 3.68532E-08 |
| 11343 | MGLL | -2.627019058 | -1.393426667 | 2.627019058 | down | 4.77264E-16 | 7.81204E-14 |
| 55719 | SLF2 | -2.526143625 | -1.336936667 | 2.526143625 | down | 1.4144E-14 | 9.63191E-13 |
| 10513 | APPBP2 | -2.135089677 | -1.094296667 | 2.135089677 | down | 6.37395E-11 | 9.88605E-10 |
| 4739 | NEDD9 | -2.216684326 | -1.148403333 | 2.216684326 | down | 1.13336E-11 | 2.23062E-10 |
| 151987 | PPP4R2 | -3.734046776 | -1.90074 | 3.734046776 | down | 5.68277E-14 | 2.81803E-12 |
| 51714 | SELT | -3.325958114 | -1.73377 | 3.325958114 | down | 9.02355E-17 | 2.43243E-14 |
| 7485 | WRB | -2.616533385 | -1.387656667 | 2.616533385 | down | 5.51549E-15 | 4.85079E-13 |
| 5601 | MAPK9 | -2.599192225 | -1.378063333 | 2.599192225 | down | 9.0411E-13 | 2.62889E-11 |
| 6717 | SRI | -6.691996388 | -2.742436667 | 6.691996388 | down | 5.30582E-17 | 1.67839E-14 |
| 23484 | LEPROTL1 | -2.392899822 | -1.25876 | 2.392899822 | down | 1.96325E-15 | 2.15334E-13 |
| 80777 | CYB5B | -4.256211963 | -2.08957 | 4.256211963 | down | 1.50102E-12 | 4.03272E-11 |
| 9474 | ATG5 | -5.270622384 | -2.397973333 | 5.270622384 | down | 1.74853E-15 | 1.9596E-13 |
| 64747 | MFSD1 | -2.834779786 | -1.503236667 | 2.834779786 | down | 7.14566E-18 | 4.5949E-15 |
| 132430 | PABPC4L | -2.09208287 | -1.06494 | 2.09208287 | down | 3.81299E-08 | 2.8257E-07 |
| 286144 | TRIQK | -2.133546174 | -1.093253333 | 2.133546174 | down | 1.28882E-10 | 1.83366E-09 |
| 27069 | GHITM | -2.03883732 | -1.027746667 | 2.03883732 | down | 8.28477E-09 | 7.23764E-08 |
| 8821 | INPP4B | -2.818700123 | -1.49503 | 2.818700123 | down | 4.75963E-12 | 1.07606E-10 |
| 3184 | HNRNPD | -3.535162829 | -1.821776667 | 3.535162829 | down | 8.96895E-17 | 2.43243E-14 |
| 83693 | HSDL1 | -2.491094929 | -1.31678 | 2.491094929 | down | 3.1364E-13 | 1.10336E-11 |
| 79685 | SAP30L | -3.036424155 | -1.602373333 | 3.036424155 | down | 3.63985E-12 | 8.57496E-11 |
| 25782 | RAB3GAP2 | -2.030896428 | -1.022116667 | 2.030896428 | down | 3.6006E-12 | 8.4978E-11 |
| 4082 | MARCKS | -5.836094861 | -2.545003333 | 5.836094861 | down | 1.41883E-15 | 1.69676E-13 |
| 90861 | HN1L | -2.515909451 | -1.33108 | 2.515909451 | down | 2.45902E-14 | 1.46811E-12 |
| 79677 | SMC6 | -3.130818771 | -1.64654 | 3.130818771 | down | 1.22992E-13 | 5.24389E-12 |
| 56255 | TMX4 | -2.075928373 | -1.053756667 | 2.075928373 | down | 1.84283E-12 | 4.82866E-11 |
| 10972 | TMED10 | -2.066744432 | -1.04736 | 2.066744432 | down | 7.68526E-14 | 3.58022E-12 |
| 65117 | RSRC2 | -2.397825492 | -1.261726667 | 2.397825492 | down | 3.94616E-12 | 9.19716E-11 |
| 7905 | REEP5 | -4.832545558 | -2.272783333 | 4.832545558 | down | 4.61684E-16 | 7.66812E-14 |
| 5356 | PLRG1 | -2.885130681 | -1.528636667 | 2.885130681 | down | 3.29583E-14 | 1.84684E-12 |
| 9975 | NR1D2 | -2.030385024 | -1.021753333 | 2.030385024 | down | 6.47391E-09 | 5.79505E-08 |
| 55149 | MTPAP | -2.785719505 | -1.47805 | 2.785719505 | down | 6.5506E-13 | 2.01685E-11 |
| 8545 | CGGBP1 | -2.232504887 | -1.158663333 | 2.232504887 | down | 1.25362E-14 | 8.81242E-13 |
| 5210 | PFKFB4 | -2.077996657 | -1.055193333 | 2.077996657 | down | 7.36288E-13 | 2.21819E-11 |
| 10923 | SUB1 | -8.216268528 | -3.038483333 | 8.216268528 | down | 3.50851E-19 | 6.5534E-16 |
| 57488 | ESYT2 | -4.775769917 | -2.255733333 | 4.775769917 | down | 6.64143E-17 | 1.98863E-14 |
| 23216 | TBC1D1 | -2.566934826 | -1.360046667 | 2.566934826 | down | 4.02401E-10 | 4.98081E-09 |
| 23198 | PSME4 | -3.567582189 | -1.834946667 | 3.567582189 | down | 2.83369E-15 | 2.86473E-13 |
| 9204 | ZMYM6 | -3.59580138 | -1.846313333 | 3.59580138 | down | 3.47984E-13 | 1.20367E-11 |
| 161742 | SPRED1 | -2.303200488 | -1.20364 | 2.303200488 | down | 6.24249E-10 | 7.31804E-09 |
| 166929 | SGMS2 | -2.198277221 | -1.136373333 | 2.198277221 | down | 2.04211E-12 | 5.25257E-11 |
| 64786 | TBC1D15 | -2.599546568 | -1.37826 | 2.599546568 | down | 5.81949E-13 | 1.82873E-11 |
| 9282 | MED14 | -2.610048593 | -1.384076667 | 2.610048593 | down | 1.29117E-13 | 5.44632E-12 |
| 861 | RUNX1 | -2.461122838 | -1.299316667 | 2.461122838 | down | 4.84863E-13 | 1.5757E-11 |
| 55591 | VEZT | -4.485358605 | -2.165223333 | 4.485358605 | down | 6.53714E-16 | 9.42717E-14 |
| 9650 | MTFR1 | -2.047315342 | -1.033733333 | 2.047315342 | down | 4.53729E-15 | 4.16804E-13 |
| 5255 | PHKA1 | -5.316560957 | -2.410493333 | 5.316560957 | down | 3.21179E-15 | 3.17336E-13 |
| 55131 | RBM28 | -2.627741452 | -1.393823333 | 2.627741452 | down | 4.66559E-13 | 1.52761E-11 |
| 8065 | CUL5 | -2.378853895 | -1.250266667 | 2.378853895 | down | 6.76716E-15 | 5.57651E-13 |
| 64115 | C10orf54 | -2.090811984 | -1.064063333 | 2.090811984 | down | 6.67379E-16 | 9.54602E-14 |
| 7110 | TMF1 | -2.274621764 | -1.185626667 | 2.274621764 | down | 5.40243E-10 | 6.45674E-09 |
| 90355 | C5orf30 | -2.899913766 | -1.53601 | 2.899913766 | down | 1.4261E-13 | 5.89821E-12 |
| 153642 | ARSK | -2.100122283 | -1.070473333 | 2.100122283 | down | 1.41849E-09 | 1.51774E-08 |
| 9801 | MRPL19 | -2.701030629 | -1.43351 | 2.701030629 | down | 4.26005E-14 | 2.25507E-12 |
| 23065 | EMC1 | -2.470163722 | -1.304606667 | 2.470163722 | down | 2.69829E-09 | 2.68071E-08 |
| 7175 | TPR | -3.767550022 | -1.913626667 | 3.767550022 | down | 2.1549E-11 | 3.82164E-10 |
| 79139 | DERL1 | -2.315039271 | -1.211036667 | 2.315039271 | down | 2.21056E-14 | 1.34225E-12 |
| 7072 | TIA1 | -3.09468664 | -1.629793333 | 3.09468664 | down | 3.90856E-13 | 1.31374E-11 |
| 339761 | CYP27C1 | -3.192522232 | -1.674696667 | 3.192522232 | down | 5.9111E-11 | 9.28565E-10 |
| 50804 | MYEF2 | -2.722030691 | -1.444683333 | 2.722030691 | down | 6.917E-13 | 2.10815E-11 |
| 9318 | COPS2 | -2.02716942 | -1.019466667 | 2.02716942 | down | 1.90791E-11 | 3.44558E-10 |
| 54778 | RNF111 | -2.111717235 | -1.078416667 | 2.111717235 | down | 4.1532E-08 | 3.04949E-07 |
| 285672 | SREK1IP1 | -3.506884704 | -1.81019 | 3.506884704 | down | 6.82021E-14 | 3.25889E-12 |
| 23283 | CSTF2T | -2.101190063 | -1.071206667 | 2.101190063 | down | 1.01023E-09 | 1.12767E-08 |
| 51473 | DCDC2 | -4.698824876 | -2.2323 | 4.698824876 | down | 4.90282E-14 | 2.51719E-12 |
| 6167 | RPL37 | -2.170278218 | -1.11788 | 2.170278218 | down | 8.75207E-10 | 9.89622E-09 |
| 5810 | RAD1 | -3.010048362 | -1.589786667 | 3.010048362 | down | 3.82664E-11 | 6.32532E-10 |
| 7855 | FZD5 | -2.258001848 | -1.175046667 | 2.258001848 | down | 1.11496E-11 | 2.19991E-10 |
| 3725 | JUN | -3.524177689 | -1.817286667 | 3.524177689 | down | 1.70976E-17 | 7.38355E-15 |
| 25909 | AHCTF1 | -4.967362386 | -2.31248 | 4.967362386 | down | 5.38617E-15 | 4.75839E-13 |
| 7105 | TSPAN6 | -2.226647731 | -1.154873333 | 2.226647731 | down | 8.75559E-15 | 6.74731E-13 |
| 4288 | MKI67 | -3.014648923 | -1.59199 | 3.014648923 | down | 2.65103E-13 | 9.6642E-12 |
| 54801 | HAUS6 | -2.382506252 | -1.25248 | 2.382506252 | down | 5.386E-14 | 2.7097E-12 |
| 49854 | ZBTB21 | -3.433904697 | -1.77985 | 3.433904697 | down | 9.86187E-11 | 1.44179E-09 |
| 11169 | WDHD1 | -3.023333228 | -1.59614 | 3.023333228 | down | 2.153E-11 | 3.82134E-10 |
| 8975 | USP13 | -2.333787709 | -1.222673333 | 2.333787709 | down | 5.60152E-08 | 3.99417E-07 |
| 140707 | BRI3BP | -4.027208035 | -2.00978 | 4.027208035 | down | 4.35285E-13 | 1.44207E-11 |
| 4087 | SMAD2 | -2.839847253 | -1.505813333 | 2.839847253 | down | 4.91367E-14 | 2.51946E-12 |
| 7321 | UBE2D1 | -3.791669804 | -1.922833333 | 3.791669804 | down | 4.42069E-16 | 7.48332E-14 |
| 55254 | TMEM39A | -2.117497109 | -1.08236 | 2.117497109 | down | 7.50919E-11 | 1.13769E-09 |
| 3301 | DNAJA1 | -2.421985117 | -1.27619 | 2.421985117 | down | 7.3297E-18 | 4.63722E-15 |
| 100529241 | HSPE1-MOB4 | -3.387693453 | -1.760303333 | 3.387693453 | down | 9.13204E-16 | 1.21015E-13 |
| 120534 | ARL14EP | -2.16806799 | -1.11641 | 2.16806799 | down | 3.62604E-13 | 1.24655E-11 |
| 140739 | UBE2F | -2.567741552 | -1.3605 | 2.567741552 | down | 8.3314E-10 | 9.46147E-09 |
| 55105 | GPATCH2 | -8.156838003 | -3.02801 | 8.156838003 | down | 8.10988E-17 | 2.28856E-14 |
| 2181 | ACSL3 | -4.847452181 | -2.277226667 | 4.847452181 | down | 1.67248E-17 | 7.37114E-15 |
| 57650 | KIAA1524 | -2.726298128 | -1.446943333 | 2.726298128 | down | 9.18749E-13 | 2.66356E-11 |
| 54454 | ATAD2B | -3.289169448 | -1.717723333 | 3.289169448 | down | 8.95916E-10 | 1.01129E-08 |
| 54462 | CCSER2 | -2.506921231 | -1.325916667 | 2.506921231 | down | 1.58997E-14 | 1.04642E-12 |
| 55109 | AGGF1 | -2.024655803 | -1.017676667 | 2.024655803 | down | 1.35834E-11 | 2.5827E-10 |
| 966 | CD59 | -2.108923359 | -1.076506667 | 2.108923359 | down | 1.29601E-10 | 1.84055E-09 |
| 2060 | EPS15 | -2.757370886 | -1.463293333 | 2.757370886 | down | 1.26435E-13 | 5.35644E-12 |
| 3308 | HSPA4 | -3.766566498 | -1.91325 | 3.766566498 | down | 7.87832E-17 | 2.25567E-14 |
| 54482 | TRMT13 | -2.050155492 | -1.035733333 | 2.050155492 | down | 1.88306E-11 | 3.41042E-10 |
| 26225 | ARL5A | -3.543217432 | -1.82506 | 3.543217432 | down | 4.94538E-17 | 1.57709E-14 |
| 3146 | HMGB1 | -2.562934603 | -1.357796667 | 2.562934603 | down | 2.73838E-14 | 1.59366E-12 |
| 6642 | SNX1 | -2.148639331 | -1.103423333 | 2.148639331 | down | 1.47253E-11 | 2.76364E-10 |
| 1540 | CYLD | -2.513027862 | -1.329426667 | 2.513027862 | down | 6.77061E-12 | 1.45124E-10 |
| 8540 | AGPS | -3.413464308 | -1.771236667 | 3.413464308 | down | 1.56078E-14 | 1.02893E-12 |
| 25843 | MOB4 | -3.387693453 | -1.760303333 | 3.387693453 | down | 9.13204E-16 | 1.21015E-13 |
| 7091 | TLE4 | -2.209142782 | -1.143486667 | 2.209142782 | down | 6.87073E-12 | 1.46869E-10 |
| 5912 | RAP2B | -2.232680272 | -1.158776667 | 2.232680272 | down | 1.01924E-08 | 8.70639E-08 |
| 7334 | UBE2N | -4.875747224 | -2.285623333 | 4.875747224 | down | 1.00881E-16 | 2.63803E-14 |
| 9958 | USP15 | -3.907023681 | -1.96607 | 3.907023681 | down | 2.50624E-15 | 2.58024E-13 |
| 26973 | CHORDC1 | -2.032065164 | -1.022946667 | 2.032065164 | down | 2.22278E-08 | 1.74134E-07 |
| 79796 | ALG9 | -2.722967947 | -1.44518 | 2.722967947 | down | 6.35197E-11 | 9.86067E-10 |
| 7026 | NR2F2 | -2.459889199 | -1.298593333 | 2.459889199 | down | 1.96895E-09 | 2.02868E-08 |
| 9097 | USP14 | -4.56597618 | -2.190923333 | 4.56597618 | down | 3.24524E-17 | 1.16784E-14 |
| 23051 | ZHX3 | -2.745419608 | -1.457026667 | 2.745419608 | down | 3.7531E-10 | 4.692E-09 |
| 128 | ADH5 | -2.724547544 | -1.446016667 | 2.724547544 | down | 1.11114E-14 | 8.12422E-13 |
| 51768 | TM7SF3 | -2.17787326 | -1.12292 | 2.17787326 | down | 7.53385E-13 | 2.26102E-11 |
| 64376 | IKZF5 | -2.738052292 | -1.45315 | 2.738052292 | down | 5.79664E-12 | 1.27237E-10 |
| 51762 | RAB8B | -2.099181145 | -1.069826667 | 2.099181145 | down | 3.52941E-12 | 8.35963E-11 |
| 1787 | TRDMT1 | -2.680551773 | -1.42253 | 2.680551773 | down | 1.89785E-09 | 1.9642E-08 |
| 528 | ATP6V1C1 | -4.045187757 | -2.016206667 | 4.045187757 | down | 4.53151E-16 | 7.62869E-14 |
| 25852 | ARMC8 | -2.938900808 | -1.555276667 | 2.938900808 | down | 3.63129E-11 | 6.0406E-10 |
| 57403 | RAB22A | -2.283805834 | -1.19144 | 2.283805834 | down | 2.74166E-13 | 9.91168E-12 |
| 7341 | SUMO1 | -2.354874214 | -1.23565 | 2.354874214 | down | 1.70783E-08 | 1.37499E-07 |
| 284273 | ZADH2 | -2.794331793 | -1.482503333 | 2.794331793 | down | 4.20835E-12 | 9.71016E-11 |
| 8876 | VNN1 | -2.338969961 | -1.225873333 | 2.338969961 | down | 1.21005E-11 | 2.34739E-10 |
| 4801 | NFYB | -2.365568056 | -1.242186667 | 2.365568056 | down | 1.90254E-10 | 2.57423E-09 |
| 10944 | C11orf58 | -3.910554888 | -1.967373333 | 3.910554888 | down | 2.44427E-15 | 2.52972E-13 |
| 79582 | SPAG16 | -2.949090304 | -1.56027 | 2.949090304 | down | 1.08139E-12 | 3.06166E-11 |
| 4175 | MCM6 | -2.528339152 | -1.33819 | 2.528339152 | down | 4.32943E-10 | 5.31192E-09 |
| 10102 | TSFM | -2.010610091 | -1.007633333 | 2.010610091 | down | 7.40528E-08 | 5.14528E-07 |
| 5529 | PPP2R5E | -3.146286395 | -1.65365 | 3.146286395 | down | 3.46908E-14 | 1.92196E-12 |
| 5927 | KDM5A | -3.510654449 | -1.81174 | 3.510654449 | down | 7.54113E-13 | 2.26147E-11 |
| 81839 | VANGL1 | -2.099525533 | -1.070063333 | 2.099525533 | down | 1.25861E-12 | 3.4816E-11 |
| 54205 | CYCS | -2.849884268 | -1.510903333 | 2.849884268 | down | 2.07896E-10 | 2.78128E-09 |
| 60496 | AASDHPPT | -2.930323615 | -1.55106 | 2.930323615 | down | 6.58904E-16 | 9.46721E-14 |
| 23768 | FLRT2 | -2.510572986 | -1.328016667 | 2.510572986 | down | 9.58411E-11 | 1.41011E-09 |
| 4718 | NDUFC2 | -2.513347231 | -1.32961 | 2.513347231 | down | 1.35921E-11 | 2.58309E-10 |
| 1073 | CFL2 | -3.075419756 | -1.620783333 | 3.075419756 | down | 7.63396E-18 | 4.75305E-15 |
| 55860 | ACTR10 | -3.034327206 | -1.601376667 | 3.034327206 | down | 5.34027E-13 | 1.70164E-11 |
| 10949 | HNRNPA0 | -4.596874672 | -2.200653333 | 4.596874672 | down | 9.30543E-19 | 1.52086E-15 |
| 26031 | OSBPL3 | -3.886352243 | -1.958416667 | 3.886352243 | down | 9.77391E-13 | 2.81691E-11 |
| 3093 | UBE2K | -2.243923253 | -1.166023333 | 2.243923253 | down | 4.07997E-11 | 6.6933E-10 |
| 6921 | TCEB1 | -2.285547813 | -1.19254 | 2.285547813 | down | 9.4416E-15 | 7.16338E-13 |
| 1347 | COX7A2 | -4.584856775 | -2.196876667 | 4.584856775 | down | 2.43643E-18 | 2.38923E-15 |
| 9169 | SCAF11 | -3.34865868 | -1.743583333 | 3.34865868 | down | 2.83649E-16 | 5.55937E-14 |
| 27436 | EML4 | -2.603922782 | -1.380686667 | 2.603922782 | down | 4.33682E-15 | 4.01207E-13 |
| 25758 | KIAA1549L | -2.779457521 | -1.474803333 | 2.779457521 | down | 2.79013E-12 | 6.8962E-11 |
| 54014 | BRWD1 | -3.467165412 | -1.793756667 | 3.467165412 | down | 2.0068E-12 | 5.18899E-11 |
| 55156 | ARMC1 | -2.083193232 | -1.058796667 | 2.083193232 | down | 5.02769E-16 | 8.07178E-14 |
| 30061 | SLC40A1 | -2.067737911 | -1.048053333 | 2.067737911 | down | 2.76979E-10 | 3.5821E-09 |
| 10169 | SERF2 | -2.477823342 | -1.309073333 | 2.477823342 | down | 1.61551E-09 | 1.69888E-08 |
| 3920 | LAMP2 | -3.556841451 | -1.830596667 | 3.556841451 | down | 8.35817E-14 | 3.81664E-12 |
| 2218 | FKTN | -2.412078177 | -1.270276667 | 2.412078177 | down | 4.32006E-13 | 1.43241E-11 |
| 285550 | FAM200B | -2.568346763 | -1.36084 | 2.568346763 | down | 1.72416E-09 | 1.80444E-08 |
| 51526 | OSER1 | -2.179468975 | -1.123976667 | 2.179468975 | down | 8.35198E-11 | 1.24898E-09 |
| 27342 | RABGEF1 | -3.256795952 | -1.703453333 | 3.256795952 | down | 2.48804E-16 | 5.10959E-14 |
| 54665 | RSBN1 | -3.03495123 | -1.601673333 | 3.03495123 | down | 2.42352E-12 | 6.10549E-11 |
| 94101 | ORMDL1 | -3.700738852 | -1.887813333 | 3.700738852 | down | 7.61151E-13 | 2.28084E-11 |
| 51528 | JKAMP | -3.376058727 | -1.75534 | 3.376058727 | down | 3.37913E-16 | 6.32844E-14 |
| 2054 | STX2 | -2.169616417 | -1.11744 | 2.169616417 | down | 3.03805E-14 | 1.73461E-12 |
| 860 | RUNX2 | -2.317051322 | -1.21229 | 2.317051322 | down | 2.09623E-10 | 2.79866E-09 |
| 26273 | FBXO3 | -2.782663902 | -1.476466667 | 2.782663902 | down | 1.76884E-14 | 1.14119E-12 |
| 55660 | PRPF40A | -2.876677184 | -1.524403333 | 2.876677184 | down | 1.05064E-13 | 4.62009E-12 |
| 55809 | TRERF1 | -2.140898897 | -1.098216667 | 2.140898897 | down | 5.11697E-10 | 6.14929E-09 |
| 1364 | CLDN4 | -3.56156997 | -1.832513333 | 3.56156997 | down | 1.17052E-11 | 2.28653E-10 |
| 8871 | SYNJ2 | -2.218739051 | -1.14974 | 2.218739051 | down | 3.08061E-12 | 7.49143E-11 |
| 79109 | MAPKAP1 | -2.552569071 | -1.35195 | 2.552569071 | down | 1.95749E-13 | 7.57225E-12 |
| 55071 | C9orf40 | -3.223780089 | -1.688753333 | 3.223780089 | down | 1.42972E-13 | 5.89883E-12 |
| 4678 | NASP | -2.430809216 | -1.281436667 | 2.430809216 | down | 4.35885E-13 | 1.44284E-11 |
| 84230 | LRRC8C | -3.479122153 | -1.798723333 | 3.479122153 | down | 1.68115E-13 | 6.68792E-12 |
| 51082 | POLR1D | -2.322362744 | -1.215593333 | 2.322362744 | down | 1.35688E-13 | 5.66811E-12 |
| 51101 | ZC2HC1A | -3.733865603 | -1.90067 | 3.733865603 | down | 4.29725E-12 | 9.86882E-11 |
| 8601 | RGS20 | -2.146401548 | -1.10192 | 2.146401548 | down | 2.05571E-14 | 1.27185E-12 |
| 5000 | ORC4 | -2.59740923 | -1.377073333 | 2.59740923 | down | 2.55708E-10 | 3.34004E-09 |
| 57606 | SLAIN2 | -2.618565453 | -1.388776667 | 2.618565453 | down | 2.34552E-12 | 5.93952E-11 |
| 64841 | GNPNAT1 | -2.181801725 | -1.12552 | 2.181801725 | down | 8.59038E-15 | 6.67243E-13 |
| 9824 | ARHGAP11A | -2.176736335 | -1.122166667 | 2.176736335 | down | 2.47692E-10 | 3.24615E-09 |
| 5128 | CDK17 | -2.988952562 | -1.57964 | 2.988952562 | down | 2.00502E-15 | 2.19072E-13 |
| 283742 | FAM98B | -2.078774596 | -1.055733333 | 2.078774596 | down | 7.70652E-14 | 3.58586E-12 |
| 23350 | U2SURP | -2.096166526 | -1.067753333 | 2.096166526 | down | 4.98199E-13 | 1.60473E-11 |
| 6747 | SSR3 | -2.149428817 | -1.103953333 | 2.149428817 | down | 1.50034E-11 | 2.80656E-10 |
| 22868 | FASTKD2 | -2.838390986 | -1.505073333 | 2.838390986 | down | 3.24623E-12 | 7.81665E-11 |
| 55183 | RIF1 | -3.796035103 | -1.924493333 | 3.796035103 | down | 9.04494E-14 | 4.05472E-12 |
| 57707 | TLDC1 | -2.000970642 | -1.0007 | 2.000970642 | down | 9.76655E-11 | 1.43052E-09 |
| 11231 | SEC63 | -5.209145255 | -2.381046667 | 5.209145255 | down | 2.79057E-14 | 1.61302E-12 |
| 138050 | HGSNAT | -2.366049079 | -1.24248 | 2.366049079 | down | 8.89678E-12 | 1.81026E-10 |
| 7187 | TRAF3 | -2.09855557 | -1.069396667 | 2.09855557 | down | 5.23197E-15 | 4.67379E-13 |
| 11052 | CPSF6 | -2.638729258 | -1.399843333 | 2.638729258 | down | 2.26671E-15 | 2.40953E-13 |
| 25842 | ASF1A | -2.041500612 | -1.02963 | 2.041500612 | down | 4.39492E-12 | 1.00461E-10 |
| 57472 | CNOT6 | -2.958528925 | -1.56488 | 2.958528925 | down | 9.02809E-15 | 6.93007E-13 |
| 125228 | FAM210A | -5.108441915 | -2.352883333 | 5.108441915 | down | 1.06119E-15 | 1.35587E-13 |
| 22998 | LIMCH1 | -3.397114865 | -1.76431 | 3.397114865 | down | 3.83385E-15 | 3.62368E-13 |
| 55781 | RIOK2 | -3.497102301 | -1.80616 | 3.497102301 | down | 2.591E-13 | 9.46295E-12 |
| 57520 | HECW2 | -2.021799593 | -1.01564 | 2.021799593 | down | 8.3551E-09 | 7.29258E-08 |
| 647087 | C7orf73 | -4.129260207 | -2.045883333 | 4.129260207 | down | 1.68041E-13 | 6.68792E-12 |
| 91298 | C12orf29 | -2.66425099 | -1.41373 | 2.66425099 | down | 1.74275E-15 | 1.95872E-13 |
| 1655 | DDX5 | -6.010493013 | -2.587483333 | 6.010493013 | down | 2.91929E-17 | 1.08693E-14 |
| 135228 | CD109 | -2.435075774 | -1.283966667 | 2.435075774 | down | 4.24226E-17 | 1.42224E-14 |
| 261726 | TIPRL | -3.711759752 | -1.892103333 | 3.711759752 | down | 2.29217E-15 | 2.43001E-13 |
| 171546 | SPTSSA | -2.449442344 | -1.292453333 | 2.449442344 | down | 3.28306E-12 | 7.88599E-11 |
| 3309 | HSPA5 | -2.976864043 | -1.573793333 | 2.976864043 | down | 2.5727E-11 | 4.46919E-10 |
| 8766 | RAB11A | -2.174152795 | -1.120453333 | 2.174152795 | down | 4.57839E-14 | 2.3945E-12 |
| 26009 | ZZZ3 | -2.012543547 | -1.00902 | 2.012543547 | down | 2.37981E-08 | 1.84921E-07 |
| 7716 | VEZF1 | -2.544178687 | -1.3472 | 2.544178687 | down | 1.25589E-12 | 3.47653E-11 |
| 55187 | VPS13D | -3.47863184 | -1.79852 | 3.47863184 | down | 1.76882E-13 | 6.99414E-12 |
| 6157 | RPL27A | -3.576561708 | -1.838573333 | 3.576561708 | down | 7.86001E-12 | 1.63733E-10 |
| 54149 | C21orf91 | -5.035540589 | -2.332146667 | 5.035540589 | down | 6.1697E-13 | 1.91461E-11 |
| 83892 | KCTD10 | -2.131782129 | -1.09206 | 2.131782129 | down | 1.15334E-13 | 4.98783E-12 |
| 10336 | PCGF3 | -4.128535184 | -2.04563 | 4.128535184 | down | 9.42654E-16 | 1.23252E-13 |
| 54968 | TMEM70 | -2.430792367 | -1.281426667 | 2.430792367 | down | 3.45087E-08 | 2.58469E-07 |
| 1459 | CSNK2A2 | -2.014534715 | -1.010446667 | 2.014534715 | down | 6.65333E-13 | 2.04688E-11 |
| 22990 | PCNX | -2.59181007 | -1.37396 | 2.59181007 | down | 9.3565E-12 | 1.88394E-10 |
| 157 | ADRBK2 | -2.86125881 | -1.51665 | 2.86125881 | down | 1.99093E-11 | 3.57248E-10 |
| 5478 | PPIA | -4.759323982 | -2.250756667 | 4.759323982 | down | 1.54157E-17 | 7.1986E-15 |
| 84945 | ABHD13 | -3.037989043 | -1.603116667 | 3.037989043 | down | 1.92914E-11 | 3.47911E-10 |
| 55681 | SCYL2 | -4.335164285 | -2.116086667 | 4.335164285 | down | 5.06422E-16 | 8.07178E-14 |
| 26135 | SERBP1 | -2.327686342 | -1.218896667 | 2.327686342 | down | 5.42339E-14 | 2.72036E-12 |
| 145173 | B3GLCT | -2.732244642 | -1.450086667 | 2.732244642 | down | 4.409E-11 | 7.14113E-10 |
| 9939 | RBM8A | -2.667453891 | -1.415463333 | 2.667453891 | down | 2.68052E-16 | 5.31027E-14 |
| 10914 | PAPOLA | -3.760288447 | -1.910843333 | 3.760288447 | down | 3.22953E-14 | 1.82717E-12 |
| 51566 | ARMCX3 | -2.254764357 | -1.172976667 | 2.254764357 | down | 2.60131E-11 | 4.5089E-10 |
| 2926 | GRSF1 | -2.20012679 | -1.137586667 | 2.20012679 | down | 1.59448E-14 | 1.04763E-12 |
| 10614 | HEXIM1 | -2.488667228 | -1.315373333 | 2.488667228 | down | 2.92035E-11 | 5.01316E-10 |
| 30011 | SH3KBP1 | -4.140782236 | -2.049903333 | 4.140782236 | down | 4.25455E-16 | 7.31951E-14 |
| 56995 | TULP4 | -2.023874736 | -1.01712 | 2.023874736 | down | 3.28188E-12 | 7.88599E-11 |
| 55031 | USP47 | -3.521353346 | -1.81613 | 3.521353346 | down | 1.9239E-12 | 5.00431E-11 |
| 64398 | MPP5 | -2.788636711 | -1.47956 | 2.788636711 | down | 3.32867E-15 | 3.24945E-13 |
| 83640 | FAM103A1 | -2.602683711 | -1.38 | 2.602683711 | down | 2.64458E-14 | 1.5555E-12 |
| 5537 | PPP6C | -6.050216073 | -2.596986667 | 6.050216073 | down | 3.57244E-15 | 3.42613E-13 |
| 4673 | NAP1L1 | -5.204249035 | -2.37969 | 5.204249035 | down | 1.46533E-14 | 9.89288E-13 |
| 1182 | CLCN3 | -2.008093809 | -1.005826667 | 2.008093809 | down | 1.80048E-13 | 7.08362E-12 |
| 5494 | PPM1A | -5.681190711 | -2.506193333 | 5.681190711 | down | 1.16711E-14 | 8.42608E-13 |
| 92092 | ZC3HAV1L | -3.297090148 | -1.721193333 | 3.297090148 | down | 8.72254E-12 | 1.78385E-10 |
| 57619 | SHROOM3 | -4.367062968 | -2.126663333 | 4.367062968 | down | 1.78231E-14 | 1.14608E-12 |
| 5861 | RAB1A | -2.61924316 | -1.38915 | 2.61924316 | down | 6.53535E-15 | 5.46587E-13 |
| 7323 | UBE2D3 | -2.199150999 | -1.136946667 | 2.199150999 | down | 1.40444E-15 | 1.68468E-13 |
| 55219 | TMEM57 | -2.016048016 | -1.01153 | 2.016048016 | down | 2.09155E-08 | 1.6494E-07 |
| 2773 | GNAI3 | -2.687273822 | -1.426143333 | 2.687273822 | down | 2.8766E-13 | 1.03141E-11 |
| 79047 | KCTD15 | -2.187616729 | -1.12936 | 2.187616729 | down | 2.91504E-13 | 1.03948E-11 |
| 56474 | CTPS2 | -2.062560827 | -1.044436667 | 2.062560827 | down | 4.51959E-13 | 1.48806E-11 |
| 84376 | HOOK3 | -3.503847536 | -1.80894 | 3.503847536 | down | 3.14577E-14 | 1.78571E-12 |
| 131118 | DNAJC19 | -2.962318301 | -1.566726667 | 2.962318301 | down | 3.37712E-13 | 1.1754E-11 |
| 22948 | CCT5 | -3.963074258 | -1.98662 | 3.963074258 | down | 5.78217E-14 | 2.84574E-12 |
| 7109 | TRAPPC10 | -2.465733136 | -1.302016667 | 2.465733136 | down | 6.92535E-09 | 6.16259E-08 |
| 9391 | CIAO1 | -2.354836128 | -1.235626667 | 2.354836128 | down | 3.47981E-12 | 8.25745E-11 |
| 4205 | MEF2A | -2.628706977 | -1.394353333 | 2.628706977 | down | 6.09139E-11 | 9.51174E-10 |
| 3836 | KPNA1 | -2.380272368 | -1.251126667 | 2.380272368 | down | 9.67567E-09 | 8.32482E-08 |
| 23229 | ARHGEF9 | -2.833830234 | -1.502753333 | 2.833830234 | down | 2.12829E-12 | 5.43505E-11 |
| 283209 | PGM2L1 | -2.18015393 | -1.12443 | 2.18015393 | down | 2.05076E-14 | 1.27185E-12 |
| 157247 | MGC27345 | -2.627741452 | -1.393823333 | 2.627741452 | down | 4.66559E-13 | 1.52761E-11 |
| 79657 | RPAP3 | -3.420506567 | -1.77421 | 3.420506567 | down | 1.03628E-13 | 4.5672E-12 |
| 2009 | EML1 | -2.751255263 | -1.46009 | 2.751255263 | down | 3.96192E-14 | 2.12885E-12 |
| 3614 | IMPDH1 | -2.08816638 | -1.062236667 | 2.08816638 | down | 5.6023E-15 | 4.90514E-13 |
| 10605 | PAIP1 | -2.274201364 | -1.18536 | 2.274201364 | down | 1.9619E-10 | 2.64453E-09 |
| 81614 | NIPA2 | -2.257047322 | -1.174436667 | 2.257047322 | down | 7.28096E-15 | 5.91295E-13 |
| 51542 | VPS54 | -3.207835141 | -1.6816 | 3.207835141 | down | 1.20392E-14 | 8.55505E-13 |
| 5728 | PTEN | -2.086758046 | -1.061263333 | 2.086758046 | down | 1.05447E-09 | 1.16973E-08 |
| 283635 | FAM177A1 | -2.882738555 | -1.52744 | 2.882738555 | down | 1.66603E-13 | 6.67519E-12 |
| 8802 | SUCLG1 | -2.383750652 | -1.253233333 | 2.383750652 | down | 6.03445E-12 | 1.31209E-10 |
| 27042 | DIEXF | -2.02513301 | -1.018016667 | 2.02513301 | down | 7.04306E-12 | 1.49737E-10 |
| 54928 | IMPAD1 | -2.351378303 | -1.233506667 | 2.351378303 | down | 5.9581E-14 | 2.92865E-12 |
| 63875 | MRPL17 | -2.315600971 | -1.211386667 | 2.315600971 | down | 1.65506E-10 | 2.28109E-09 |
| 79768 | KATNBL1 | -4.052175522 | -2.018696667 | 4.052175522 | down | 3.70048E-16 | 6.689E-14 |
| 55038 | CDCA4 | -3.039379172 | -1.603776667 | 3.039379172 | down | 3.47645E-12 | 8.25448E-11 |
| 78988 | MRPL57 | -2.131782129 | -1.09206 | 2.131782129 | down | 3.15827E-09 | 3.07249E-08 |
| 84542 | KIAA1841 | -2.221467959 | -1.151513333 | 2.221467959 | down | 5.26328E-13 | 1.68121E-11 |
| 494115 | RBMXL1 | -2.028303206 | -1.020273333 | 2.028303206 | down | 6.96308E-11 | 1.06524E-09 |
| 10291 | SF3A1 | -2.250943742 | -1.17053 | 2.250943742 | down | 4.01023E-11 | 6.59544E-10 |
| 23585 | TMEM50A | -3.364627208 | -1.750446667 | 3.364627208 | down | 9.35963E-16 | 1.22786E-13 |
| 6146 | RPL22 | -3.826264658 | -1.935936667 | 3.826264658 | down | 4.18269E-20 | 1.82296E-16 |
| 55635 | DEPDC1 | -2.770551786 | -1.470173333 | 2.770551786 | down | 8.78125E-14 | 3.95459E-12 |
| 1244 | ABCC2 | -2.403866315 | -1.265356667 | 2.403866315 | down | 3.49181E-18 | 2.92438E-15 |
| 394 | ARHGAP5 | -2.452177367 | -1.294063333 | 2.452177367 | down | 1.44588E-08 | 1.18229E-07 |
| 54994 | GID8 | -2.24177787 | -1.164643333 | 2.24177787 | down | 1.05075E-11 | 2.08687E-10 |
| 23023 | TMCC1 | -3.498718679 | -1.806826667 | 3.498718679 | down | 8.313E-17 | 2.32913E-14 |
| 3607 | FOXK2 | -2.01409258 | -1.01013 | 2.01409258 | down | 3.35228E-13 | 1.16883E-11 |
| 4763 | NF1 | -2.469804189 | -1.304396667 | 2.469804189 | down | 4.16059E-10 | 5.13044E-09 |
| 55832 | CAND1 | -2.449640432 | -1.29257 | 2.449640432 | down | 3.03114E-13 | 1.07501E-11 |
| 10929 | SRSF8 | -2.592954097 | -1.374596667 | 2.592954097 | down | 4.44309E-10 | 5.43267E-09 |
| 23012 | STK38L | -2.283088314 | -1.190986667 | 2.283088314 | down | 1.44434E-11 | 2.71981E-10 |
| 2297 | FOXD1 | -2.108022112 | -1.07589 | 2.108022112 | down | 1.25524E-12 | 3.47653E-11 |
| 6526 | SLC5A3 | -2.06618581 | -1.04697 | 2.06618581 | down | 1.70719E-13 | 6.78464E-12 |
| 1314 | COPA | -2.196885988 | -1.13546 | 2.196885988 | down | 5.41936E-10 | 6.47303E-09 |
| 9255 | AIMP1 | -2.068516788 | -1.048596667 | 2.068516788 | down | 1.39492E-11 | 2.64325E-10 |
| 112858 | TP53RK | -3.122734686 | -1.64281 | 3.122734686 | down | 4.02457E-12 | 9.34658E-11 |
| 5257 | PHKB | -2.098099843 | -1.069083333 | 2.098099843 | down | 2.30917E-11 | 4.05812E-10 |
| 6919 | TCEA2 | -2.093668937 | -1.066033333 | 2.093668937 | down | 1.47159E-10 | 2.06154E-09 |
| 81873 | ARPC5L | -2.344423711 | -1.229233333 | 2.344423711 | down | 6.41499E-12 | 1.38715E-10 |
| 56261 | GPCPD1 | -3.115794317 | -1.6396 | 3.115794317 | down | 4.37088E-11 | 7.08754E-10 |
| 11103 | KRR1 | -2.312697648 | -1.209576667 | 2.312697648 | down | 1.2825E-11 | 2.46477E-10 |
| 9889 | ZBED4 | -2.014646428 | -1.010526667 | 2.014646428 | down | 2.1022E-07 | 1.3185E-06 |
| 4094 | MAF | -2.068492892 | -1.04858 | 2.068492892 | down | 1.39821E-07 | 9.11347E-07 |
| 4683 | NBN | -2.692052726 | -1.428706667 | 2.692052726 | down | 5.32884E-15 | 4.71837E-13 |
| 9263 | STK17A | -2.37156049 | -1.245836667 | 2.37156049 | down | 4.38234E-12 | 1.00232E-10 |
| 55585 | UBE2Q1 | -2.432742012 | -1.282583333 | 2.432742012 | down | 4.34441E-12 | 9.95383E-11 |
| 1964 | EIF1AX | -5.794068202 | -2.534576667 | 5.794068202 | down | 3.26353E-18 | 2.90936E-15 |
| 7322 | UBE2D2 | -3.651071698 | -1.86832 | 3.651071698 | down | 1.33213E-11 | 2.54395E-10 |
| 150864 | FAM117B | -2.956410631 | -1.563846667 | 2.956410631 | down | 1.00179E-12 | 2.87878E-11 |
| 10490 | VTI1B | -2.788765577 | -1.479626667 | 2.788765577 | down | 1.304E-12 | 3.5669E-11 |
| 25959 | KANK2 | -2.082476189 | -1.0583 | 2.082476189 | down | 4.881E-14 | 2.50927E-12 |
| 116150 | NUS1 | -3.120873758 | -1.64195 | 3.120873758 | down | 2.99812E-12 | 7.3135E-11 |
| 3189 | HNRNPH3 | -2.371461861 | -1.245776667 | 2.371461861 | down | 1.86143E-15 | 2.0684E-13 |
| 8228 | PNPLA4 | -3.180190819 | -1.669113333 | 3.180190819 | down | 1.03188E-10 | 1.50075E-09 |
| 4719 | NDUFS1 | -3.20782773 | -1.681596667 | 3.20782773 | down | 1.36795E-12 | 3.72108E-11 |
| 55500 | ETNK1 | -2.396169558 | -1.26073 | 2.396169558 | down | 3.04614E-12 | 7.42142E-11 |
| 4193 | MDM2 | -2.304233095 | -1.204286667 | 2.304233095 | down | 1.33726E-11 | 2.55251E-10 |
| 8445 | DYRK2 | -3.801608587 | -1.92661 | 3.801608587 | down | 2.52936E-16 | 5.14063E-14 |
| 54764 | ZRANB1 | -2.092010366 | -1.06489 | 2.092010366 | down | 1.65646E-09 | 1.73962E-08 |
| 121457 | IKBIP | -2.222792586 | -1.152373333 | 2.222792586 | down | 2.2362E-11 | 3.94579E-10 |
| 56929 | FEM1C | -2.384009525 | -1.25339 | 2.384009525 | down | 1.08122E-10 | 1.56531E-09 |
| 3021 | H3F3B | -2.64223112 | -1.401756667 | 2.64223112 | down | 2.93388E-14 | 1.68002E-12 |
| 133686 | NADK2 | -2.133881409 | -1.09348 | 2.133881409 | down | 1.2359E-13 | 5.25795E-12 |
| 5718 | PSMD12 | -3.284514194 | -1.71568 | 3.284514194 | down | 2.1337E-15 | 2.29299E-13 |
| 831 | CAST | -2.097624828 | -1.068756667 | 2.097624828 | down | 1.07594E-15 | 1.36581E-13 |
| 56941 | HMCES | -2.000688645 | -1.000496667 | 2.000688645 | down | 4.90156E-08 | 3.54403E-07 |
| 10527 | IPO7 | -2.598465672 | -1.37766 | 2.598465672 | down | 1.90188E-15 | 2.10144E-13 |
| 23327 | NEDD4L | -2.850872133 | -1.511403333 | 2.850872133 | down | 5.40812E-13 | 1.71907E-11 |
| 5898 | RALA | -3.15922349 | -1.65957 | 3.15922349 | down | 9.7606E-18 | 5.67292E-15 |
| 23075 | SWAP70 | -2.745419608 | -1.457026667 | 2.745419608 | down | 2.83322E-14 | 1.63431E-12 |
| 127018 | LYPLAL1 | -3.67553264 | -1.877953333 | 3.67553264 | down | 7.74169E-16 | 1.05761E-13 |
| 1355 | COX15 | -2.688578012 | -1.426843333 | 2.688578012 | down | 1.32421E-11 | 2.53128E-10 |
| 950 | SCARB2 | -4.537643705 | -2.181943333 | 4.537643705 | down | 9.01627E-21 | 1.38642E-16 |
| 84640 | USP38 | -3.372160803 | -1.753673333 | 3.372160803 | down | 4.72087E-14 | 2.43653E-12 |
| 92259 | MRPS36 | -2.127108071 | -1.088893333 | 2.127108071 | down | 4.27865E-10 | 5.2529E-09 |
| 4686 | NCBP1 | -2.366388041 | -1.242686667 | 2.366388041 | down | 7.98217E-12 | 1.65837E-10 |
| 8732 | RNGTT | -2.547961244 | -1.349343333 | 2.547961244 | down | 4.34262E-12 | 9.95383E-11 |
| 4856 | NOV | -2.930973654 | -1.55138 | 2.930973654 | down | 5.10396E-12 | 1.14206E-10 |
| 55654 | TMEM127 | -2.133708855 | -1.093363333 | 2.133708855 | down | 8.75523E-13 | 2.56478E-11 |
| 159371 | SLC35G1 | -2.020090602 | -1.01442 | 2.020090602 | down | 1.18616E-10 | 1.69869E-09 |
| 84991 | RBM17 | -3.852842826 | -1.945923333 | 3.852842826 | down | 3.62256E-16 | 6.60495E-14 |
| 10123 | ARL4C | -2.193913532 | -1.133506667 | 2.193913532 | down | 2.86479E-16 | 5.56293E-14 |
| 157695 | TDRP | -2.310011446 | -1.2079 | 2.310011446 | down | 2.86539E-13 | 1.02926E-11 |
| 6617 | SNAPC1 | -2.941692948 | -1.556646667 | 2.941692948 | down | 8.27334E-11 | 1.23863E-09 |
| 51755 | CDK12 | -2.383976476 | -1.25337 | 2.383976476 | down | 4.47393E-10 | 5.46411E-09 |
| 2235 | FECH | -2.806930481 | -1.488993333 | 2.806930481 | down | 9.56877E-12 | 1.91987E-10 |
| 81542 | TMX1 | -4.436073697 | -2.149283333 | 4.436073697 | down | 4.20885E-17 | 1.42224E-14 |
| 81627 | TRMT1L | -2.540918321 | -1.34535 | 2.540918321 | down | 4.98496E-10 | 6.01277E-09 |
| 84312 | BRMS1L | -2.163619292 | -1.113446667 | 2.163619292 | down | 1.0707E-13 | 4.67685E-12 |
| 23313 | KIAA0930 | -2.804175545 | -1.487576667 | 2.804175545 | down | 2.23934E-13 | 8.4704E-12 |
| 222236 | NAPEPLD | -2.451033156 | -1.29339 | 2.451033156 | down | 9.97795E-13 | 2.86939E-11 |
| 444 | ASPH | -2.577692195 | -1.36608 | 2.577692195 | down | 3.14413E-07 | 1.89441E-06 |
| 29914 | UBIAD1 | -2.92058357 | -1.546256667 | 2.92058357 | down | 1.8791E-11 | 3.40765E-10 |
| 2909 | ARHGAP35 | 2.071118356 | 1.05041 | 2.071118356 | up | 6.35768E-11 | 9.86472E-10 |
| 29068 | ZBTB44 | 2.577573083 | 1.366013333 | 2.577573083 | up | 4.06861E-14 | 2.16542E-12 |
| 54852 | PAQR5 | 2.897288468 | 1.534703333 | 2.897288468 | up | 9.49249E-15 | 7.18808E-13 |
| 197131 | UBR1 | 3.127500251 | 1.64501 | 3.127500251 | up | 1.49328E-10 | 2.083E-09 |
| 9462 | RASAL2 | 2.1706443 | 1.118123333 | 2.1706443 | up | 9.05429E-11 | 1.34021E-09 |
| 3685 | ITGAV | 3.796745596 | 1.924763333 | 3.796745596 | up | 1.66654E-18 | 1.76676E-15 |
| 7544 | ZFY | 2.511031279 | 1.32828 | 2.511031279 | up | 3.04269E-13 | 1.07632E-11 |
| 342908 | ZNF404 | 2.039459229 | 1.028186667 | 2.039459229 | up | 1.35451E-08 | 1.11431E-07 |
| 405 | ARNT | 2.02226211 | 1.01597 | 2.02226211 | up | 2.56282E-10 | 3.34642E-09 |
| 9926 | LPGAT1 | 4.156511875 | 2.055373333 | 4.156511875 | up | 7.7491E-17 | 2.24484E-14 |
| 80856 | KIAA1715 | 2.941414294 | 1.55651 | 2.941414294 | up | 1.02389E-13 | 4.53295E-12 |
| 84187 | TMEM164 | 2.445252307 | 1.289983333 | 2.445252307 | up | 8.52898E-10 | 9.67465E-09 |
| 112574 | SNX18 | 2.160552069 | 1.1114 | 2.160552069 | up | 3.543E-10 | 4.45716E-09 |
| 157378 | TMEM65 | 2.286271388 | 1.192996667 | 2.286271388 | up | 6.6058E-12 | 1.42291E-10 |
| 51465 | UBE2J1 | 2.040614033 | 1.029003333 | 2.040614033 | up | 8.44392E-14 | 3.85131E-12 |
| 2744 | GLS | 5.144782189 | 2.36311 | 5.144782189 | up | 7.70725E-22 | 3.02317E-17 |
| 134266 | GRPEL2 | 2.123297709 | 1.086306667 | 2.123297709 | up | 1.66257E-09 | 1.7451E-08 |
| 11320 | MGAT4A | 4.028678466 | 2.010306667 | 4.028678466 | up | 2.13634E-13 | 8.15155E-12 |
| 163 | AP2B1 | 2.051088866 | 1.03639 | 2.051088866 | up | 6.89728E-16 | 9.768E-14 |
| 79762 | C1orf115 | 2.058723386 | 1.04175 | 2.058723386 | up | 4.74911E-11 | 7.63145E-10 |
| 56886 | UGGT1 | 2.428154128 | 1.27986 | 2.428154128 | up | 1.88581E-13 | 7.33839E-12 |
| 3597 | IL13RA1 | 2.252535745 | 1.17155 | 2.252535745 | up | 5.31669E-14 | 2.684E-12 |
| 23365 | ARHGEF12 | 4.081529826 | 2.02911 | 4.081529826 | up | 1.75415E-14 | 1.13542E-12 |
| 51704 | GPRC5B | 2.255895126 | 1.1737 | 2.255895126 | up | 9.39178E-12 | 1.88726E-10 |
| 84679 | SLC9A7 | 2.375113844 | 1.247996667 | 2.375113844 | up | 4.67334E-13 | 1.52887E-11 |
| 22824 | HSPA4L | 2.121150029 | 1.084846667 | 2.121150029 | up | 1.38158E-14 | 9.45766E-13 |
| 64795 | RMND5A | 2.263972865 | 1.178856667 | 2.263972865 | up | 1.79772E-14 | 1.1541E-12 |
| 57700 | FAM160B1 | 4.476682495 | 2.16243 | 4.476682495 | up | 1.50139E-16 | 3.49123E-14 |
| 6288 | SAA1 | 2.655935296 | 1.40922 | 2.655935296 | up | 2.63955E-12 | 6.57791E-11 |
| 5991 | RFX3 | 2.177189022 | 1.122466667 | 2.177189022 | up | 7.03137E-09 | 6.24701E-08 |
| 27314 | RAB30 | 2.49584787 | 1.31953 | 2.49584787 | up | 1.28534E-12 | 3.53311E-11 |
| 9321 | TRIP11 | 2.160242591 | 1.111193333 | 2.160242591 | up | 4.91571E-13 | 1.59091E-11 |
| 10982 | MAPRE2 | 2.031398573 | 1.022473333 | 2.031398573 | up | 1.79689E-10 | 2.44817E-09 |
| 23076 | RRP1B | 2.22633393 | 1.15467 | 2.22633393 | up | 1.46403E-10 | 2.05351E-09 |
| 29767 | TMOD2 | 3.629045135 | 1.85959 | 3.629045135 | up | 2.50505E-11 | 4.36883E-10 |
| 80762 | NDFIP1 | 2.080985145 | 1.057266667 | 2.080985145 | up | 1.51543E-13 | 6.19368E-12 |
| 22862 | FNDC3A | 2.164834396 | 1.114256667 | 2.164834396 | up | 5.89025E-12 | 1.29039E-10 |
| 83464 | APH1B | 3.755955577 | 1.90918 | 3.755955577 | up | 6.87857E-18 | 4.49687E-15 |
| 79832 | QSER1 | 3.091006454 | 1.628076667 | 3.091006454 | up | 4.57148E-15 | 4.17986E-13 |
| 10114 | HIPK3 | 4.541839317 | 2.183276667 | 4.541839317 | up | 1.61224E-14 | 1.05752E-12 |
| 27 | ABL2 | 2.337770543 | 1.225133333 | 2.337770543 | up | 5.03858E-12 | 1.13065E-10 |
| 126731 | CCSAP | 2.031422041 | 1.02249 | 2.031422041 | up | 1.91334E-08 | 1.52088E-07 |
| 23527 | ACAP2 | 4.309777332 | 2.107613333 | 4.309777332 | up | 1.18724E-15 | 1.46838E-13 |
| 9077 | DIRAS3 | 2.115492152 | 1.080993333 | 2.115492152 | up | 1.43256E-09 | 1.53074E-08 |
| 55195 | C14orf105 | 2.18315314 | 1.126413333 | 2.18315314 | up | 4.56251E-10 | 5.5579E-09 |
| 54545 | MTMR12 | 3.950257411 | 1.981946667 | 3.950257411 | up | 1.86037E-15 | 2.0684E-13 |
| 54495 | TMX3 | 2.875348184 | 1.523736667 | 2.875348184 | up | 5.94093E-11 | 9.32132E-10 |
| 23080 | AVL9 | 2.930553821 | 1.551173333 | 2.930553821 | up | 4.29932E-17 | 1.42916E-14 |
| 152006 | RNF38 | 3.471277417 | 1.795466667 | 3.471277417 | up | 1.69334E-12 | 4.49705E-11 |
| 6498 | SKIL | 3.045445598 | 1.606653333 | 3.045445598 | up | 6.90621E-13 | 2.1065E-11 |
| 2113 | ETS1 | 2.691169636 | 1.428233333 | 2.691169636 | up | 1.04976E-10 | 1.52394E-09 |
| 23568 | ARL2BP | 2.379777456 | 1.250826667 | 2.379777456 | up | 1.66911E-16 | 3.74119E-14 |
| 5611 | DNAJC3 | 3.831829406 | 1.938033333 | 3.831829406 | up | 3.23278E-14 | 1.82717E-12 |
| 64426 | SUDS3 | 2.353704708 | 1.234933333 | 2.353704708 | up | 5.72527E-10 | 6.77856E-09 |
| 56138 | PCDHA11 | 2.074240722 | 1.052583333 | 2.074240722 | up | 1.427E-13 | 5.89821E-12 |
| 9732 | DOCK4 | 4.552713476 | 2.186726667 | 4.552713476 | up | 5.46897E-16 | 8.51271E-14 |
| 9991 | PTBP3 | 4.201302503 | 2.070836667 | 4.201302503 | up | 1.34413E-16 | 3.21485E-14 |
| 9857 | CEP350 | 2.13427587 | 1.093746667 | 2.13427587 | up | 2.68778E-12 | 6.6769E-11 |
| 167555 | FAM151B | 2.192449076 | 1.132543333 | 2.192449076 | up | 5.62924E-10 | 6.68301E-09 |
| 5825 | ABCD3 | 2.170182946 | 1.117816667 | 2.170182946 | up | 2.37691E-13 | 8.81954E-12 |
| 128486 | FITM2 | 3.305549227 | 1.72489 | 3.305549227 | up | 3.65902E-13 | 1.25459E-11 |
| 64395 | GMCL1 | 2.182769818 | 1.12616 | 2.182769818 | up | 6.06126E-10 | 7.12262E-09 |
| 142891 | SAMD8 | 4.293090741 | 2.102016667 | 4.293090741 | up | 7.06283E-17 | 2.083E-14 |
| 134353 | LSM11 | 2.681604856 | 1.423096667 | 2.681604856 | up | 8.25556E-12 | 1.70255E-10 |
| 353376 | TICAM2 | 2.15841159 | 1.10997 | 2.15841159 | up | 8.32531E-11 | 1.24594E-09 |
| 23362 | PSD3 | 2.815562807 | 1.493423333 | 2.815562807 | up | 1.23289E-09 | 1.34483E-08 |
| 10116 | FEM1B | 3.048184017 | 1.60795 | 3.048184017 | up | 5.64417E-14 | 2.80738E-12 |
| 4040 | LRP6 | 2.231808641 | 1.158213333 | 2.231808641 | up | 2.40794E-11 | 4.21469E-10 |
| 145282 | MIPOL1 | 2.178975538 | 1.12365 | 2.178975538 | up | 1.04807E-09 | 1.16395E-08 |
| 23657 | SLC7A11 | 3.837535243 | 1.94018 | 3.837535243 | up | 6.56716E-20 | 2.35663E-16 |
| 167227 | DCP2 | 3.08217049 | 1.623946667 | 3.08217049 | up | 1.16179E-11 | 2.27175E-10 |
| 132864 | CPEB2 | 5.380859611 | 2.427836667 | 5.380859611 | up | 2.69912E-18 | 2.52079E-15 |
| 23705 | CADM1 | 2.354145244 | 1.235203333 | 2.354145244 | up | 3.14854E-15 | 3.12662E-13 |
| 9615 | GDA | 2.081951797 | 1.057936667 | 2.081951797 | up | 3.61607E-09 | 3.45615E-08 |
| 26146 | TRAF3IP1 | 2.854174088 | 1.513073333 | 2.854174088 | up | 1.59176E-12 | 4.25608E-11 |
| 22847 | ZNF507 | 2.701717194 | 1.433876667 | 2.701717194 | up | 1.54679E-13 | 6.30693E-12 |
| 7035 | TFPI | 2.037052741 | 1.026483333 | 2.037052741 | up | 1.312E-15 | 1.59328E-13 |
| 5194 | PEX13 | 2.02993003 | 1.02143 | 2.02993003 | up | 4.53469E-13 | 1.49097E-11 |
| 55233 | MOB1A | 2.038111999 | 1.027233333 | 2.038111999 | up | 2.26459E-16 | 4.75019E-14 |
| 23271 | CAMSAP2 | 2.121468612 | 1.085063333 | 2.121468612 | up | 4.11506E-14 | 2.18717E-12 |
| 56143 | PCDHA5 | 2.074240722 | 1.052583333 | 2.074240722 | up | 1.427E-13 | 5.89821E-12 |
| 1824 | DSC2 | 2.56680435 | 1.359973333 | 2.56680435 | up | 8.23941E-12 | 1.70011E-10 |
| 57234 | LINC00869 | 2.742021722 | 1.45524 | 2.742021722 | up | 8.67027E-15 | 6.70792E-13 |
| 375190 | FAM228B | 2.166760957 | 1.11554 | 2.166760957 | up | 3.55272E-11 | 5.92498E-10 |
| 148534 | TMEM56 | 2.846718819 | 1.5093 | 2.846718819 | up | 2.33651E-16 | 4.84919E-14 |
| 9019 | MPZL1 | 2.646984964 | 1.40435 | 2.646984964 | up | 1.09182E-17 | 5.96586E-15 |
| 4179 | CD46 | 2.438662315 | 1.28609 | 2.438662315 | up | 1.39895E-14 | 9.54324E-13 |
| 92014 | SLC25A51 | 2.460520154 | 1.298963333 | 2.460520154 | up | 2.16227E-12 | 5.51105E-11 |
| 55970 | GNG12 | 2.108796674 | 1.07642 | 2.108796674 | up | 4.66467E-14 | 2.41387E-12 |
| 60312 | AFAP1 | 3.092120767 | 1.628596667 | 3.092120767 | up | 6.53045E-12 | 1.40978E-10 |
| 2932 | GSK3B | 2.30530345 | 1.204956667 | 2.30530345 | up | 3.62037E-15 | 3.46363E-13 |
| 5563 | PRKAA2 | 2.960258851 | 1.565723333 | 2.960258851 | up | 1.50712E-11 | 2.81777E-10 |
| 55331 | ACER3 | 2.275978086 | 1.186486667 | 2.275978086 | up | 1.20914E-12 | 3.38293E-11 |
| 26751 | SH3YL1 | 2.254248664 | 1.172646667 | 2.254248664 | up | 1.0919E-09 | 1.20613E-08 |
| 3423 | IDS | 2.638839002 | 1.399903333 | 2.638839002 | up | 5.89104E-11 | 9.25785E-10 |
| 56134 | PCDHAC2 | 2.074240722 | 1.052583333 | 2.074240722 | up | 1.427E-13 | 5.89821E-12 |
| 85363 | TRIM5 | 3.07088964 | 1.618656667 | 3.07088964 | up | 4.62691E-10 | 5.62936E-09 |
| 6801 | STRN | 3.124048101 | 1.643416667 | 3.124048101 | up | 1.05838E-13 | 4.64372E-12 |
| 5814 | PURB | 3.311060411 | 1.727293333 | 3.311060411 | up | 3.72505E-15 | 3.53789E-13 |
| 91782 | CHMP7 | 2.0705442 | 1.05001 | 2.0705442 | up | 1.30047E-11 | 2.48978E-10 |
| 10846 | PDE10A | 2.025193838 | 1.01806 | 2.025193838 | up | 2.20651E-10 | 2.92993E-09 |
| 83737 | ITCH | 2.209122366 | 1.143473333 | 2.209122366 | up | 6.13166E-15 | 5.22858E-13 |
| 8202 | NCOA3 | 2.387438108 | 1.255463333 | 2.387438108 | up | 9.64122E-12 | 1.93342E-10 |
| 27067 | STAU2 | 6.259178559 | 2.645973333 | 6.259178559 | up | 2.63683E-16 | 5.26491E-14 |
| 2649 | NR6A1 | 2.103939666 | 1.073093333 | 2.103939666 | up | 1.47135E-08 | 1.19962E-07 |
| 26298 | EHF | 2.781808934 | 1.476023333 | 2.781808934 | up | 2.58264E-12 | 6.44428E-11 |
| 9517 | SPTLC2 | 2.131112371 | 1.091606667 | 2.131112371 | up | 2.7478E-14 | 1.59677E-12 |
| 2776 | GNAQ | 2.051150475 | 1.036433333 | 2.051150475 | up | 5.73418E-14 | 2.83636E-12 |
| 9908 | G3BP2 | 2.099666215 | 1.07016 | 2.099666215 | up | 2.19374E-15 | 2.35108E-13 |
| 23169 | SLC35D1 | 3.262323838 | 1.7059 | 3.262323838 | up | 1.21741E-13 | 5.20389E-12 |
| 23137 | SMC5 | 2.501794575 | 1.322963333 | 2.501794575 | up | 2.66169E-12 | 6.62887E-11 |
| 4012 | LNPEP | 3.919664029 | 1.97073 | 3.919664029 | up | 5.64721E-16 | 8.55257E-14 |
| 7048 | TGFBR2 | 2.08646878 | 1.061063333 | 2.08646878 | up | 3.90206E-14 | 2.11115E-12 |
| 6777 | STAT5B | 2.580576382 | 1.367693333 | 2.580576382 | up | 1.48505E-11 | 2.78181E-10 |
| 152110 | NEK10 | 3.00502432 | 1.587376667 | 3.00502432 | up | 1.35121E-12 | 3.68065E-11 |
| 79602 | ADIPOR2 | 2.431983319 | 1.282133333 | 2.431983319 | up | 3.2869E-13 | 1.15115E-11 |
| 6289 | SAA2 | 2.655935296 | 1.40922 | 2.655935296 | up | 2.63955E-12 | 6.57791E-11 |
| 26154 | ABCA12 | 2.205195659 | 1.140906667 | 2.205195659 | up | 5.28129E-10 | 6.3282E-09 |
| 196 | AHR | 2.295449458 | 1.198776667 | 2.295449458 | up | 2.35185E-13 | 8.75248E-12 |
| 658 | BMPR1B | 2.098899856 | 1.069633333 | 2.098899856 | up | 8.79992E-11 | 1.306E-09 |
| 374354 | NHLRC2 | 3.19905693 | 1.677646667 | 3.19905693 | up | 4.59823E-16 | 7.66812E-14 |
| 6648 | SOD2 | 2.908717797 | 1.540383333 | 2.908717797 | up | 5.58082E-13 | 1.76396E-11 |
| 105379283 | LOC105379283 | 2.16219504 | 1.112496667 | 2.16219504 | up | 3.04434E-08 | 2.31602E-07 |
| 25898 | RCHY1 | 2.450631109 | 1.293153333 | 2.450631109 | up | 4.00707E-14 | 2.13847E-12 |
| 114800 | CCDC85A | 2.010893487 | 1.007836667 | 2.010893487 | up | 5.60017E-09 | 5.09431E-08 |
| 55076 | TMEM45A | 2.518654677 | 1.332653333 | 2.518654677 | up | 2.08082E-10 | 2.78175E-09 |
| 134430 | WDR36 | 2.107900351 | 1.075806667 | 2.107900351 | up | 2.19096E-13 | 8.32756E-12 |
| 9723 | SEMA3E | 2.061245961 | 1.043516667 | 2.061245961 | up | 2.6284E-11 | 4.55183E-10 |
| 22884 | WDR37 | 2.02829852 | 1.02027 | 2.02829852 | up | 1.03147E-08 | 8.80321E-08 |
| 10090 | UST | 2.008413972 | 1.006056667 | 2.008413972 | up | 2.19145E-09 | 2.23214E-08 |
| 8653 | DDX3Y | 2.201103014 | 1.138226667 | 2.201103014 | up | 1.27351E-12 | 3.51291E-11 |
| 2444 | FRK | 2.67264212 | 1.418266667 | 2.67264212 | up | 5.06085E-12 | 1.13371E-10 |
| 84669 | USP32 | 2.068994772 | 1.04893 | 2.068994772 | up | 8.59926E-09 | 7.48405E-08 |
| 23333 | DPY19L1 | 2.70011964 | 1.433023333 | 2.70011964 | up | 2.04727E-17 | 8.59944E-15 |
| 10564 | ARFGEF2 | 4.226060243 | 2.079313333 | 4.226060243 | up | 1.83361E-16 | 3.99573E-14 |
| 64089 | SNX16 | 2.10381328 | 1.073006667 | 2.10381328 | up | 1.35184E-08 | 1.11282E-07 |
| 10642 | IGF2BP1 | 2.450381987 | 1.293006667 | 2.450381987 | up | 3.63506E-15 | 3.46923E-13 |
| 84910 | TMEM87B | 3.18129318 | 1.669613333 | 3.18129318 | up | 8.90308E-17 | 2.43243E-14 |
| 84725 | PLEKHA8 | 2.349808737 | 1.232543333 | 2.349808737 | up | 7.68039E-10 | 8.81144E-09 |
| 56147 | PCDHA1 | 2.074240722 | 1.052583333 | 2.074240722 | up | 1.427E-13 | 5.89821E-12 |
| 5939 | RBMS2 | 2.913607754 | 1.542806667 | 2.913607754 | up | 1.14933E-16 | 2.96595E-14 |
| 9329 | GTF3C4 | 2.10876744 | 1.0764 | 2.10876744 | up | 4.60174E-14 | 2.40031E-12 |
| 255919 | CNEP1R1 | 2.232814399 | 1.158863333 | 2.232814399 | up | 2.73899E-11 | 4.72042E-10 |
| 5562 | PRKAA1 | 2.909806732 | 1.540923333 | 2.909806732 | up | 1.89293E-12 | 4.94013E-11 |
| 7077 | TIMP2 | 2.38742156 | 1.255453333 | 2.38742156 | up | 6.67808E-15 | 5.53801E-13 |
| 996 | CDC27 | 2.309819312 | 1.20778 | 2.309819312 | up | 1.62219E-15 | 1.86054E-13 |
| 219902 | TMEM136 | 2.710777434 | 1.438706667 | 2.710777434 | up | 1.33018E-14 | 9.20064E-13 |
| 145781 | GCOM1 | 2.017553131 | 1.012606667 | 2.017553131 | up | 2.88347E-14 | 1.66086E-12 |
| 4254 | KITLG | 4.729813615 | 2.241783333 | 4.729813615 | up | 1.11118E-15 | 1.39699E-13 |
| 1362 | CPD | 2.148137984 | 1.103086667 | 2.148137984 | up | 5.00939E-15 | 4.50672E-13 |
| 25937 | WWTR1 | 2.683352647 | 1.424036667 | 2.683352647 | up | 5.6102E-18 | 4.07519E-15 |
| 54520 | CCDC93 | 2.386925161 | 1.255153333 | 2.386925161 | up | 2.49535E-10 | 3.26594E-09 |
| 84102 | SLC41A2 | 2.516566405 | 1.331456667 | 2.516566405 | up | 5.64091E-16 | 8.55257E-14 |
| 79679 | VTCN1 | 2.034263643 | 1.024506667 | 2.034263643 | up | 7.96981E-10 | 9.10232E-09 |
| 57532 | NUFIP2 | 2.002978127 | 1.002146667 | 2.002978127 | up | 1.07527E-08 | 9.12243E-08 |
| 9695 | EDEM1 | 3.342613774 | 1.740976667 | 3.342613774 | up | 2.31757E-13 | 8.65779E-12 |
| 9990 | SLC12A6 | 2.148619473 | 1.10341 | 2.148619473 | up | 3.58834E-07 | 2.13133E-06 |
| 9096 | TBX18 | 2.65288107 | 1.40756 | 2.65288107 | up | 8.73289E-13 | 2.56398E-11 |
| 81617 | CAB39L | 2.673976278 | 1.418986667 | 2.673976278 | up | 8.80808E-12 | 1.79853E-10 |
| 5144 | PDE4D | 3.378032793 | 1.756183333 | 3.378032793 | up | 9.06357E-15 | 6.94372E-13 |
| 10363 | HMG20A | 2.240276287 | 1.163676667 | 2.240276287 | up | 2.68785E-11 | 4.64658E-10 |
| 339745 | SPOPL | 3.115002526 | 1.639233333 | 3.115002526 | up | 4.00496E-14 | 2.13847E-12 |
| 51351 | ZNF117 | 2.547755206 | 1.349226667 | 2.547755206 | up | 2.83348E-11 | 4.87257E-10 |
| 10905 | MAN1A2 | 2.913116369 | 1.542563333 | 2.913116369 | up | 1.30391E-12 | 3.5669E-11 |
| 114882 | OSBPL8 | 4.079851571 | 2.028516667 | 4.079851571 | up | 3.48642E-14 | 1.92884E-12 |
| 4897 | NRCAM | 2.498057468 | 1.320806667 | 2.498057468 | up | 1.79852E-12 | 4.74424E-11 |
| 5795 | PTPRJ | 3.600232306 | 1.84809 | 3.600232306 | up | 1.9141E-11 | 3.45515E-10 |
| 27161 | AGO2 | 2.385811397 | 1.25448 | 2.385811397 | up | 7.46002E-15 | 5.98462E-13 |
| 11160 | ERLIN2 | 2.749132928 | 1.458976667 | 2.749132928 | up | 1.40443E-12 | 3.80973E-11 |
| 121512 | FGD4 | 2.747964437 | 1.458363333 | 2.747964437 | up | 2.43948E-12 | 6.12995E-11 |
| 149041 | RC3H1 | 2.629757921 | 1.39493 | 2.629757921 | up | 6.6267E-11 | 1.02054E-09 |
| 729182 | FAM91A3P | 2.742021722 | 1.45524 | 2.742021722 | up | 8.67027E-15 | 6.70792E-13 |
| 1184 | CLCN5 | 2.054005436 | 1.03844 | 2.054005436 | up | 8.21499E-08 | 5.63541E-07 |
| 221035 | REEP3 | 2.937610933 | 1.554643333 | 2.937610933 | up | 1.10004E-17 | 5.96586E-15 |
| 9530 | BAG4 | 2.332035903 | 1.22159 | 2.332035903 | up | 6.03145E-11 | 9.44069E-10 |
| 54806 | AHI1 | 2.081860403 | 1.057873333 | 2.081860403 | up | 9.54581E-10 | 1.07012E-08 |
| 205564 | SENP5 | 2.257365452 | 1.17464 | 2.257365452 | up | 4.43161E-11 | 7.17119E-10 |
| 10786 | SLC17A3 | 2.125388634 | 1.087726667 | 2.125388634 | up | 9.48627E-09 | 8.1798E-08 |
| 92400 | RBM18 | 2.307482969 | 1.20632 | 2.307482969 | up | 9.55806E-16 | 1.23734E-13 |
| 6397 | SEC14L1 | 2.226416234 | 1.154723333 | 2.226416234 | up | 1.42855E-14 | 9.66574E-13 |
| 3953 | LEPR | 4.538031636 | 2.182066667 | 4.538031636 | up | 7.3772E-14 | 3.47384E-12 |
| 56136 | PCDHA13 | 2.074240722 | 1.052583333 | 2.074240722 | up | 1.427E-13 | 5.89821E-12 |
| 4664 | NAB1 | 2.021449272 | 1.01539 | 2.021449272 | up | 1.29115E-10 | 1.83564E-09 |
| 4734 | NEDD4 | 4.965182226 | 2.311846667 | 4.965182226 | up | 6.60879E-20 | 2.35663E-16 |
| 2764 | GMFB | 2.249274909 | 1.16946 | 2.249274909 | up | 8.40141E-13 | 2.47965E-11 |
| 56145 | PCDHA3 | 2.074240722 | 1.052583333 | 2.074240722 | up | 1.427E-13 | 5.89821E-12 |
| 3998 | LMAN1 | 3.856146868 | 1.94716 | 3.856146868 | up | 2.27174E-18 | 2.28485E-15 |
| 221710 | SMIM13 | 2.305756237 | 1.20524 | 2.305756237 | up | 1.10515E-12 | 3.12317E-11 |
| 157769 | FAM91A1 | 2.742021722 | 1.45524 | 2.742021722 | up | 8.67027E-15 | 6.70792E-13 |
| 9262 | STK17B | 2.290427134 | 1.195616667 | 2.290427134 | up | 4.04677E-10 | 5.00425E-09 |
| 57211 | ADGRG6 | 2.223866215 | 1.15307 | 2.223866215 | up | 2.17708E-12 | 5.54159E-11 |
| 767 | CA8 | 2.371034519 | 1.245516667 | 2.371034519 | up | 2.51794E-13 | 9.23911E-12 |
| 2332 | FMR1 | 3.037722324 | 1.60299 | 3.037722324 | up | 4.94877E-13 | 1.59897E-11 |
| 79884 | MAP9 | 2.882152487 | 1.527146667 | 2.882152487 | up | 6.89323E-13 | 2.10511E-11 |
| 138151 | NACC2 | 3.309630138 | 1.72667 | 3.309630138 | up | 5.43246E-16 | 8.48957E-14 |
| 160760 | PPTC7 | 2.488713229 | 1.3154 | 2.488713229 | up | 2.97052E-10 | 3.81779E-09 |
| 9581 | PREPL | 2.511182128 | 1.328366667 | 2.511182128 | up | 1.50278E-15 | 1.76486E-13 |
| 9917 | FAM20B | 2.76464965 | 1.467096667 | 2.76464965 | up | 1.21045E-12 | 3.38334E-11 |
| 5274 | SERPINI1 | 3.188666761 | 1.672953333 | 3.188666761 | up | 5.02168E-10 | 6.05148E-09 |
| 1104 | RCC1 | 2.117712388 | 1.082506667 | 2.117712388 | up | 3.73801E-13 | 1.27134E-11 |
| 79646 | PANK3 | 2.57698356 | 1.365683333 | 2.57698356 | up | 1.28803E-13 | 5.4443E-12 |
| 56146 | PCDHA2 | 2.074240722 | 1.052583333 | 2.074240722 | up | 1.427E-13 | 5.89821E-12 |
| 259232 | NALCN | 3.167643549 | 1.66341 | 3.167643549 | up | 8.16798E-15 | 6.42062E-13 |
| 23468 | CBX5 | 2.201591289 | 1.138546667 | 2.201591289 | up | 8.24051E-15 | 6.46468E-13 |
| 3364 | HUS1 | 2.017012465 | 1.01222 | 2.017012465 | up | 1.49802E-13 | 6.13999E-12 |
| 164 | AP1G1 | 2.079154066 | 1.055996667 | 2.079154066 | up | 5.70441E-12 | 1.25564E-10 |
| 160897 | GPR180 | 2.51608966 | 1.331183333 | 2.51608966 | up | 6.08421E-14 | 2.97944E-12 |
| 9180 | OSMR | 2.644203722 | 1.402833333 | 2.644203722 | up | 1.9978E-14 | 1.24909E-12 |
| 134429 | STARD4 | 2.426634232 | 1.278956667 | 2.426634232 | up | 7.93224E-09 | 6.96535E-08 |
| 84665 | MYPN | 3.306855488 | 1.72546 | 3.306855488 | up | 2.67758E-13 | 9.73379E-12 |
| 10802 | SEC24A | 2.488764981 | 1.31543 | 2.488764981 | up | 1.10655E-12 | 3.12488E-11 |
| 56144 | PCDHA4 | 2.074240722 | 1.052583333 | 2.074240722 | up | 1.427E-13 | 5.89821E-12 |
| 253260 | RICTOR | 2.067690137 | 1.04802 | 2.067690137 | up | 5.93017E-10 | 6.98951E-09 |
| 3340 | NDST1 | 2.000975265 | 1.000703333 | 2.000975265 | up | 3.04306E-13 | 1.07632E-11 |
| 23008 | KLHDC10 | 2.673346176 | 1.418646667 | 2.673346176 | up | 7.47231E-12 | 1.57887E-10 |
| 5594 | MAPK1 | 2.315467221 | 1.211303333 | 2.315467221 | up | 6.64978E-14 | 3.19263E-12 |
| 65983 | GRAMD3 | 2.042807603 | 1.030553333 | 2.042807603 | up | 1.04515E-08 | 8.90637E-08 |
| 4973 | OLR1 | 4.801693555 | 2.263543333 | 4.801693555 | up | 2.56272E-19 | 5.75269E-16 |
| 79627 | OGFRL1 | 2.221308852 | 1.15141 | 2.221308852 | up | 1.16778E-09 | 1.28094E-08 |
| 23034 | SAMD4A | 2.435120785 | 1.283993333 | 2.435120785 | up | 3.1447E-12 | 7.61896E-11 |
| 6558 | SLC12A2 | 3.078007329 | 1.621996667 | 3.078007329 | up | 9.83452E-18 | 5.67292E-15 |
| 29915 | HCFC2 | 2.409638401 | 1.268816667 | 2.409638401 | up | 5.17494E-12 | 1.15469E-10 |
| 3017 | HIST1H2BD | 2.175464287 | 1.121323333 | 2.175464287 | up | 6.311E-15 | 5.33511E-13 |
| 26148 | C10orf12 | 2.111419631 | 1.078213333 | 2.111419631 | up | 1.39045E-09 | 1.49426E-08 |
| 55088 | CCDC186 | 2.341781817 | 1.227606667 | 2.341781817 | up | 4.40668E-09 | 4.10965E-08 |
| 56135 | PCDHAC1 | 2.074240722 | 1.052583333 | 2.074240722 | up | 1.427E-13 | 5.89821E-12 |
| 55041 | PLEKHB2 | 2.010907425 | 1.007846667 | 2.010907425 | up | 3.24687E-14 | 1.8325E-12 |
| 1956 | EGFR | 3.336502689 | 1.738336667 | 3.336502689 | up | 8.11608E-16 | 1.09777E-13 |
| 1656 | DDX6 | 3.48802408 | 1.80241 | 3.48802408 | up | 4.30077E-13 | 1.42722E-11 |
| 51026 | GOLT1B | 2.116362363 | 1.081586667 | 2.116362363 | up | 5.03441E-14 | 2.56461E-12 |
| 122830 | NAA30 | 2.661679138 | 1.412336667 | 2.661679138 | up | 6.99206E-15 | 5.72576E-13 |
| 51054 | PLEKHA8P1 | 5.545061008 | 2.471203333 | 5.545061008 | up | 1.94033E-14 | 1.21581E-12 |
| 9805 | SCRN1 | 2.22721886 | 1.155243333 | 2.22721886 | up | 8.22681E-13 | 2.44282E-11 |
| 92597 | MOB1B | 4.128411179 | 2.045586667 | 4.128411179 | up | 3.38996E-14 | 1.88879E-12 |
| 55103 | RALGPS2 | 5.407081117 | 2.43485 | 5.407081117 | up | 2.88022E-13 | 1.03175E-11 |
| 92255 | LMBRD2 | 2.456118214 | 1.29638 | 2.456118214 | up | 1.83867E-11 | 3.34672E-10 |
| 284323 | ZNF780A | 3.786994519 | 1.921053333 | 3.786994519 | up | 9.7051E-15 | 7.30677E-13 |
| 3084 | NRG1 | 3.744578498 | 1.904803333 | 3.744578498 | up | 1.2243E-18 | 1.65228E-15 |
| 25923 | ATL3 | 3.769091102 | 1.914216667 | 3.769091102 | up | 2.67634E-19 | 5.75269E-16 |
| 54557 | SGTB | 4.275243721 | 2.096006667 | 4.275243721 | up | 2.45903E-13 | 9.06536E-12 |
| 9962 | SLC23A2 | 4.11122026 | 2.039566667 | 4.11122026 | up | 2.54962E-14 | 1.51529E-12 |
| 8526 | DGKE | 3.20778326 | 1.681576667 | 3.20778326 | up | 1.29883E-11 | 2.48978E-10 |
| 54882 | ANKHD1 | 2.020347325 | 1.014603333 | 2.020347325 | up | 2.35263E-09 | 2.37486E-08 |
| 2634 | GBP2 | 2.266092366 | 1.180206667 | 2.266092366 | up | 6.64657E-12 | 1.42856E-10 |
| 8945 | BTRC | 2.170022498 | 1.11771 | 2.170022498 | up | 8.01603E-13 | 2.38928E-11 |
| 80205 | CHD9 | 2.897194751 | 1.534656667 | 2.897194751 | up | 3.95099E-12 | 9.19747E-11 |
| 10018 | BCL2L11 | 2.738387604 | 1.453326667 | 2.738387604 | up | 1.21185E-09 | 1.32482E-08 |
| 8795 | TNFRSF10B | 2.772799565 | 1.471343333 | 2.772799565 | up | 6.07016E-17 | 1.86791E-14 |
| 8674 | VAMP4 | 2.257052537 | 1.17444 | 2.257052537 | up | 6.93658E-11 | 1.06176E-09 |
| 7543 | ZFX | 2.511031279 | 1.32828 | 2.511031279 | up | 3.04269E-13 | 1.07632E-11 |
| 80124 | VCPIP1 | 3.186766542 | 1.672093333 | 3.186766542 | up | 2.11667E-14 | 1.29653E-12 |
| 55754 | TMEM30A | 2.081182287 | 1.057403333 | 2.081182287 | up | 3.11524E-14 | 1.77094E-12 |
| 100528017 | SAA2-SAA4 | 2.655935296 | 1.40922 | 2.655935296 | up | 2.63955E-12 | 6.57791E-11 |
| 84056 | KATNAL1 | 4.43978557 | 2.15049 | 4.43978557 | up | 2.70817E-13 | 9.80869E-12 |
| 84919 | PPP1R15B | 3.165141505 | 1.66227 | 3.165141505 | up | 8.469E-16 | 1.13766E-13 |
| 56141 | PCDHA7 | 2.074240722 | 1.052583333 | 2.074240722 | up | 1.427E-13 | 5.89821E-12 |
| 135114 | HINT3 | 2.93351425 | 1.55263 | 2.93351425 | up | 1.69656E-14 | 1.10728E-12 |
| 7046 | TGFBR1 | 5.70374704 | 2.51191 | 5.70374704 | up | 8.66088E-15 | 6.70792E-13 |
| 5565 | PRKAB2 | 2.418367227 | 1.274033333 | 2.418367227 | up | 1.62165E-10 | 2.24213E-09 |
| 253827 | MSRB3 | 2.80748828 | 1.48928 | 2.80748828 | up | 5.32273E-15 | 4.71837E-13 |
| 22903 | BTBD3 | 2.017725616 | 1.01273 | 2.017725616 | up | 2.01E-11 | 3.60011E-10 |
| 5578 | PRKCA | 2.6623557 | 1.412703333 | 2.6623557 | up | 4.02092E-12 | 9.34363E-11 |
| 100302736 | TMED7-TICAM2 | 2.15841159 | 1.10997 | 2.15841159 | up | 8.32531E-11 | 1.24594E-09 |
| 6999 | TDO2 | 2.070955662 | 1.050296667 | 2.070955662 | up | 2.97219E-10 | 3.81868E-09 |
| 100129518 | LOC100129518 | 2.908717797 | 1.540383333 | 2.908717797 | up | 5.58082E-13 | 1.76396E-11 |
| 254251 | LCORL | 2.247695598 | 1.168446667 | 2.247695598 | up | 8.16116E-11 | 1.22324E-09 |
| 26060 | APPL1 | 2.251391054 | 1.170816667 | 2.251391054 | up | 2.03232E-10 | 2.72819E-09 |
| 4289 | MKLN1 | 2.20340801 | 1.139736667 | 2.20340801 | up | 6.07627E-15 | 5.19263E-13 |
| 3480 | IGF1R | 2.286144614 | 1.192916667 | 2.286144614 | up | 6.8331E-12 | 1.46304E-10 |
| 375035 | SFT2D2 | 2.859771742 | 1.5159 | 2.859771742 | up | 1.72276E-16 | 3.81781E-14 |
| 8895 | CPNE3 | 2.517566698 | 1.33203 | 2.517566698 | up | 2.44569E-14 | 1.46238E-12 |
| 5519 | PPP2R1B | 2.24371588 | 1.16589 | 2.24371588 | up | 4.86835E-13 | 1.57949E-11 |
| 7204 | TRIO | 2.388751314 | 1.256256667 | 2.388751314 | up | 3.28198E-16 | 6.23404E-14 |
| 1106 | CHD2 | 2.317538544 | 1.212593333 | 2.317538544 | up | 6.6366E-12 | 1.4272E-10 |
| 57609 | DIP2B | 3.271457057 | 1.709933333 | 3.271457057 | up | 3.91513E-16 | 7.01238E-14 |
| 27330 | RPS6KA6 | 2.002038891 | 1.00147 | 2.002038891 | up | 1.35676E-06 | 7.12056E-06 |
| 10447 | FAM3C | 2.270389779 | 1.18294 | 2.270389779 | up | 6.22257E-14 | 3.01706E-12 |
| 2820 | GPD2 | 2.030014454 | 1.02149 | 2.030014454 | up | 4.85842E-11 | 7.78796E-10 |
| 123879 | DCUN1D3 | 2.822179987 | 1.49681 | 2.822179987 | up | 3.05045E-10 | 3.90643E-09 |
| 3934 | LCN2 | 2.163439335 | 1.113326667 | 2.163439335 | up | 3.07153E-13 | 1.08541E-11 |
| 8774 | NAPG | 2.639778109 | 1.400416667 | 2.639778109 | up | 1.90528E-13 | 7.40679E-12 |
| 1825 | DSC3 | 2.719447046 | 1.443313333 | 2.719447046 | up | 9.69714E-11 | 1.42301E-09 |
| 84100 | ARL6 | 2.076883079 | 1.05442 | 2.076883079 | up | 3.65415E-09 | 3.48999E-08 |
| 24145 | PANX1 | 2.219323535 | 1.15012 | 2.219323535 | up | 2.49776E-13 | 9.18227E-12 |
| 23176 | SEPT8 | 2.324091174 | 1.216666667 | 2.324091174 | up | 2.88343E-13 | 1.03196E-11 |
| 4644 | MYO5A | 2.486695745 | 1.31423 | 2.486695745 | up | 2.0658E-16 | 4.40385E-14 |
| 84883 | AIFM2 | 2.413298991 | 1.271006667 | 2.413298991 | up | 5.67006E-15 | 4.93144E-13 |
| 57553 | MICAL3 | 2.044002083 | 1.031396667 | 2.044002083 | up | 3.72122E-10 | 4.6649E-09 |
| 7039 | TGFA | 3.902287305 | 1.96432 | 3.902287305 | up | 3.48819E-16 | 6.45397E-14 |
| 53344 | CHIC1 | 2.482023313 | 1.311516667 | 2.482023313 | up | 1.21163E-11 | 2.34929E-10 |
| 56142 | PCDHA6 | 2.074240722 | 1.052583333 | 2.074240722 | up | 1.427E-13 | 5.89821E-12 |
| 26108 | PYGO1 | 2.117844502 | 1.082596667 | 2.117844502 | up | 3.46387E-09 | 3.32771E-08 |
| 9334 | B4GALT5 | 3.983905243 | 1.994183333 | 3.983905243 | up | 6.62815E-14 | 3.18614E-12 |
| 29880 | ALG5 | 2.317506417 | 1.212573333 | 2.317506417 | up | 7.76524E-16 | 1.05761E-13 |
| 50484 | RRM2B | 4.989089902 | 2.318776667 | 4.989089902 | up | 1.3098E-18 | 1.65228E-15 |
| 9444 | QKI | 2.457895081 | 1.297423333 | 2.457895081 | up | 3.2638E-12 | 7.84933E-11 |
| 51028 | VPS36 | 2.101248321 | 1.071246667 | 2.101248321 | up | 3.48141E-11 | 5.8209E-10 |
| 3977 | LIFR | 2.030844813 | 1.02208 | 2.030844813 | up | 4.37604E-09 | 4.08547E-08 |
| 134957 | STXBP5 | 2.030019145 | 1.021493333 | 2.030019145 | up | 5.95192E-12 | 1.29919E-10 |
| 4026 | LPP | 2.94632518 | 1.558916667 | 2.94632518 | up | 1.47869E-11 | 2.77387E-10 |
| 6774 | STAT3 | 2.101141516 | 1.071173333 | 2.101141516 | up | 8.33162E-15 | 6.51683E-13 |
| 64750 | SMURF2 | 2.188102012 | 1.12968 | 2.188102012 | up | 3.55445E-17 | 1.25606E-14 |
| 23548 | TTC33 | 2.544484377 | 1.347373333 | 2.544484377 | up | 2.85793E-12 | 7.02588E-11 |
| 5937 | RBMS1 | 2.001890875 | 1.001363333 | 2.001890875 | up | 5.56099E-14 | 2.78227E-12 |
| 1612 | DAPK1 | 2.495063731 | 1.319076667 | 2.495063731 | up | 7.05919E-11 | 1.07742E-09 |
| 286205 | SCAI | 3.240290889 | 1.696123333 | 3.240290889 | up | 7.77419E-11 | 1.1715E-09 |
| 440672 | NUDT4P1 | 2.193619549 | 1.133313333 | 2.193619549 | up | 6.21599E-15 | 5.26613E-13 |
| 57827 | C6orf47 | 2.043487379 | 1.031033333 | 2.043487379 | up | 3.16522E-08 | 2.39583E-07 |
| 8760 | CDS2 | 2.742490583 | 1.455486667 | 2.742490583 | up | 5.40683E-17 | 1.69666E-14 |
| 331 | XIAP | 2.590241596 | 1.373086667 | 2.590241596 | up | 4.89224E-12 | 1.10223E-10 |
| 3841 | KPNA5 | 2.79387989 | 1.48227 | 2.79387989 | up | 6.6054E-12 | 1.42291E-10 |
| 11116 | FGFR1OP | 3.045220439 | 1.606546667 | 3.045220439 | up | 2.34089E-13 | 8.71997E-12 |
| 55729 | ATF7IP | 2.164769373 | 1.114213333 | 2.164769373 | up | 2.31173E-13 | 8.65244E-12 |
| 60559 | SPCS3 | 2.443998388 | 1.289243333 | 2.443998388 | up | 1.70437E-16 | 3.79851E-14 |
| 639 | PRDM1 | 2.193614481 | 1.13331 | 2.193614481 | up | 8.18673E-11 | 1.22613E-09 |
| 340481 | ZDHHC21 | 3.058568669 | 1.612856667 | 3.058568669 | up | 2.999E-13 | 1.06554E-11 |
| 122525 | C14orf28 | 2.293911925 | 1.19781 | 2.293911925 | up | 5.34537E-13 | 1.70188E-11 |
| 283551 | LINC01588 | 2.079648924 | 1.05634 | 2.079648924 | up | 7.18618E-12 | 1.52202E-10 |
| 127253 | TYW3 | 2.059974768 | 1.042626667 | 2.059974768 | up | 1.0494E-14 | 7.78125E-13 |
| 1272 | CNTN1 | 2.364043638 | 1.241256667 | 2.364043638 | up | 1.71295E-17 | 7.38355E-15 |
| 80012 | PHC3 | 2.04444606 | 1.03171 | 2.04444606 | up | 9.23573E-09 | 7.97955E-08 |
| 57120 | GOPC | 2.059841505 | 1.042533333 | 2.059841505 | up | 7.32704E-13 | 2.20909E-11 |
| 6400 | SEL1L | 3.092849573 | 1.628936667 | 3.092849573 | up | 1.02874E-15 | 1.32302E-13 |
| 56681 | SAR1A | 2.300217048 | 1.20177 | 2.300217048 | up | 2.08885E-16 | 4.42893E-14 |
| 54165 | DCUN1D1 | 2.599612638 | 1.378296667 | 2.599612638 | up | 5.2257E-14 | 2.64488E-12 |
| 1657 | DMXL1 | 2.530828943 | 1.33961 | 2.530828943 | up | 4.85114E-12 | 1.09423E-10 |
| 283417 | DPY19L2 | 2.170122777 | 1.117776667 | 2.170122777 | up | 3.02599E-10 | 3.87637E-09 |
| 8828 | NRP2 | 3.386973424 | 1.759996667 | 3.386973424 | up | 2.36155E-15 | 2.47018E-13 |
| 2243 | FGA | 2.204446809 | 1.140416667 | 2.204446809 | up | 5.4361E-10 | 6.4871E-09 |
| 83544 | DNAL1 | 2.303988209 | 1.204133333 | 2.303988209 | up | 1.09683E-11 | 2.16632E-10 |
| 30845 | EHD3 | 2.069224244 | 1.04909 | 2.069224244 | up | 1.07647E-10 | 1.55925E-09 |
| 6335 | SCN9A | 2.492465151 | 1.317573333 | 2.492465151 | up | 3.50546E-13 | 1.2104E-11 |
| 139322 | APOOL | 2.716859576 | 1.44194 | 2.716859576 | up | 1.91844E-13 | 7.42851E-12 |
| 84328 | LZIC | 2.23002518 | 1.15706 | 2.23002518 | up | 3.93119E-14 | 2.12106E-12 |
| 51569 | UFM1 | 2.151634957 | 1.105433333 | 2.151634957 | up | 1.17351E-14 | 8.42608E-13 |
| 51274 | KLF3 | 2.029404803 | 1.021056667 | 2.029404803 | up | 1.72724E-11 | 3.17336E-10 |
| 9076 | CLDN1 | 2.297609044 | 1.200133333 | 2.297609044 | up | 6.0954E-17 | 1.86791E-14 |
| 11057 | ABHD2 | 3.279016891 | 1.713263333 | 3.279016891 | up | 2.40744E-15 | 2.50482E-13 |
| 7169 | TPM2 | 3.119792333 | 1.64145 | 3.119792333 | up | 2.72568E-14 | 1.58863E-12 |
| 146050 | ZSCAN29 | 2.265511268 | 1.179836667 | 2.265511268 | up | 3.51958E-10 | 4.43481E-09 |
| 147339 | C18orf25 | 2.902863364 | 1.537476667 | 2.902863364 | up | 2.53751E-13 | 9.30224E-12 |
| 26127 | FGFR1OP2 | 2.070525064 | 1.049996667 | 2.070525064 | up | 5.57751E-14 | 2.78698E-12 |
| 6612 | SUMO3 | 2.440996117 | 1.28747 | 2.440996117 | up | 6.30979E-13 | 1.9473E-11 |
| 126626 | GABPB2 | 2.235953213 | 1.16089 | 2.235953213 | up | 2.11154E-09 | 2.15859E-08 |
| 10413 | YAP1 | 4.15741471 | 2.055686667 | 4.15741471 | up | 1.21406E-18 | 1.65228E-15 |
| 54834 | GDAP2 | 2.024024378 | 1.017226667 | 2.024024378 | up | 5.45549E-11 | 8.64612E-10 |
| 255520 | ELMOD2 | 2.213782264 | 1.146513333 | 2.213782264 | up | 3.8182E-13 | 1.28889E-11 |
| 554236 | DPY19L2P1 | 2.170122777 | 1.117776667 | 2.170122777 | up | 3.02599E-10 | 3.87637E-09 |
| 57708 | MIER1 | 3.380757806 | 1.757346667 | 3.380757806 | up | 1.82554E-13 | 7.17505E-12 |
| 51360 | MBTPS2 | 2.221971019 | 1.15184 | 2.221971019 | up | 8.28742E-13 | 2.45524E-11 |
| 5962 | RDX | 2.132614694 | 1.092623333 | 2.132614694 | up | 6.37967E-10 | 7.46101E-09 |
| 56139 | PCDHA10 | 2.074240722 | 1.052583333 | 2.074240722 | up | 1.427E-13 | 5.89821E-12 |
| 8411 | EEA1 | 2.121659784 | 1.085193333 | 2.121659784 | up | 6.1819E-13 | 1.91688E-11 |
| 170688 | NUDT4P2 | 2.193619549 | 1.133313333 | 2.193619549 | up | 6.21599E-15 | 5.26613E-13 |
| 64783 | RBM15 | 2.051254739 | 1.036506667 | 2.051254739 | up | 2.85955E-08 | 2.1916E-07 |
| 56925 | LXN | 2.263967634 | 1.178853333 | 2.263967634 | up | 1.11197E-10 | 1.60534E-09 |
| 2247 | FGF2 | 3.178853801 | 1.668506667 | 3.178853801 | up | 1.65932E-18 | 1.76676E-15 |
| 4883 | NPR3 | 2.712882695 | 1.439826667 | 2.712882695 | up | 4.74043E-13 | 1.54922E-11 |
| 5420 | PODXL | 2.070151951 | 1.049736667 | 2.070151951 | up | 8.99595E-11 | 1.33207E-09 |
| 79598 | CEP97 | 2.218200847 | 1.14939 | 2.218200847 | up | 1.4913E-10 | 2.08098E-09 |
| 56140 | PCDHA8 | 2.074240722 | 1.052583333 | 2.074240722 | up | 1.427E-13 | 5.89821E-12 |
| 79836 | LONRF3 | 3.385956253 | 1.759563333 | 3.385956253 | up | 1.16372E-13 | 5.02167E-12 |
| 10079 | ATP9A | 3.90281028 | 1.964513333 | 3.90281028 | up | 1.7217E-14 | 1.11996E-12 |
| 81488 | POLR2M | 2.017553131 | 1.012606667 | 2.017553131 | up | 2.88347E-14 | 1.66086E-12 |
| 1282 | COL4A1 | 2.003334504 | 1.002403333 | 2.003334504 | up | 1.57501E-13 | 6.39541E-12 |
| 23586 | DDX58 | 2.953044984 | 1.562203333 | 2.953044984 | up | 5.87529E-15 | 5.05391E-13 |
| 56137 | PCDHA12 | 2.074240722 | 1.052583333 | 2.074240722 | up | 1.427E-13 | 5.89821E-12 |
| 125488 | TTC39C | 2.794641711 | 1.482663333 | 2.794641711 | up | 3.69271E-12 | 8.67345E-11 |
| 253782 | CERS6 | 2.721527599 | 1.444416667 | 2.721527599 | up | 8.34209E-12 | 1.71678E-10 |
| 51124 | IER3IP1 | 2.012301763 | 1.008846667 | 2.012301763 | up | 1.51112E-14 | 1.00806E-12 |
| 9752 | PCDHA9 | 2.074240722 | 1.052583333 | 2.074240722 | up | 1.427E-13 | 5.89821E-12 |
| 9475 | ROCK2 | 2.246568939 | 1.167723333 | 2.246568939 | up | 1.0406E-14 | 7.73063E-13 |
| 2633 | GBP1 | 2.355532657 | 1.236053333 | 2.355532657 | up | 2.50602E-11 | 4.36883E-10 |
| 9575 | CLOCK | 2.175610057 | 1.12142 | 2.175610057 | up | 3.97797E-10 | 4.94097E-09 |
| 63926 | ANKEF1 | 2.371428986 | 1.245756667 | 2.371428986 | up | 1.1242E-12 | 3.17242E-11 |
| 27125 | AFF4 | 4.055294439 | 2.019806667 | 4.055294439 | up | 9.11582E-17 | 2.43243E-14 |
| 11163 | NUDT4 | 2.193619549 | 1.133313333 | 2.193619549 | up | 6.21599E-15 | 5.26613E-13 |
| 54726 | OTUD4 | 2.793724969 | 1.48219 | 2.793724969 | up | 3.24026E-10 | 4.12258E-09 |
| 23405 | DICER1 | 2.648532723 | 1.405193333 | 2.648532723 | up | 1.11223E-14 | 8.12422E-13 |
| 23683 | PRKD3 | 2.015973489 | 1.011476667 | 2.015973489 | up | 1.41056E-12 | 3.81845E-11 |
| 7057 | THBS1 | 2.186464614 | 1.1286 | 2.186464614 | up | 1.97642E-13 | 7.63043E-12 |
| 19 | ABCA1 | 2.452670335 | 1.294353333 | 2.452670335 | up | 1.65634E-13 | 6.64488E-12 |
| 84333 | PCGF5 | 3.625098007 | 1.85802 | 3.625098007 | up | 9.20041E-15 | 7.02113E-13 |
| 80854 | SETD7 | 2.470158015 | 1.304603333 | 2.470158015 | up | 4.85668E-10 | 5.86706E-09 |
| 673 | BRAF | 2.174755681 | 1.120853333 | 2.174755681 | up | 2.21095E-10 | 2.93286E-09 |
| 192669 | AGO3 | 2.571701742 | 1.362723333 | 2.571701742 | up | 1.86013E-12 | 4.86747E-11 |
| 54439 | RBM27 | 2.938160758 | 1.554913333 | 2.938160758 | up | 1.11415E-13 | 4.85062E-12 |
| 50999 | TMED5 | 2.032051078 | 1.022936667 | 2.032051078 | up | 4.02258E-13 | 1.344E-11 |
| 130507 | UBR3 | 2.058185954 | 1.041373333 | 2.058185954 | up | 3.96022E-11 | 6.52414E-10 |
| 51091 | SEPSECS | 2.832697736 | 1.502176667 | 2.832697736 | up | 7.67124E-13 | 2.29523E-11 |
| 56938 | ARNTL2 | 2.260455213 | 1.176613333 | 2.260455213 | up | 2.97956E-15 | 2.98146E-13 |
| 10000 | AKT3 | 2.290824069 | 1.195866667 | 2.290824069 | up | 4.35106E-10 | 5.33362E-09 |
| 5781 | PTPN11 | 2.160766733 | 1.111543333 | 2.160766733 | up | 1.69706E-08 | 1.36801E-07 |
| 11099 | PTPN21 | 2.054086116 | 1.038496667 | 2.054086116 | up | 6.20998E-09 | 5.58905E-08 |
| 57608 | KIAA1462 | 2.894030246 | 1.53308 | 2.894030246 | up | 7.98178E-12 | 1.65837E-10 |
| 54583 | EGLN1 | 2.197007813 | 1.13554 | 2.197007813 | up | 1.04149E-15 | 1.33504E-13 |
| 79071 | ELOVL6 | 3.300641999 | 1.722746667 | 3.300641999 | up | 9.35697E-16 | 1.22786E-13 |
| 5165 | PDK3 | 2.111346456 | 1.078163333 | 2.111346456 | up | 2.71724E-11 | 4.68912E-10 |
| 124491 | TMEM170A | 3.249002079 | 1.699996667 | 3.249002079 | up | 5.10744E-15 | 4.57396E-13 |
| 83988 | NCALD | 2.135666929 | 1.094686667 | 2.135666929 | up | 7.23021E-11 | 1.1001E-09 |
| 323 | APBB2 | 3.967306887 | 1.98816 | 3.967306887 | up | 3.4603E-16 | 6.43272E-14 |
| 9098 | USP6 | 2.068994772 | 1.04893 | 2.068994772 | up | 8.59926E-09 | 7.48405E-08 |
| 57720 | GPR107 | 2.081802682 | 1.057833333 | 2.081802682 | up | 2.35364E-10 | 3.10116E-09 |
| 152078 | PQLC2L | 2.233928999 | 1.159583333 | 2.233928999 | up | 3.86138E-09 | 3.66737E-08 |
| 283464 | GXYLT1 | 2.239913986 | 1.163443333 | 2.239913986 | up | 3.8208E-14 | 2.07817E-12 |
| 84881 | RPUSD4 | 2.103200903 | 1.072586667 | 2.103200903 | up | 1.24183E-11 | 2.40002E-10 |
| 56667 | MUC13 | 2.2011793 | 1.138276667 | 2.2011793 | up | 1.58412E-12 | 4.23856E-11 |
| 440145 | MZT1 | 2.352046637 | 1.233916667 | 2.352046637 | up | 4.97567E-13 | 1.60473E-11 |
| 10529 | NEBL | 2.468628938 | 1.30371 | 2.468628938 | up | 2.79712E-12 | 6.90913E-11 |
| 79937 | CNTNAP3 | 2.215946934 | 1.147923333 | 2.215946934 | up | 7.51486E-12 | 1.58326E-10 |
| 102 | ADAM10 | 3.75198311 | 1.907653333 | 3.75198311 | up | 2.82762E-20 | 1.38642E-16 |
| 3936 | LCP1 | 2.364655473 | 1.24163 | 2.364655473 | up | 3.71727E-09 | 3.54251E-08 |
| 55334 | SLC39A9 | 2.948449872 | 1.559956667 | 2.948449872 | up | 1.29378E-12 | 3.55134E-11 |
| 6812 | STXBP1 | 2.181978168 | 1.125636667 | 2.181978168 | up | 2.11507E-10 | 2.82189E-09 |
| 64282 | PAPD5 | 4.619051612 | 2.207596667 | 4.619051612 | up | 3.62862E-14 | 1.9838E-12 |
| 92181 | UBTD2 | 2.257699277 | 1.174853333 | 2.257699277 | up | 1.28586E-11 | 2.47002E-10 |
| 79853 | TM4SF20 | 2.363661322 | 1.241023333 | 2.363661322 | up | 1.87242E-12 | 4.88985E-11 |
| 23341 | DNAJC16 | 2.505027891 | 1.324826667 | 2.505027891 | up | 2.9299E-08 | 2.23982E-07 |
| 23035 | PHLPP2 | 2.008172685 | 1.005883333 | 2.008172685 | up | 3.37205E-08 | 2.53437E-07 |
| 147407 | SLC25A52 | 2.567350021 | 1.36028 | 2.567350021 | up | 6.26813E-13 | 1.93902E-11 |
| 6533 | SLC6A6 | 2.298437336 | 1.200653333 | 2.298437336 | up | 1.11833E-11 | 2.20545E-10 |
| 5049 | PAFAH1B2 | 3.776299875 | 1.916973333 | 3.776299875 | up | 2.06073E-13 | 7.89378E-12 |
| 51439 | FAM8A1 | 2.633600777 | 1.397036667 | 2.633600777 | up | 2.57208E-12 | 6.42531E-11 |
| 27010 | TPK1 | 4.211672724 | 2.074393333 | 4.211672724 | up | 7.19932E-13 | 2.17728E-11 |
| 64328 | XPO4 | 3.841198892 | 1.941556667 | 3.841198892 | up | 5.22639E-13 | 1.67078E-11 |
| 57182 | ANKRD50 | 2.622658565 | 1.39103 | 2.622658565 | up | 8.08642E-15 | 6.38209E-13 |
| 285605 | DTWD2 | 2.013413278 | 1.009643333 | 2.013413278 | up | 2.82994E-09 | 2.78696E-08 |
| 8476 | CDC42BPA | 2.005969958 | 1.0043 | 2.005969958 | up | 1.42077E-09 | 1.51976E-08 |
| 2674 | GFRA1 | 3.324590538 | 1.733176667 | 3.324590538 | up | 6.74951E-17 | 2.00568E-14 |
| 63971 | KIF13A | 2.511472248 | 1.328533333 | 2.511472248 | up | 1.13463E-10 | 1.63444E-09 |
| 91408 | BTF3L4 | 2.404499567 | 1.265736667 | 2.404499567 | up | 1.15782E-13 | 5.00172E-12 |
| 63929 | XPNPEP3 | 2.759467703 | 1.46439 | 2.759467703 | up | 3.08902E-10 | 3.95325E-09 |
| 160851 | DGKH | 3.584768613 | 1.84188 | 3.584768613 | up | 2.37886E-13 | 8.81954E-12 |
| 127845 | GOLT1A | 2.318379377 | 1.213116667 | 2.318379377 | up | 3.09014E-10 | 3.95339E-09 |
| 133619 | PRRC1 | 2.131594969 | 1.091933333 | 2.131594969 | up | 3.5401E-14 | 1.95303E-12 |
| 257397 | TAB3 | 2.783261893 | 1.476776667 | 2.783261893 | up | 1.79393E-09 | 1.86897E-08 |
| 10818 | FRS2 | 2.445806043 | 1.29031 | 2.445806043 | up | 2.03742E-12 | 5.24395E-11 |
| 23431 | AP4E1 | 2.420648049 | 1.275393333 | 2.420648049 | up | 3.12884E-10 | 3.99638E-09 |
| 102724668 | DPY19L1P2 | 2.70011964 | 1.433023333 | 2.70011964 | up | 2.04727E-17 | 8.59944E-15 |
| 8658 | TNKS | 2.222438248 | 1.152143333 | 2.222438248 | up | 6.54034E-10 | 7.63072E-09 |
| 80006 | TRAPPC13 | 2.219477372 | 1.15022 | 2.219477372 | up | 1.13478E-14 | 8.25823E-13 |
| 64396 | GMCL1P1 | 2.182769818 | 1.12616 | 2.182769818 | up | 6.06126E-10 | 7.12262E-09 |
| 51199 | NIN | 2.850167421 | 1.511046667 | 2.850167421 | up | 8.99658E-13 | 2.61948E-11 |
| 3840 | KPNA4 | 2.508920343 | 1.327066667 | 2.508920343 | up | 1.10585E-14 | 8.10782E-13 |
| 2182 | ACSL4 | 2.910996961 | 1.541513333 | 2.910996961 | up | 6.39864E-15 | 5.38598E-13 |
| 2967 | GTF2H3 | 2.104085505 | 1.073193333 | 2.104085505 | up | 2.12284E-12 | 5.42465E-11 |
| 6782 | HSPA13 | 2.00256629 | 1.00185 | 2.00256629 | up | 5.47576E-13 | 1.73495E-11 |
| 163859 | SDE2 | 2.328627699 | 1.21948 | 2.328627699 | up | 8.64173E-12 | 1.77009E-10 |
| 286148 | DPY19L4 | 2.452704336 | 1.294373333 | 2.452704336 | up | 4.3144E-14 | 2.27463E-12 |
| 30001 | ERO1A | 2.78932621 | 1.479916667 | 2.78932621 | up | 2.97558E-15 | 2.98146E-13 |
| 7182 | NR2C2 | 2.660603143 | 1.411753333 | 2.660603143 | up | 7.61114E-12 | 1.59651E-10 |
| 7456 | WIPF1 | 2.364721036 | 1.24167 | 2.364721036 | up | 2.0608E-10 | 2.76169E-09 |
| 6016 | RIT1 | 2.285986156 | 1.192816667 | 2.285986156 | up | 2.6442E-16 | 5.26491E-14 |
| 92906 | HNRNPLL | 4.410106003 | 2.140813333 | 4.410106003 | up | 7.30895E-14 | 3.45414E-12 |
| 79989 | TTC26 | 2.644839177 | 1.40318 | 2.644839177 | up | 8.12029E-13 | 2.41302E-11 |
| 79776 | ZFHX4 | 2.19006446 | 1.130973333 | 2.19006446 | up | 1.47054E-10 | 2.06081E-09 |
